# A Small Molecule Agonist of Krüppel-Like Factor 15 in Proteinuric Kidney Disease

**DOI:** 10.1681/ASN.0000000000000460

**Published:** 2024-08-12

**Authors:** Yiqing Guo, Nehaben A. Gujarati, Andrew K. Chow, Brock T. Boysan, Robert Bronstein, John C. He, Monica P. Revelo, Navjot Pabla, Robert C. Rizzo, Bhaskar Das, Sandeep K. Mallipattu

**Affiliations:** 1 Division of Nephrology, Department of Medicine, Stony Brook University, Stony Brook, New York; 2 Department of Chemistry, Stony Brook University, Stony Brook, New York; 3 Division of Nephrology, Department of Medicine, Icahn School of Medicine at Mount Sinai, New York, New York; 4 Department of Pathology, University of Utah, Salt Lake City, Utah; 5 Division of Pharmaceutics and Pharmacology, College of Pharmacy and Comprehensive Cancer Center, Ohio State University, Columbus, Ohio; 6 Department of Applied Mathematics and Statistics, Stony Brook University, Stony Brook, New York; 7 Institute of Chemical Biology and Drug Discovery, Stony Brook University, Stony Brook, New York; 8 Laufer Center for Physical and Quantitative Biology, Stony Brook University, Stony Brook, New York; 9 Pharmaceutical Sciences, Long Island University, Brookville, New York; 10 Renal Section, Northport VA Medical Center, Northport, New York

**Keywords:** glomerular disease, podocyte

## Abstract

**Key Points:**

- A human podocyte-based high-throughput screen identified a novel agonist of Krüppel-like factor 15 (BT503), independent of glucocorticoid signaling.
- BT503 demonstrated renoprotective effects in three independent proteinuric kidney murine models.
- BT503 directly binds to inhibitor of nuclear factor kappa-B kinase subunit beta to inhibit NF-κB activation, which, subsequently restores Krüppel-like factor 15 under cell stress.

**Background:**

Podocyte loss is the major driver of primary glomerular diseases such as FSGS. While systemic glucocorticoids remain the initial and primary therapy for these diseases, high-dose and chronic use of glucocorticoids is riddled with systemic toxicities. Krüppel-like factor 15 (KLF15) is a glucocorticoid-responsive gene, which is essential for the restoration of mature podocyte differentiation markers and stabilization of actin cytoskeleton in the setting of cell stress. Induction of *KLF15* attenuates podocyte injury and glomerulosclerosis in the setting of cell stress.

**Methods:**

A cell-based high-throughput screen with a subsequent structure–activity relationship study was conducted to identify novel agonists of KLF15 in human podocytes. Next, the agonist was tested in cultured human podocytes under cell stress and in three independent proteinuric models (LPS, nephrotoxic serum nephritis, and HIV-1 transgenic mice). A combination of RNA sequencing and molecular modeling with experimental validation was conducted to demonstrate the direct target of the agonist.

**Results:**

The high-throughput screen with structure–activity relationship study identified BT503, a urea-based compound, as a novel agonist of KLF15, independent of glucocorticoid signaling. BT503 demonstrated protective effects in cultured human podocytes and in three independent proteinuric murine models. Subsequent molecular modeling with experimental validation shows that BT503 targets the inhibitor of nuclear factor kappa-B kinase complex by directly binding to inhibitor of nuclear factor kappa-B kinase subunit beta to inhibit canonical NF-κB signaling, which, in turn, restores KLF15 under cell stress, thereby rescuing podocyte loss and ameliorating kidney injury.

**Conclusions:**

By developing and validating a cell-based high-throughput screen in human podocytes, we identified a novel agonist for KLF15 with salutary effects in proteinuric murine models through direct inhibition of inhibitor of nuclear factor kappa-B kinase subunit beta kinase activity.

## Visual Abstract

**Figure:**
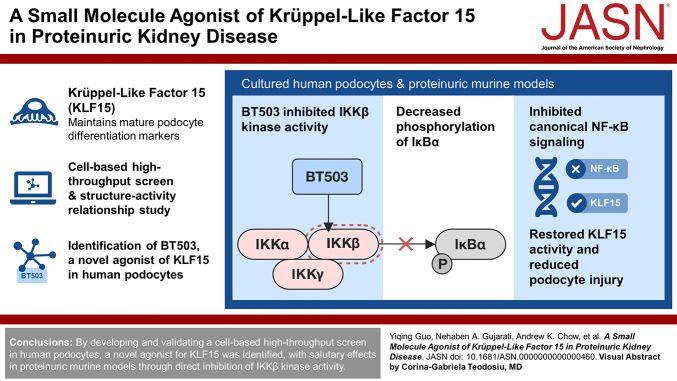

## Introduction

Glucocorticoids are the initial treatment option for many primary glomerulopathies, such as minimal change disease and FSGS.^1^ In many instances, alternate therapy is typically not considered until individuals have not responded to an initial trial of glucocorticoids. In addition, their prolonged use is associated with systemic adverse effects, ranging from weight gain, hyperglycemia, and systemic infections. While the immunomodulatory effects of glucocorticoids are important,^2,3^ the glucocorticoid receptor and the major components of the glucocorticoid receptor complex are expressed with a direct salutary role in human podocytes.^4–9^ Therefore, targeting downstream effectors of glucocorticoid signaling in podocytes might preserve the therapeutic efficacy of glucocorticoids while minimizing its systemic toxicity.

Krüppel-like factor 15 (KLF15) is an early-inducible glucocorticoid-responsive gene, and the knockdown of *KLF15* attenuates the salutary effects of glucocorticoids in human podocytes and in murine models of proteinuric kidney disease.^10^ KLF15 belongs to a subclass of zinc-finger family of DNA-binding transcriptional regulators that are involved in a broad range of cellular processes (*i.e*., cell differentiation, metabolism, and inflammation).^11–15^ Previous studies demonstrate that KLF15 is critical for the maintenance of mature podocyte differentiation markers in human podocytes and in proteinuric murine models.^10,16,17^ The podocyte-specific expression of KLF15 in human kidney biopsies also correlated with responsiveness to glucocorticoids in primary glomerulopathies such as FSGS and minimal change disease.^10^ We also previously demonstrated that the induction of podocyte-specific *KLF15* ameliorated albuminuria, podocyte injury, FSGS, interstitial fibrosis, and overall kidney function in murine models of proteinuric kidney disease.^16^ In this study, we propose to develop a human podocyte-based *KLF15* high-throughput screen to identify a novel small molecule KLF15 agonist with protective effects in models of podocytopathy and proteinuric kidney disease.

## Methods

Additional details regarding materials and procedures, including Supplemental Figure 1, Supplemental Table 1, and computational methods for RNA sequencing, enrichment analysis, and molecular modeling, are provided in the Supplemental Material.

### Cell Culture

Methods for cultivation, immortalization, differentiation, and transduction of cultured human podocytes were based on previously described protocol.^18^

### Mouse Models

Eight-week-old *FVB/n* mice were used for the LPS (Sigma-Aldrich) and nephrotoxic serum (ProbeTex) nephritis models as previously reported.^10,19^ Breeding strategy and use of the 8-week-old hemizygous HIV-1 transgenic (*Tg26)* (*FVB/n*) mice were conducted as previously described.^16^ All mice were administered either BT503 (intraperitoneal, 1 mg/kg) or DMSO vehicle (intraperitoneal, 50% normal saline, 35% PEG300, 5% Tween80, 10% DMSO). Treatment schemas are provided in the respective figures.

**Figure 1:**
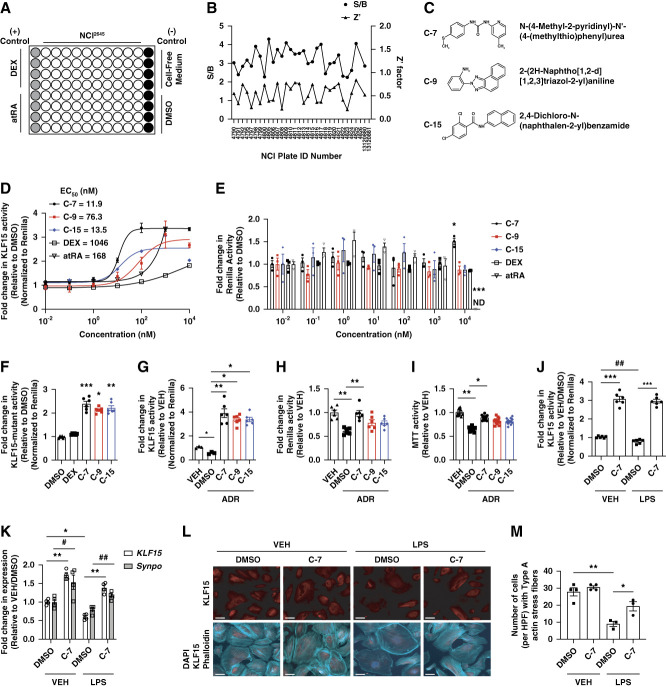
**Identification of KLF15 agonists from NCI**^**2645**^ **high-throughput screen.** (A) Ninety-six–well plate format for NCI^2645^ high-throughput screen. First column, (+) control (DEX-treated and atRA-treated podocytes); last column, (−) control (cell-free medium only, DMSO-treated cells); all other columns, cells treated with NCI^2645^ compounds with a final concentration of 1 *µ*M in 0.5% DMSO. (B) Variations in signal to background (S/B) ratios and Z score (Z′) factors across plates in NCI^2645^ high-throughput screen. S/B ratios and Z′ factors were calculated for each plate and plotted in the graphs. Left, *y* axis, S/B ratios; right, Z′ factors. (C) Chemical structures for C-7, C-9, and C-15. (D) Dose response of KLF15 reporter activity for C-7, C-9, C-15, DEX, and all-trans retinoic acid (relative to DMSO, normalized to renilla). EC_50_ was calculated for each compound. (E) Fold change in renilla activity (relative to DMSO) (*n*=3, *P < 0.05, ***P < 0.001, Kruskal–Wallis test with Dunn post-test, ND—not determined because of cell death). (F) Fold change in KLF15-mutant reporter activity (relative to DMSO, normalized to renilla) (*n*=6, *P < 0.05, **P < 0.01, ***P < 0.001, compared with DEX or DMSO, Kruskal–Wallis test with Dunn post-test). (G) Fold change in KLF15 reporter activity post-adriamycin (0.4 *µ*g/ml) or VEH for 24 hours (relative to VEH-treated podocytes, normalized to renilla) (*n*=6, *P < 0.5, **P < 0.01, Kruskal–Wallis test with Dunn post-test). (H) Fold change in renilla activity (relative to VEH-treated podocytes) (*n*=6, **P < 0.01, Kruskal–Wallis test with Dunn post-test). (I) Fold change in MTT activity (relative to VEH-treated podocytes) (*n*=12, *P < 0.5, **P < 0.01, Kruskal–Wallis test with Dunn post-test). (J) Fold change in KLF15 reporter activity in DMSO-treated and C-7–treated podocytes post-LPS (25 *µ*g/ml) or VEH treatment for 24 hours (relative to VEH/DMSO-treated podocytes, normalized to renilla) (*n*=6, ***P < 0.001, Kruskal–Wallis test with Dunn post-test; ##*P* < 0.01, Mann–Whitney test). (K) Fold change in KLF15 and Synaptopodin (Synpo) expression (relative to VEH/DMSO-treated podocytes) (*n*=4, *P < 0.5, **P < 0.01 [KLF15], #*P* < 0.5, ##*P* < 0.01 [Synpo], Kruskal–Wallis test with Dunn post-test). (L) Immunostaining for phalloidin, KLF15, and Hoechst. Representative images from three independent experiments are shown. (M) Quantification of type A actin stress fibers in DMSO-treated and C-7–treated podocytes (*n*=3–4, *P < 0.5, **P < 0.01, Kruskal–Wallis test with Dunn post-test). atRA, all-trans retinoic acid; DEX, dexamethasone; KLF15, Krüppel-like factor 15; MTT, 3-(4,5-dimethylthiazol-2-yl)-2,5-diphenyltetrazolium bromide.

**Figure 2:**
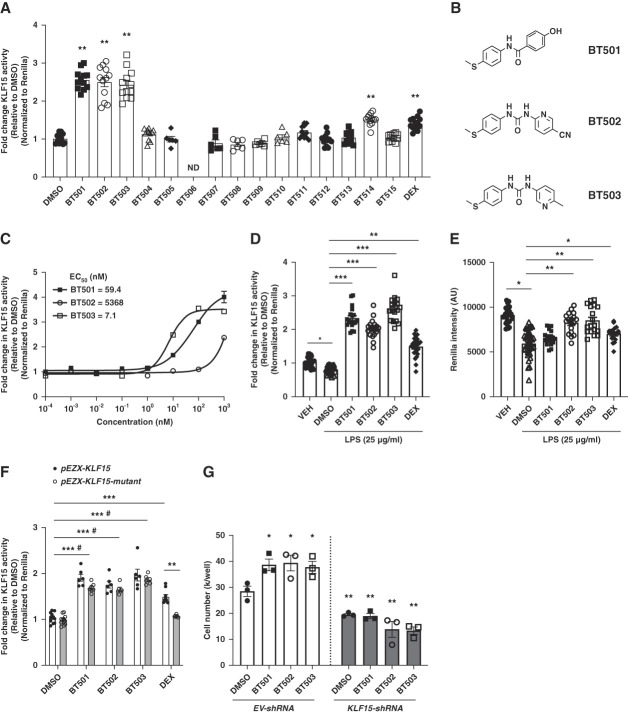
**Novel lead compounds restored KLF15 activity in the setting of podocyte stress.** (A) Fold change in KLF15 reporter activity (relative to DMSO, normalized to renilla, ND—not determined because of low solubility) (*n*=6–12, **P < 0.01, compared with DMSO, Kruskal–Wallis test with Dunn post-test). (B) Chemical structures for BT501, BT502, and BT503. (C) Dose response of KLF15 reporter activity for BT501, BT502, and BT503 (relative to DMSO, normalized to renilla). EC_50_ was calculated for each compound. (D) Fold change in KLF15 reporter activity (relative to DMSO, normalized to renilla) post-LPS (25 *µ*g/ml) or VEH treatment for 24 hours (*n*=18, *P < 0.05, **P < 0.01, ***P < 0.001, Kruskal–Wallis test with Dunn post-test). (E) Renilla intensity (*n*=18, *P < 0.05, **P < 0.01, Kruskal–Wallis test with Dunn post-test). (F) Fold change in *pEZX-KLF15* and *pEZX-KLF15-mutant* reporter activity (relative to DMSO, normalized to renilla) (*n*=6–12, ***P < 0.001 (compared with DMSO, *pEZX-KLF15* activity), #*P* < 0.01 (compared with DMSO, *pEZX-KLF15-mutant* activity), **P < 0.01 (compared with both groups in DEX), two-way ANOVA with Bonferroni post-test). (G) Cell survival measured by cell count for *EV-shRNA* and *KLF15-shRNA* podocytes (*n*=3, *P < 0.05, **P < 0.01, compared with DMSO/*EV-shRNA*, Kruskal–Wallis test with Dunn post-test). EV-shRNA, empty vector short hairpin RNA.

### Statistical Analyses

Based on the normality of the data, the exact test used for each experiment is denoted in the figure legends and expressed as the mean±SEM using GraphPad Prism 9.0. Statistical significance was considered when *P* < 0.05.

### Study Approval

Stony Brook University Animal Institute Committee approved all animal studies, and the National Institutes of Health Guide for the Care and Use of Laboratory Animals was followed strictly.

## Results

### Identification and Validation of Small Molecule KLF15 Agonists from a High-Throughput Screen in Cultured Human Podocytes

To identify small molecule KLF15 agonists in human podocytes, we initially generated human podocytes with a dual reporter, firefly luciferase directed at the *KLF15* promoter region and renilla luciferase (*pEZX-FR03hKLF15p*). Subsequently, we used these *pEZX-FR03hKLF15p* human podocytes to develop a cell-based high-throughput screen in a 96-well layout under nonpermissive conditions (Figure 1A) to screen the National Cancer Institute Drug Screening Sets (2645 compounds) at a final concentration of 1 *μ*M in 0.5% DMSO for 24 hours. The positive controls were inducers of KLF15 expression, dexamethasone (DEX) and all-trans retinoic acid, and media-free and cell-free wells served as negative controls. This high-throughput screen exhibited high reproducibility with low variability as determined by a high signal to background approximately 3.22 and a low Z-score approximately 0.56 (Figure 1B). Using the traditional hit threshold selection methodology,^20^ we initially identified 44 hits with >2.5-fold change in *KLF15* reporter activity (Supplemental Table 2). These initial hits from this primary screen belong to several classes of small molecules, including benzamides, urea analogues, aromatic amines, nitriles, thiols, and dienone. Dose-response studies (concentration range from 0.1 nM to 10 *μ*M) were subsequently conducted for all 44 hit compounds and identified 16 small molecules with EC_50_ <100 nM (Supplemental Table 2). Based on the composition of low-nanomolar EC_50_, stable cell viability, and the Lipinski's rule of five (evaluate druggability and the likelihood of the compound being orally active), we advanced C-7, C-9, and C-15 as our hit KLF15 agonists (Figure 1, C–E, and Supplemental Tables 2 and 3). C-7 showed a significant increase in renilla activity with stable KLF15 activity at higher concentrations (10^4^ nM) as compared with all other groups (Figure 1E). Because glucocorticoid response elements (GRE) occupy the promoter region of *KLF15*,^21–23^ we generated a podocyte dual reporter with a mutation in the GRE (*pEZX-FR03hKLF5p-mutant*) to test whether the induction of KLF15 activity is independent of glucocorticoid receptor signaling. All three KLF15 agonists induced similar KLF15 activity in *pEZX-FR03hKLF5p-mutant* podocytes as compared with DMSO, which was lost with DEX treatment (Figure 1F).

**Figure 3:**
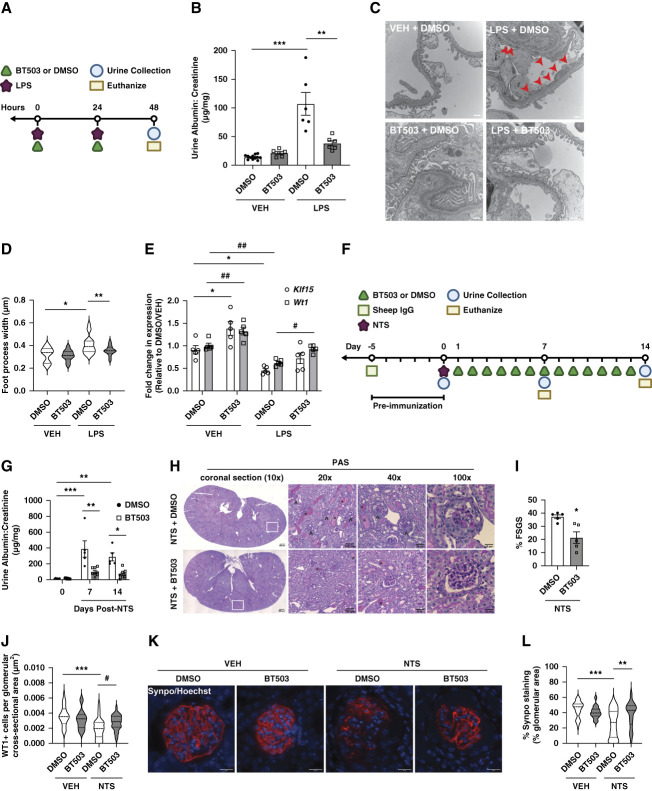
**BT503 attenuated kidney injury in LPS and NTS nephritis proteinuric murine models.** (A) Schematic of the LPS proteinuric model. (B) Urine albumin-creatinine ratio in DMSO-treated and BT503-treated mice in the setting of LPS versus VEH administration (*n*=6–12, **P < 0.01, ***P < 0.001, Kruskal–Wallis test with Dunn post-test). (C) Transmission electron microscopy showing podocyte foot processes in DMSO-treated and BT503-treated mice in the setting of LPS administration. Red arrowheads indicate podocyte effacement. Representative images from three independent experiments are shown. (D) Quantification of FP width (*n*=3, ten glomeruli each, measurements per group, *P < 0.05, **P < 0.01, Kruskal–Wallis test with Dunn post-test). (E) Fold change in *Klf15* and *Wt1* mRNA expression (relative to DMSO/VEH) (*n*=5, *P < 0.05, [*Klf15*]; #*P* < 0.05, ##*P* < 0.01, [*Wt1*], Kruskal–Wallis test with Dunn post-test). (F) Schematic of the NTS nephritis proteinuric model. (G) Urine albumin-creatinine ratio in DMSO-treated, BT503-treated mice post-nephrotoxic serum versus IgG administration (*n*=5–10, *P < 0.05, **P < 0.01, ***P < 0.001, Kruskal–Wallis test with Dunn post-test). (H) Periodic acid–Schiff staining. Representative images are shown at 10×, 20×, 40×, and 100×. White box shows areas of higher magnification. Arrowheads indicate segmentally sclerotic glomeruli; *indicates protein casts. (I) Percentage of glomeruli with FSGS lesions (*n*=5, *P < 0.05, Mann–Whitney test). (J) Podocyte number determined by WT1+ cells per glomerular cross-sectional area (*n*=4 mice, *n*=30 glomeruli per mouse, ***P < 0.001, Kruskal–Wallis test with Dunn post-test; #*P* < 0.05, Mann–Whitney test). (K) Immunostaining for Synaptopodin (Synpo) and Hoechst. Representative images from three different experiments are shown. (L) Quantification of glomerular Synpo expression (% area stained) (*n*=3, 30 glomeruli per mouse, **P < 0.01, ***P < 0.001, Kruskal–Wallis test with Dunn's post-test). FP, foot process; NTS, nephrotoxic serum; VEH, vehicle; Wt1, Wilms tumor 1.

### KLF15 Agonists Attenuated Podocyte Injury under Cell Stress

To determine the salutary effect of these KLF15 agonists (C-7, C-9, and C-15), we initially treated cultured human podocytes with all three agonists as compared with DMSO in the setting of adriamycin. All three agonists induced KLF15 activity as compared wih DMSO-treated podocytes, which showed a reduction in KLF15 activity (Figure 1G). In addition, cell viability (measured by 3-(4,5-dimethylthiazol-2-yl)-2,5-diphenyltetrazolium bromide assay and confirmed by renilla activity) was only maintained with C-7 (Figure 1, H and I). Based on these data, we advanced C-7 to begin *in silico* pharmacokinetic profiling and for further testing in additional *in vitro* and *in vivo* models of podocyte injury. Using the open-source Swiss Institute of Bioinformatics to predict the pharmacokinetics, “leadlikeness,” and medicinal chemistry,^24^ C-7 demonstrated key features consistent with a target-to-hit compound (Supplemental Table 3). In a second model of podocyte injury, C-7 similarly maintained KLF15 activity in the setting of LPS treatment (Figure 1J). In addition, C-7 preserved *KLF15* and *Synaptopodin* expression under LPS conditions (Figure 1K). While LPS reduced KLF15 expression and destabilized the actin cytoskeleton (measured by actin stress fiber formation), immunostaining showed that C-7 restored KLF15 expression and actin stress fiber formation (Figure 1, L and M).

### Novel Structural Analogs of C-7 Restored KLF15 Reporter Activity and Podocyte Viability under Cell Stress

Based on the preceding low EC_50_, “leadlikeness,” and salutary effects in podocytes, we selected C-7 for a structure–activity relationship study to synthesize novel structural analogues. We generated 14 analogs by targeting the three structural moieties of C-7 (Part A contains thiomethyl group of phenyl ring system, Part B contains substituted urea linker, and Part C contains pyridine derivatives) as described in the Supplemental Methods and Supplemental Figure 1. We initially screened these novel analogs and determined that BT501, BT502, and BT503 significantly induced KLF15 reporter activity (>2-fold), with no observed toxicity to human podocytes (Figure 2A). In addition, all three analogs had higher KLF15 activity as compared with DEX-treated podocytes. Subsequent dose escalation studies for these three analogs determined BT503 with EC_50_ approximately 7.1 nM, lowest as compared with the other analogues (Figure 2, B and C). While LPS reduced reporter activity in DMSO-treated podocytes, BT501, BT502, and BT503 maintained the elevated KLF15 activity in the setting of LPS treatment (Figure 2D). However, only BT502 and BT503 restored renilla activity in the setting of LPS treatment (Figure 2E). All three novel analogs induced KLF15 activity in *pEZX-FR03hKLF5p-mutant* podocytes, suggesting these novel compounds induced KLF15 activity independent of glucocorticoid signaling (Figure 2F). To test the specificity of these novel analogues to KLF15, we used previously generated and validated podocytes with KLF15 knockdown (*KLF15-shRNA*) and compared them with control podocytes (empty vector short hairpin RNA).^10^ While all three analogs improved cell survival in empty vector short hairpin RNA podocytes, this was lost in *KLF15-shRNA* podocytes, indicating their salutary effects might be dependent on KLF15 (Figure 2G).

**Figure 4:**
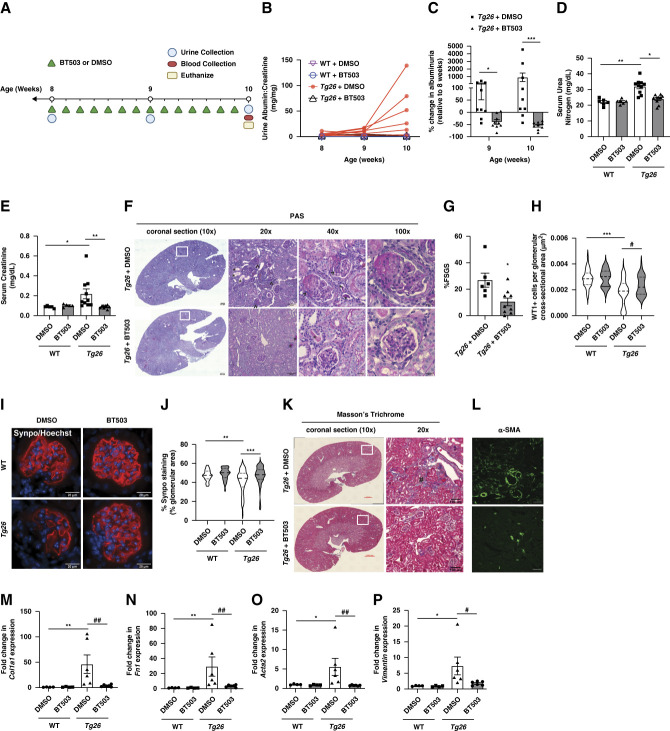
**BT503 attenuated kidney injury in HIV-1 transgenic mice.** (A) Schematic of the HIV-1 transgenic (*Tg26*) proteinuric model. (B) Individual urine albumin-creatinine ratio change over time and (C) % change in urine albumin-creatinine in *Tg26* mice treated with DMSO or BT503 for 2 weeks (relative to albuminuria at 8 weeks) (*n*=9, *P < 0.05, ***P < 0.001, two-way ANOVA). (D) Serum urea nitrogen and (E) serum creatinine (*n*=5–10, *P < 0.05, **P < 0.01, Kruskal–Wallis test with Dunn post-test). (F) Periodic acid–Schiff staining of kidney cortex. Representative images are shown at 10×, 20×, 40×, and 100×. White box shows areas of higher magnification. Arrowheads indicate segmentally sclerotic glomeruli. *indicates protein casts. (G) Percentage of glomeruli with FSGS lesions (*n*=6–9, *P < 0.05, Mann–Whitney test). (H) Podocyte number determined by WT1+ cells per glomerular cross-sectional area (*n*=4 mice, 30 glomeruli per mouse, ***P < 0.001, Kruskal–Wallis test with Dunn post-test; #*P* < 0.05, Mann–Whitney test). (I) Immunostaining for Synaptopodin (Synpo) and Hoechst. Representative images from three different experiments are shown. (J) Quantification of glomerular Synpo expression (% area stained) (*n*=3, 30 glomeruli per mouse, **P < 0.01, ***P < 0.001, Kruskal–Wallis test with Dunn post-test). (K) Masson's trichrome staining of kidney cortex. Representative images are shown at 10× and 20×. White box shows areas of higher magnification. #indicates areas of interstitial fibrosis. (L) Immunostaining for alpha-smooth muscle actin. Representative images from three different experiments are shown. Fold change in (M) *Col1a1*, (N) *Fn1*, (O) *Acta2*, and (P) *Vimentin* mRNA expression (*n*=4–6, *P < 0.05, **P < 0.01, Kruskal–Wallis test with Dunn post-test; #*P* < 0.05, ##*P* < 0.01, Mann–Whitney test).

### BT503 Attenuated Albuminuria and Kidney Disease in Mice

Because BT503 exhibited an EC_50_ <10 nM and improved cell viability in podocytes under cell stress, we advanced BT503 to test its salutary effects *in vivo* using proteinuric murine models. Initially, using the short-term LPS proteinuric murine model^10,17,25,26^ (Figure 3A), concurrent treatment with BT503 attenuated albuminuria as compared with DMSO-treated mice (Figure 3B). In addition, LPS treatment increased foot process effacement (*i.e*., podocyte injury), which was significantly restored in BT503-treated mice (Figure 3, C and D). Glomerular *Klf15* and *Wilms Tumor 1* expression was also restored in BT503-treated mice under LPS conditions (Figure 3E).

To test the therapeutic efficacy of BT503 in additional proteinuric models with progressive kidney disease, we used the nephrotoxic serum nephritis proteinuric model of FSGS^27^ (Figure 3F). BT503-treated mice exhibited significantly less albuminuria as compared with DMSO-treated mice at day 7 and 14 postnephrotoxic serum treatment (Figure 3G). In addition, BT503 significantly reduced the % of glomeruli with FSGS, proteinaceous casts, and restored the podocyte number (Figure 3, H–J). Furthermore, glomerular Synaptopodin expression was restored in BT503-treated mice (Figure 3, K and L).

To further validate these findings, we investigated the efficacy of BT503 in the HIV-1 transgenic (*Tg26*) mice, a model of progressive podocyte loss, severe proteinuria, and collapsing FSGS.^28^ Because significant albuminuria begins at 3–4 weeks and progresses to FSGS lesions by 7–8 weeks of age in the *Tg26* mice, we tested whether treatment with BT503 can mitigate the continued rise in albuminuria and development of FSGS lesions in these mice. We initially selected *Tg26* mice with significant albuminuria (>1 mg/mg urine albumin/creatinine) at 8 weeks of age to ensure likelihood of progressive of kidney disease. Subsequently, these mice were treated with BT503 or vehicle daily for a 14-day period (Figure 4A). We observed that the BT503-treated *Tg26* mice mitigated the increase in albuminuria and improved kidney function (Figure 4, B–E). BT503 also reduced the % of glomeruli with focal segmental and global glomerulosclerosis lesions and preserved podocyte number (Figure 4, F–H). Glomerular Synaptopodin expression was also restored in BT503-treated *Tg26* mice (Figure 4, I and J). In addition, Masson's trichrome staining and immunostaining for alpha-smooth muscle actin showed that treatment with BT503 also reduced interstitial fibrosis (Figure 4, K and L). We also confirmed these findings by measuring cortical mRNA expression of profibrotic markers (*Col1a1*, *Fibronectin 1*, *Acta2*, and *Vimentin*) (Figure 4, M–P).

### BT503 Attenuated Proinflammatory Pathways under Cell Stress

**Figure 5:**
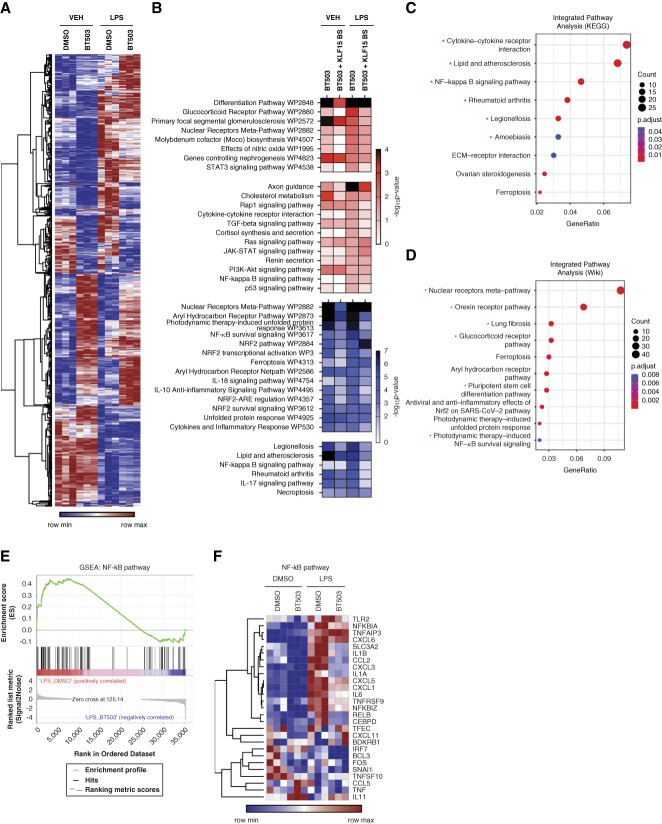
**DEGs with enrichment analysis demonstrated upregulation of genes involved in differentiation and downregulation of genes involved in NF-κB signaling in BT503-treated podocytes.** (A) Heatmap analysis of all 600 DEGs in DMSO-treated, BT503-treated mice in the setting of LPS versus VEH administration. (B) Heatmap analysis of WikiPathway and KEGG Pathway for upregulated (red) and downregulated (blue) DEGs with enrichment for pathways involving genes with KLF15 BS. (C and D) Integrated KEGG pathway and WikiPathway analysis with ClusterProfiler for all DEGs. *Indicates pathways containing DEGs related to NF-κB signaling. (E) GSEA using all DEGs for KEGG NF-κB pathway. (F) Heatmap analysis of DEGs encompassing NF-κB pathway. BS, binding sites; DEG, differentially expressed gene; GSEA, gene set enrichment analysis; KEGG, Kyoto Encyclopedia of Genes and Genomes; SARS-CoV-2, severe acute respiratory syndrome coronavirus 2.

To investigate the potential pathways by which BT503-KLF15 restores podocyte injury, we conducted RNA sequencing in differentiated human podocytes treated with and without BT503 in the setting of LPS treatment (Figure 5A). The ranked upregulated and downregulated differentially expressed genes (DEGs) between the BT503 as compared with DMSO have been provided in Supplemental Tables 4 and 5. We subsequently conducted an enrichment analysis by applying the tool *Enrichr*^29^ to the DEGs (upregulated and downregulated) in the BT503 groups as compared with DMSO (±LPS) using the gene set libraries: WikiPathways^30,31^ and Kyoto Encyclopedia of Genes and Genomes pathways.^32^ Upregulated DEGs post-BT503 treatment revealed a significant increase in pathways involved in cell differentiation, axon guidance, and glucocorticoid signaling (Figure 5B). In comparison, there was an enrichment of nuclear receptors meta-pathway, NF-κB survival signaling pathway, and other inflammatory pathways in the downregulated DEGs (Figure 5B). Cross-matching these DEGs with predicted KLF15 binding sites (BS) in their promoter demonstrated an enrichment of these pathways that might be directly regulated by KLF15, thereby further confirming that the salutary effects of BT503 are in part mediated by KLF15 (Figure 5B).

Because NF-κB signaling has been previously reported to negatively regulate KLF15,^33–35^ we conducted an integrated pathway analysis (Kyoto Encyclopedia of Genes and Genomes and WikiPathways) of both upregulated and downregulated DEGs using *ClusterProfiler* to validate an enrichment of several pathways involving NF-κB signaling (Figure 5, C and D). Gene set enrichment analysis of DEGs involved in NF-κB signaling also showed a downregulation with BT503 as compared with DMSO in the setting of LPS treatment (Figure 5, E and F). To test the potential specificity of BT503 to NF-κB signaling, we tested the conditions that enable the salutary effects of BT503. We observed that BT503 only induces KLF15 activity under nonpermissive (37°C) conditions (*i.e*., differentiated podocytes) as compared with permissive (33°C) conditions (Supplemental Figure 2A). Subsequent RNA sequencing demonstrated a significant enrichment of similar pathways involving cell differentiation from the upregulated DEGs and proinflammatory pathways from the downregulated DEGs post-BT503 treatment under nonpermissive conditions (Supplemental Figure 2, B–D). The ranked upregulated and downregulated DEGs have been provided in Supplemental Tables 6 and 7. Interestingly, *in silico* chromatin immunoprecipitation enrichment analysis^36^ demonstrated an enrichment in putative NFKB1 transcription factor BS occupying the promoter of these DEGs (Supplemental Figure 2E). These data demonstrated that BT503 inhibited proinflammatory pathways, specifically NF-κB signaling, in human podocytes in the setting of cell stress.

### BT503 Directly Inhibited NF-κB Signaling by Targeting Inhibitor of Nuclear Factor Kappa-B Kinase Subunit Beta

**Figure 6:**
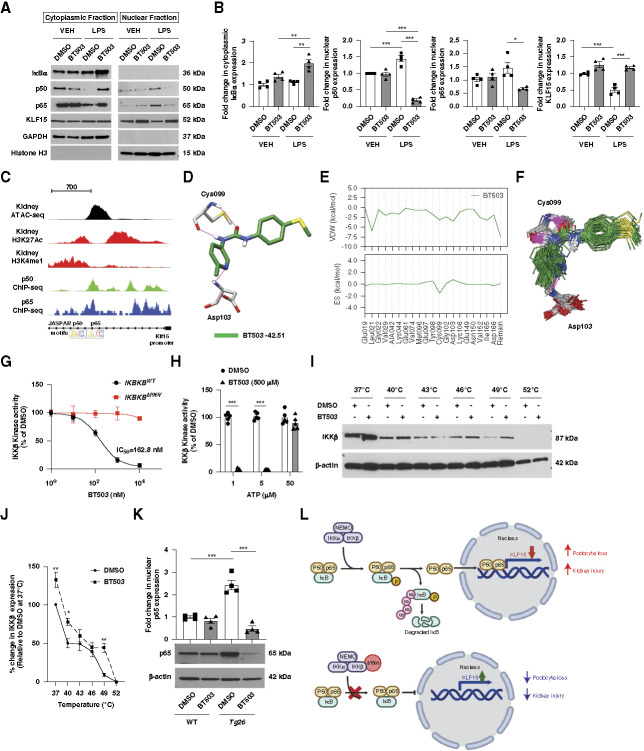
**BT503 inhibited IKK*β* activity, leading to the inhibition of p50/p65 translocation and restoration of KLF15 in the setting of cell stress.** (A and B) Western blot with quantification of densitometry for IκB*α*, p50, p65, KLF15, GAPDH, and Histone H3 from nuclear and cytoplasmic fractions in DMSO-treated versus BT503-treated podocytes in the setting of LPS and VEH treatment. Representative blots from four different experiments are shown (*n*=4, **P < 0.01, ***P < 0.001, Kruskal–Wallis test with Dunn post-test). (C) Mapping of open chromatin from kidney ATAC-seq, H3K27Ac, and H3K4me1 ChIP-seq data from the ENCODE consortium to show putative promoter-proximal enhancer element in the first intron of *KLF15*. Mapping of aforementioned regulatory element to show overlap of ChIP-seq-determined BS for p50 (GSE129618) and p65 (ENCODE ID—ENCSR989LMJ) (canonical motifs are shown for each by the JASPAR motif PWMs listed along the ENSEMBL gene track). (D) Docked BT503 pose (dark green) on IKK*β*. Only two protein residues shown for clarity. Potential H-bonds in magenta. DOCK scores reported in kcal/mol. (E) Molecular footprints (per residue energy breakdown) for docked BT503 (green) with IKK*β*. The VDW (top) and ES (bottom) residue lists correspond to the top 20 most favorable residues. Energies reported in kcal/mol. The 20 highest contributing residues are explicitly shown, and the sum of all other interactions are grouped together into the residue labeled Remain. (F) Ensemble overlay (*N*=100 frames) for BT503 (green) showing key IKK*β* residues (gray) involved in hydrogen bonding (magenta). (G) IKK*β* kinase activity for BT503 and DMSO in *IKBKB*^*WT*^ and *IKBKB*^*M96V*^ (% relative to DMSO). IC_50_ for BT503 in *IKKB*^*WT*^ is shown (***P < 0.001, two-way ANOVA with Bonferroni post-test). (H) IKK*β* kinase activity for BT503 and DMSO at ATP concentration of 1, 5, and 50 *µ*M (% relative to DMSO) (*n*=5, ***P < 0.001, multiple Mann–Whitney tests). (I and J) Western blot with quantification of densitometry for IKK*β* and *β*-actin from the cellular thermal shift assay in human podocytes treated with DMSO or BT503 (1 *µ*M). Representative images of three independent experiments are shown (*n*=4, *P < 0.05, **P < 0.01, two-way ANOVA with Bonferroni post-test). (K) Western blot with quantification of densitometry for p65 and *β*-actin from kidney lysates in DMSO-treated versus BT503-treated *Tg26* mice. Representative blots from four different experiments are shown (*n*=4, ***P < 0.001 compared with all other groups, Kruskal–Wallis test with Dunn post-test). (L) Proposed schematic of (top panel) activated NF-κB signaling suppresses KLF15 expression and subsequent podocyte loss and kidney injury as compared with (bottom panel) BT503-mediated inhibition of IKK*β* inactivates NF-κB signaling, which, subsequently, restores KLF15 expression, leading to a reduction in podocyte loss and kidney injury. ATAC-seq, assay for transposase-accessible chromatin with sequencing; ChIP-seq, chromatin immunoprecipitation sequencing; ENCODE, Encyclopedia of DNA Elements; ES, electrostatic; IKK*β*, inhibitor of nuclear factor kappa-B kinase subunit beta; NEMO, NF-kappa-B essential modifier; PWM, position weight matrix; VDW, Van der Waals.

We initially confirmed that LPS increased the nuclear localization of NF-κB transcription factor dimers (p50 and p65), while the treatment with BT503 mitigated this translocation (Figure 6, A and B). The NF-κB inhibitory subunit, IκB*α*, expression was also increased with BT503 in the setting of LPS treatment, suggesting BT503 inhibits the phosphorylation of IκB*α*, thereby preventing its degradation and subsequent translocation of p50 and p65 from the cytosol to nucleus. In addition, LPS reduced the expression of KLF15, which was restored with BT503 treatment (Figure 6, A and B). We also conducted *in silico* p50 and p65 motif enrichment using p50/p65 ChIP-seq datasets (p50 [GSE129618] and p65 [encyclopedia of DNA elements ID—ENCSR989LMJ]) to demonstrate that p50/p65 BS are located in regions of open chromatin in the putative promoter-proximal enhancer element in the first intron of *KLF15* (Figure 6C), suggesting that the activation of NF-κB signaling directly suppressed *KLF15* expression.

Iκκ complex, composed of inhibitor of nuclear factor kappa-B kinase subunit alpha (IKK*α*), inhibitor of nuclear factor kappa-B kinase subunit beta (IKK*β*), and NF-kappa-B essential modifier/IKKγ, is the central regulator of NF-κB signaling by phosphorylating IκB*α*, with canonical signaling mediated by IKK*β* and noncanonical signaling *via* IKK*α*.^37^ Because BT503 induced KLF15 activity under nonpermissive conditions as compared with permissive conditions (Supplemental Figure 2A), interrogation of DEGs from RNA sequencing of BT503 human podocytes in this setting demonstrated that the key component of Iκκ complex, *IKBKB* (encodes the protein IKK*β*), was significantly upregulated in nonpermissive conditions (*i.e*., differentiated human podocytes) as compared with permissive conditions (Supplemental Figure 2F). Furthermore, IKK*β* protein expression was significantly increased under these nonpermissive conditions as compared with permissive conditions (Supplemental Figure 2, G and H), suggesting the kinase might serve as a target for BT503. Because phospho-IκB*α* is short-lived,^38–40^ we measured the cytoplasmic accumulation of total IκB*α* post-BT503 treatment (Figure 6, A and B) to demonstrate that BT503 targets IKK*β* kinase activity, leading to an inhibition of canonical NF-κB signaling and subsequent restoration of KLF15 expression. Interestingly, IKK*α* levels were not different between both these conditions (Supplemental Figure 2, G and I).

To identify and quantify the most likely binding pose for BT503 with IKK*β*, we initially used previously reported modeling on a related small molecule inhibitor of IKK*β*, INH14,^41^ with a structurally related inhibitor, 1PU.^42^ Quantitative analysis demonstrated binding interactions for BT503 with IKK*β* through energy minimization, docking, molecular footprints, and molecular dynamics (MD) (Figure 6D). This binding pose was also validated for previously reported ligands, INH14 and 1PU, with IKK*β* (Supplemental Figure 3, A and B). Docking BT503 yielded a nearly identical 3D pose to previously reported ligand, INH14, with similar energy scores (Figure 6D and Supplemental Figure 3B). The predicted BT503 pose maintained the favorable electrostatic (ES) interactions between the urea and Cys099, and the rotated pyridine ring places a nitrogen H-bond acceptor within range of Asp103. These docking studies provided strong support for the ligand geometries in Figure 6D and that the current DOCK protocols were suitable for modeling IKK*β*.

To investigate which residues are most likely to contribute to ligand binding in IKK*β*, we decomposed the DOCK scores for BT503 into their respective per-residue Van der Waals and ES components (termed footprints) (Figure 6E). Importantly, the interaction energy profiles were qualitatively similar across all four ligands, which corresponded to the structural overlap observed in Figure 6D and Supplemental Figure 3B.

For computed ES contributions, BT503 had favorable interactions of approximately −2 kcal/mol at position Cys099, which corresponded to the two H-bonds with the protein backbone shown in Figure 6E. Despite the proximity of a potential H-bond acceptor within range of Asp103, BT503 showed a roughly 0.3 kcal/mol ES repulsive at this position (Figure 6E). While the interaction with the backbone was expected to be favorable, the larger overall unfavorable ES interaction with this specific residue could be attributed to repulsion between the BT503 pyridine nitrogen and the Asp103 side chain. Overall, these footprints helped quantify how BT503 could lock into a specific binding geometry with IKK*β*. They also support that the binding was driven by interactions between the trans-diamide and the Cys099 backbone, analogous to those observed in the x-ray structure of 1PU in the CDK4 mimic, which shares homology with IKK*β*.^42^

To further assess the validity of the predictions, MD simulations were performed for each protein–ligand complex. Supplemental Figure 3C plots ligand root mean square deviation (RMSD) as a function of time from solvated IKK*β* complexes with INH14 (orange) and BT503 (green). As a control, the cognate ligand K252a (black) was also simulated to validate the robustness of the MD protocol. Importantly, all the ligand poses were stable in the IKK*β* binding site as judged by plateaued box-averaged RMSD plots versus time (*N*=100 frames, 10 ns) with only minor deviation from their docked (INH14, BT503) or x-ray (K252a) pose. The small deviations for the K252a control (approximately 0.5 Å RMSD, black) demonstrated that the simulation protocols and force field parameters were robust.

An ensemble overlay of structures taken from the BT503 trajectory (every 20th MD frame) was provided to visually assess ligand and sidechain dynamics and the stability of H-bonding (Figure 6F). Here, ligand and sidechains remain tightly locked in place and the consistent cis-trans-urea orientation of BT503 remains pointed at Cys099, which indicated that the two backbone H-bonds observed from docking were maintained during MD. The BT503 pyridine ring nitrogen was oriented toward Asp103, which was also within the H-bonding distance of the protein backbone. The BT503 sulfide was consistently oriented in the general direction of the Asp166 backbone amide (residue not shown), which would also facilitate H-bonding.

To assess how the binding interaction profiles might vary, we also computed time-averaged footprints for 1PU BT502 (Supplemental Figure 3D). The averaged footprints were very similar to those from the single-point docking calculations for BT503 (Figure 6E), and the small error bars indicated good energetic stability over time. Notably for BT503, the slight unfavorable ES interactions originally observed in the docking profiles at Asp103 (and to a lesser extent Tyr098) were relaxed during MD, and Van der Waals packing with Met096 and Tyr098 was improved. The net result was a much tighter overlap between the 1PU BT502 footprint profiles after MD-based sampling.

To validate this interaction between BT503 and IKK*β* experimentally, we initially measured IKK*β* kinase activity in the setting of DMSO BT502 treatment to demonstrate a significant reduction in IKK*β* activity with increasing concentration of BT503 (IC_50_=163 nM) as compared with DMSO (Figure 6G). In addition, this BT503-mediated inhibition of kinase activity was mitigated at higher ATP concentrations (50 *μ*M), suggesting ATP dependency (Figure 6H). To test the specificity of BT503 to IKK*β* inhibition, we generated a *IKBKB* gatekeeper mutant by mutating methionine to valine (*IKBKB*^*M96V*^) in the ATP docking site of IKK*β*. While the *IKBKB*^*M96V*^ mutant has similar kinase activity as compared with *IKBKB*^*WT*^ at baseline, treatment with BT503 demonstrated a resistance to IKK*β* kinase inhibition in the *IKBKB*^*M96V*^ mutant as compared with *IKBKB*^*WT*^ (Figure 6G), thereby suggesting a direct causal link between IKK*β* inhibition and the administration of BT503. Thermal shift assay in human podocytes treated with BT503 or DMSO showed that IKK*β* stability was reduced in DMSO as compared with BT503 treatment, further validating the physical BT503-IKK*β* interaction (Figure 6, I and J). Finally, p65 expression was reduced in BT503-treated *Tg26* mice, confirming that BT503 inhibits NF-κB signaling *in vivo* (Figure 6K). Collectively, these data suggest that BT503 directly inhibited IKK*β* from phosphorylating IκB*α*, NF-κB inhibitory subunit, which prevented the nuclear translocation of NF-κB dimers, and, in turn, restored KLF15 levels under podocyte stress (Figure 6L).

## Discussion

BT503 has a urea linker, which has been commonly found in many clinically used bioactive compounds.^43,44^ Urea moiety also serves as a backbone of many known kinase inhibitors.^45^ Furthermore, a key advantage of urea-based compounds as a therapy for kidney disease has been their ability to form hydrogen bond interactions because of the presence of two hydrogen bond donors and one acceptor, which affects their solubility as well as interactions with target proteins. In addition, the urea linker makes the compounds conformationally restricted,^44^ which helps improve their specificity and potency.^46^ However, there can be limitations; in some instances, having the urea linker has reduced the solubility and permeability of the compound.^47^ Because we showed that the direct target of BT503 was IKK*β*, additional structure–activity relationship studies could be used to generate analogs of BT503, which maintain the predicted H-bonding with Cys099, and enhance efficacy without compromising druggability and toxicity (*i.e*., modifications that enhance interactions with nearby residues Lys044, Tyr098, and Asp103).

Several studies have previously demonstrated the detrimental effects of NF-κB activation in podocyte injury and glomerular disease.^48–52^ In addition, activation of NF-κB signaling and single nucleotide polymorphisms in components of NF-κB signaling have been reported in human glomerular diseases.^53,54^ However, the use of broad NF-κB inhibitors has led to deleterious effects, potentially because of the need for tight regulation of NF-κB signaling in podocytes.^52^ While persistent NF-κB activation caused podocyte injury and inflammation, the complete loss of NF-κB signaling, conversely, has a catastrophic effect because of its essential role in cell survival and anti-apoptotic responses.^52^ Interestingly, our studies showed that human podocytes express higher levels of IKK*β* under nonpermissive versus permissive conditions, suggesting that some residual level of NF-κB signaling might be critical for podocyte differentiation. BT503 also suppressed the expression of only some of the NF-κB target genes, indicating it is not likely a traditional broad inhibitor of NF-κB, but rather provides a calibrated level of NF-κB inhibition. Furthermore, the use of current therapeutic strategies for podocytopathies, such as renin-angiotensin-aldosterone system blockade and glucocorticoids, also inhibited NF-κB signaling to a degree,^55–58^ highlighting the potential significance of calibrated NF-κB inhibition in human glomerular diseases.

The glucocorticoid receptor transcriptionally upregulates KLF15 expression,^10,21,22^ but NF-κB dimers (p50/065) transcriptionally suppress KLF15 expression.^33–35^ In addition, while the same GRE can invoke a positive or negative glucocorticoid effect,^59^ mutating the GRE on KLF15 attenuated the effects of DEX, indicating that this is likely a positive GRE. The BT503-mediated induction of KLF15 activity also remained unaffected, suggesting the effects of BT503 are likely independent of glucocorticoid signaling in podocytes. Furthermore, the effects of other podocyte-specific transcription factors, such as the zinc fingers and homeoboxes (ZHX) family,^60,61^ on KLF15 in the setting of glucocorticoid or NF-κB signaling requires further exploration.

Chemo-resistance occurs in cancer cells because of *de novo* gatekeeper mutations within the target kinases at an ATP docking site, which renders the kinase insensitive to drug inhibition. These gatekeeper mutations have now been used in chemical genetics approaches to examine which targets are critical for a specific biological effect of a particular inhibitor.^62^ Here, we showed a causal link between IKK*β* inhibition and the effects of BT503 by demonstrating that the gatekeeper mutant for IKK*β* has activity that was similar to wild-type IKK*β* at baseline, but resistant to the inhibitory effects of BT503. Interestingly, we also observed that the knockdown of *KLF15* abrogated the salutary effects of BT503 in cultured podocytes, suggesting that the direct effects of BT503-IKK*β* inhibition was mediated through KLF15 in podocytes. Therefore, this novel human podocyte-based KLF15 high-throughput screen could be used to screen additional small molecules that target modulators of KLF15. Finally, the use of BT503 might have a potential therapeutic benefit in other conditions where aberrant activation of canonical NF-κB signaling contributes to disease development and/or progression.

## Supplementary Material

## Data Availability

Raw data from RNA sequencing have been deposited in the Gene Expression Omnibus (accession no. GSE240634), and the reviewer’s access code is cpkfmagwxzkrvoh. https://www.ncbi.nlm.nih.gov/geo/query/acc.cgi?acc=GSE240634.

## Supplemental Material

Supplemental Methods.

Supplemental References.

Supplemental Figure 1. Structure–activity relationship study of C-7 by modifying different core structural moieties and synthetic scheme.

Supplemental Figure 2. BT503 increased KLF15 activity in differentiated human podocytes.

Supplemental Figure 3. Molecular footprints for energy-minimized 1PU, docked 1PU, and INH14 on IKK*β*.

Supplemental Table 1. Primers for real-time PCR.

Supplemental Table 2. Top hits from the KLF15 high-throughput screen in human podocytes.

Supplemental Table 3. Predicted ADME for KLF15 agonists.

Supplemental Table 4. Upregulated DEGs (BT503 versus DMSO [±LPS]).

Supplemental Table 5. Downregulated DEGs (BT503 versus DMSO [±LPS]).

Supplemental Table 6. Upregulated DEGs (BT503 versus DMSO in nonpermissive conditions [37°C], relative to permissive conditions [33°C]).

Supplemental Table 7. Downregulated DEGs (BT503 versus DMSO in nonpermissive conditions [37°C], relative to permissive conditions [33°C]).
