# Supplementary material for: A Small Molecule Agonist of Krüppel-Like Factor 15 in Proteinuric Kidney Disease
Source: J Am Soc Nephrol. 2024 Aug 12;35(12):1671–85. doi: 10.1681/ASN.0000000000000460 (PMC11617484; doi:10.1681/ASN.0000000000000460)
Supplement: Supplementary file 1 [file jasn-35-1671-s001.pdf]

ASN American Society of Nephrology JASN Journal of the American Society of Nephrology KIDNEY360 Accessing Our World From Every Angle CJASN Clinical Journal of the American Society of Nephrology

# ASN Journal Disclosure Form

As per ASN journal policy, I have disclosed any financial relationships or commitments I have held in the past 36 months as included below. I have listed my Current Employer below to indicate there is a relationship requiring disclosure. If no relationship exists, my Current Employer is not listed.

B. Boysan has nothing to disclose.

I understand that the information above will be published within the journal article, if accepted, and that failure to comply and/or to accurately and completely report the potential financial conflicts of interest could lead to the following: 1) Prior to publication, article rejection, or 2) Post-publication, sanctions ranging from, but not limited to, issuing a correction, reporting the inaccurate information to the authors' institution, banning authors from submitting work to ASN journals for varying lengths of time, and/or retraction of the published work.

Name: Brock Boysan
Manuscript ID: JASN-2024-000680R1
Manuscript Title: A small molecule agonist of Krüppel-Like Factor 15 in proteinuric kidney disease
Date of Completion: July 11, 2024
Disclosure Updated Date: July 11, 2024

ASN American Society of Nephrology | JASN Journal of the American Society of Nephrology | KIDNEY360 Accessing Our World From Every Angle | CJASN Clinical Journal of the American Society of Nephrology

# ASN Journal Disclosure Form

As per ASN journal policy, I have disclosed any financial relationships or commitments I have held in the past 36 months as included below. I have listed my Current Employer below to indicate there is a relationship requiring disclosure. If no relationship exists, my Current Employer is not listed.

R. Bronstein has nothing to disclose.

I understand that the information above will be published within the journal article, if accepted, and that failure to comply and/or to accurately and completely report the potential financial conflicts of interest could lead to the following: 1) Prior to publication, article rejection, or 2) Post-publication, sanctions ranging from, but not limited to, issuing a correction, reporting the inaccurate information to the authors' institution, banning authors from submitting work to ASN journals for varying lengths of time, and/or retraction of the published work.

Name: Robert Bronstein
Manuscript ID: JASN-2024-000680R1
Manuscript Title: A small molecule agonist of Krüppel-Like Factor 15 in proteinuric kidney disease
Date of Completion: July 10, 2024
Disclosure Updated Date: May 21, 2024

ASN American Society of Nephrology | JASN Journal of the American Society of Nephrology | KIDNEY360 Accessing Our World From Every Angle | CJASN Clinical Journal of the American Society of Nephrology

# ASN Journal Disclosure Form

As per ASN journal policy, I have disclosed any financial relationships or commitments I have held in the past 36 months as included below. I have listed my Current Employer below to indicate there is a relationship requiring disclosure. If no relationship exists, my Current Employer is not listed.

A. Chow reports the following:
Employer: Stony Brook University

I understand that the information above will be published within the journal article, if accepted, and that failure to comply and/or to accurately and completely report the potential financial conflicts of interest could lead to the following: 1) Prior to publication, article rejection, or 2) Post-publication, sanctions ranging from, but not limited to, issuing a correction, reporting the inaccurate information to the authors' institution, banning authors from submitting work to ASN journals for varying lengths of time, and/or retraction of the published work.

Name: Andrew K Chow
Manuscript ID: JASN-2024-000680R1
Manuscript Title: A small molecule agonist of Krüppel-Like Factor 15 in proteinuric kidney disease
Date of Completion: July 10, 2024
Disclosure Updated Date: May 21, 2024

ASN American Society of Nephrology | JASN Journal of the American Society of Nephrology | KIDNEY360 Accessing Our World From Every Angle | CJASN Clinical Journal of the American Society of Nephrology

# ASN Journal Disclosure Form

As per ASN journal policy, I have disclosed any financial relationships or commitments I have held in the past 36 months as included below. I have listed my Current Employer below to indicate there is a relationship requiring disclosure. If no relationship exists, my Current Employer is not listed.

B. Das reports the following:
Employer: The  Long Island University

I understand that the information above will be published within the journal article, if accepted, and that failure to comply and/or to accurately and completely report the potential financial conflicts of interest could lead to the following: 1) Prior to publication, article rejection, or 2) Post-publication, sanctions ranging from, but not limited to, issuing a correction, reporting the inaccurate information to the authors' institution, banning authors from submitting work to ASN journals for varying lengths of time, and/or retraction of the published work.

Name: Bhaskar Das
Manuscript ID: JASN-2024-000680R1
Manuscript Title: A Small Molecule Agonist of Krüppel-Like Factor 15 in Proteinuric Kidney Disease
Date of Completion: July 24, 2024
Disclosure Updated Date: July 24, 2024

ASN American Society of Nephrology · JASN Journal of the American Society of Nephrology · KIDNEY360 Accessing Our World From Every Angle · CJASN Clinical Journal of the American Society of Nephrology

# ASN Journal Disclosure Form

As per ASN journal policy, I have disclosed any financial relationships or commitments I have held in the past 36 months as included below. I have listed my Current Employer below to indicate there is a relationship requiring disclosure. If no relationship exists, my Current Employer is not listed.

N. Gujarati reports the following:
Employer: Stony Brook University

I understand that the information above will be published within the journal article, if accepted, and that failure to comply and/or to accurately and completely report the potential financial conflicts of interest could lead to the following: 1) Prior to publication, article rejection, or 2) Post-publication, sanctions ranging from, but not limited to, issuing a correction, reporting the inaccurate information to the authors' institution, banning authors from submitting work to ASN journals for varying lengths of time, and/or retraction of the published work.

Name: Nehaben A. Gujarati
Manuscript ID: JASN-2024-000680R1
Manuscript Title: "A small molecule agonist of Krüppel-Like Factor 15 in proteinuric kidney disease"
Date of Completion: July 10, 2024
Disclosure Updated Date: May 20, 2024

ASN American Society of Nephrology
JASN Journal of the American Society of Nephrology
KIDNEY360 Accessing Our World From Every Angle
CJASN Clinical Journal of the American Society of Nephrology

# ASN Journal Disclosure Form

As per ASN journal policy, I have disclosed any financial relationships or commitments I have held in the past 36 months as included below. I have listed my Current Employer below to indicate there is a relationship requiring disclosure. If no relationship exists, my Current Employer is not listed.

Y. Guo reports the following:
Employer: Stony Brook University

I understand that the information above will be published within the journal article, if accepted, and that failure to comply and/or to accurately and completely report the potential financial conflicts of interest could lead to the following: 1) Prior to publication, article rejection, or 2) Post-publication, sanctions ranging from, but not limited to, issuing a correction, reporting the inaccurate information to the authors' institution, banning authors from submitting work to ASN journals for varying lengths of time, and/or retraction of the published work.

Name: Yiqing Guo
Manuscript ID: JASN-2024-000680R1
Manuscript Title: A small molecule agonist of Krüppel-Like Factor 15 in proteinuric kidney disease
Date of Completion: July 10, 2024
Disclosure Updated Date: May 20, 2024

ASN American Society of Nephrology | JASN Journal of the American Society of Nephrology | KIDNEY360 Accessing Our World From Every Angle | CJASN Clinical Journal of the American Society of Nephrology

# ASN Journal Disclosure Form

As per ASN journal policy, I have disclosed any financial relationships or commitments I have held in the past 36 months as included below. I have listed my Current Employer below to indicate there is a relationship requiring disclosure. If no relationship exists, my Current Employer is not listed.

J. He reports the following:
Employer: Icahn School of Medicine at Mount Sinai; Consultancy: Renalytix AI; Yingli Pharmaceutical, Ono Pharmaceutical Co, LTD.; Ownership Interest: Renalytix AI; Rila Therapeutics; Yingli Pharmaceutical.; Research Funding: Shangpharma Innovation; Honoraria: Renalytix AI; Yingli Pharmaceutical, Ono Pharmaceutical Co, LTD.; and Advisory or Leadership Role: Editorial Board for Kidney International; Journal of the American Society of Nephrology, Diabetes, American Journal of Physiology, Board member of Chinese American Society of nephrology and International Chinese Society of Nephrology, Associate Editor for Kidney Disease, Section Editor for Nephron.

I understand that the information above will be published within the journal article, if accepted, and that failure to comply and/or to accurately and completely report the potential financial conflicts of interest could lead to the following: 1) Prior to publication, article rejection, or 2) Post-publication, sanctions ranging from, but not limited to, issuing a correction, reporting the inaccurate information to the authors' institution, banning authors from submitting work to ASN journals for varying lengths of time, and/or retraction of the published work.

Name: John Cijiang He
Manuscript ID: JASN-2024-000680R1
Manuscript Title: A small molecule agonist of Krüppel-Like Factor 15 in proteinuric kidney disease
Date of Completion: July 10, 2024
Disclosure Updated Date: January 10, 2024

ASN American Society of Nephrology
JASN Journal of the American Society of Nephrology
KIDNEY360 Accessing Our World From Every Angle
CJASN Clinical Journal of the American Society of Nephrology

# ASN Journal Disclosure Form

As per ASN journal policy, I have disclosed any financial relationships or commitments I have held in the past 36 months as included below. I have listed my Current Employer below to indicate there is a relationship requiring disclosure. If no relationship exists, my Current Employer is not listed.

S. Mallipattu reports the following:
Employer: Stony Brook Medicine; Consultancy: Wildwood Therapeutics, Inc.; L.E.K. Consulting; Dedham Group; Research Funding: Dialysis Clinic Inc.; Patents or Royalties: Krüppel-like factor 15 (KLF15) Small Molecule Agonists in Kidney Disease. US 63/018.247. April 30, 2021.; and Advisory or Leadership Role: Clinically Integrated Network, Board Member (Accountable Care Organization, LLC Stony Brook Medicine);.

I understand that the information above will be published within the journal article, if accepted, and that failure to comply and/or to accurately and completely report the potential financial conflicts of interest could lead to the following: 1) Prior to publication, article rejection, or 2) Post-publication, sanctions ranging from, but not limited to, issuing a correction, reporting the inaccurate information to the authors' institution, banning authors from submitting work to ASN journals for varying lengths of time, and/or retraction of the published work.

Name: Sandeep K. Mallipattu
Manuscript ID: JASN-2024-000680R1
Manuscript Title: A small molecule agonist of Krüppel-Like Factor 15 in proteinuric kidney disease
Date of Completion: July 10, 2024
Disclosure Updated Date: May 14, 2024

ASN American Society of Nephrology | JASN Journal of the American Society of Nephrology | KIDNEY360 Accessing Our World From Every Angle | CJASN Clinical Journal of the American Society of Nephrology

# ASN Journal Disclosure Form

As per ASN journal policy, I have disclosed any financial relationships or commitments I have held in the past 36 months as included below. I have listed my Current Employer below to indicate there is a relationship requiring disclosure. If no relationship exists, my Current Employer is not listed.

N. Pabla reports the following:
Employer: The Ohio State University

I understand that the information above will be published within the journal article, if accepted, and that failure to comply and/or to accurately and completely report the potential financial conflicts of interest could lead to the following: 1) Prior to publication, article rejection, or 2) Post-publication, sanctions ranging from, but not limited to, issuing a correction, reporting the inaccurate information to the authors' institution, banning authors from submitting work to ASN journals for varying lengths of time, and/or retraction of the published work.

Name: Navjot Pabla
Manuscript ID: JASN-2024-000680R1
Manuscript Title: A small molecule agonist of Krüppel-Like Factor 15 in proteinuric kidney disease
Date of Completion: July 10, 2024
Disclosure Updated Date: May 2, 2024

ASN
American Society of Nephrology
JASN
Journal of the American Society of Nephrology
KIDNEY360
Accessing Our World From Every Angle
CJASN
Clinical Journal of the American Society of Nephrology

# ASN Journal Disclosure Form

As per ASN journal policy, I have disclosed any financial relationships or commitments I have held in the past 36 months as included below. I have listed my Current Employer below to indicate there is a relationship requiring disclosure. If no relationship exists, my Current Employer is not listed.

M. Revelo Penafiel has nothing to disclose.

I understand that the information above will be published within the journal article, if accepted, and that failure to comply and/or to accurately and completely report the potential financial conflicts of interest could lead to the following: 1) Prior to publication, article rejection, or 2) Post-publication, sanctions ranging from, but not limited to, issuing a correction, reporting the inaccurate information to the authors' institution, banning authors from submitting work to ASN journals for varying lengths of time, and/or retraction of the published work.

Name: Monica Patricia Revelo Penafiel
Manuscript ID: (JASN-2024-000680R1
Manuscript Title: A small molecule agonist of Krüppel-Like Factor 15 in proteinuric kidney disease
Date of Completion: July 11, 2024
Disclosure Updated Date: May 15, 2024

ASN
American Society of Nephrology
JASN
Journal of the American Society of Nephrology
KIDNEY360
Accessing Our World From Every Angle
CJASN
Clinical Journal of the American Society of Nephrology

# ASN Journal Disclosure Form

As per ASN journal policy, I have disclosed any financial relationships or commitments I have held in the past 36 months as included below. I have listed my Current Employer below to indicate there is a relationship requiring disclosure. If no relationship exists, my Current Employer is not listed.

R. Rizzo has nothing to disclose.

I understand that the information above will be published within the journal article, if accepted, and that failure to comply and/or to accurately and completely report the potential financial conflicts of interest could lead to the following: 1) Prior to publication, article rejection, or 2) Post-publication, sanctions ranging from, but not limited to, issuing a correction, reporting the inaccurate information to the authors' institution, banning authors from submitting work to ASN journals for varying lengths of time, and/or retraction of the published work.

Name: Robert Rizzo
Manuscript ID: JASN-2024-000680R1
Manuscript Title: "A small molecule agonist of Krüppel-Like Factor 15 in proteinuric kidney disease
Date of Completion: July 16, 2024
Disclosure Updated Date: July 16, 2024
