# Supplementary material for: A Small Molecule Agonist of Krüppel-Like Factor 15 in Proteinuric Kidney Disease
Source: J Am Soc Nephrol. 2024 Aug 12;35(12):1671–85. doi: 10.1681/ASN.0000000000000460 (PMC11617484; doi:10.1681/ASN.0000000000000460)
Supplement: Supplementary file 2 [file jasn-35-1671-s002.pdf]

SUPPLEMENTAL MATERIALS

A small molecule agonist of Krüppel-Like Factor 15 in proteinuric kidney disease

Yiqing Guo[1], Nehaben A. Gujarati[1], Andrew K. Chow[1], Brock Boysan[2], Robert Bronstein[1], John C. He[3], Monica P. Revelo[4], Navjot Pabla[5], Robert C. Rizzo[6,7,8], Bhaskar Das[9], and Sandeep K. Mallipattu[1,10]

Affiliations:
[1]Division of Nephrology, Department of Medicine, Stony Brook University, Stony Brook, NY
[2]Department of Chemistry, Stony Brook University, Stony Brook, NY
[3]Division of Nephrology, Department of Medicine, Icahn School of Medicine at Mount Sinai, New York, NY
[4]Department of Pathology, University of Utah, Salt Lake City, Utah
[5]Division of Pharmaceutics and Pharmacology, College of Pharmacy & Comprehensive Cancer Center, Ohio State University, Columbus, OH
[6]Department of Applied Mathematics & Statistics, Stony Brook University, Stony Brook, NY
[7]Institute of Chemical Biology & Drug Discovery, Stony Brook University, Stony Brook, NY
[8]Laufer Center for Physical & Quantitative Biology, Stony Brook University, Stony Brook, NY
[9]Pharmaceutical Sciences, Long Island University, Brookville, NY
[10]Renal Section, Northport VA Medical Center, Northport, NY

TABLE OF CONTENTS

1. **Supplemental Methods**

2. **Supplemental References**

3. **Supplemental Figures:**
   Supplemental Figure 1: Structure-activity relationship study of C-7 by modifying different core structural moieties and synthetic scheme
   Supplemental Figure 2: BT503 increased KLF15 activity in differentiated human podocytes
   Supplemental Figure 3: Molecular footprints for energy-minimized 1PU, docked 1PU, and INH14 on IKKβ

4. **Supplemental Tables:**
   Supplemental Table 1: Primers for real-time PCR
   Supplemental Table 2: Top hits from the KLF15 High-Throughput Screen in Human Podocytes
   Supplemental Table 3: Predicted ADME for KLF15 Agonists
   Supplemental Table 4: Upregulated DEGs (BT503 vs. DMSO (+/- LPS))
   Supplemental Table 5: Downregulated DEGs (BT503 vs. DMSO (+/- LPS))
   Supplemental Table 6: Upregulated DEGs (BT503 vs. DMSO in nonpermissive conditions (37°C), relative to permissive conditions (33°C))
   Supplemental Table 7: Downregulated DEGs (BT503 vs. DMSO in nonpermissive conditions (37°C), relative to permissive conditions (33°C))

SUPPLEMENTAL METHODS

High-throughput drug screening in human podocytes

Immortalized human podocytes were provided by Dr. Moin Saleem (University of Bristol, Southmead Hospital, Bristol, UK) and methods for cultivation, immortalization, and differentiation were based on previously described protocol.[1] Briefly, these podocytes proliferate under permissive conditions (gamma interferon at 33°C), but differentiate under nonpermissive conditions (37°C). Podocytes in 37°C for 14 days are noted to be differentiated.[1]

To generate the human *KLF15* reporter assay, a 3636bp fragment of the human *KLF15* promoter upstream of the ATG start codon was amplified by PCR on the RP11 71E19 BAC clone (ResGen Invitrogen). The amplified fragment contains MluI and PmeI RE sites and was subcloned into the MluI and PmeI cloning site of the pEZX-FR03 Firefly luciferase (FLuc) and Renilla luciferase (Rluc) reporter cloning vector (GeneCopoeia) to generate *pEZX-FR03hKLF15p*. Following verification by sequencing, the *pEZX-FR03hKLF15p* construct (which contains a puromycin resistance cassette) was transfected into human podocytes followed by selection in puromycin-containing media for 2 weeks. Surviving podocytes clones were pooled together for further analysis.

KLF15 reporter human podocytes were proliferated under permissive conditions (33°C) in RPMI 1640 with 5% fetal bovine serum and 1% penicillin/streptomycin supplemented with 1 μg/mL puromycin, and shifted to 37°C to induce differentiation according to published protocols.[2,3] After 7 days at 37°C, cells were trypsinized, counted, and transferred to 96 white plates (Corning 3917), and they were further cultured at 37°C for another 4 days and subsequently used in the high-throughput screen.[4]

The library of compounds from the National Cancer Institute (Approved Oncology Drugs Set VI; Natural Products Set III; Diversity Set V; and Mechanistic Diversity Set II) were added to a final concentration of 1 μM in 0.5% DMSO in the low FBS cell culture media (0.2% FBS) using liquid handling system PipetMax (Gilson). After 24 hours incubation at 37°C, the Luc-Pair™ Duo-Luciferase HT Assay Kit (GeneCopoeia LF015) were used and firefly and renilla luciferase activity was determined using the SpectraMax M3 (Molecular Devices). Positive controls were all-trans retinoic acid (1 μM) and dexamethasone (1 μM). Negative controls were DMSO and cell-free media. The data were validated by two independent variables: signal-to-background (S/B) ratio and Z' factor as previously reported.[5]

Half maximal effective concentration (EC_{50})

A serial dilution was initially created to measure the half maximal effective concentration (EC_{50}) with concentrations ranging from 10^{-2} to 10^{4} nM. *pEZX-FR03hKLF5p* human podocytes were subsequently treated with various concentrations for each compound and firefly and renilla luciferase activity were measured. The EC_{50} was determined using nonlinear regression and then choosing the equation of Sigmoidal function, 4PL method (X is log(concentration); or the equation of log(agonist) vs. response -- variable slope).

Glucocorticoid response element-mutant KLF15 reporter assay

Using Site-Directed Mutagenesis (Thermo Fisher Scientific), glucocorticoid response element (GRE)[6] was mutated (from GTTATcaattacatgttgtTCctgcAGCC to GTTATgttaatgtctggtaTCtcctAGCC) in the *KLF15* promoter and validated by sequencing. *pEZX-FR03hKLF5p-mutant* (for GRE-mutated human *KLF15* promoter) human podocyte lines were created using transfection and puromycin selection and maintained as described above. Selected hits of National cancer institute (NCI) compounds were added for 24 hours and luciferase activity was measured as described above.

Lipopolysaccharide treatment in human podocytes

Differentiated *pEZX-FR03hKLF5p* human podocytes were treated with 25 µg/ml lipopolysaccharide (LPS) or vehicle and 1 µM KLF15 agonist or vehicle control for 24 hours. Firefly and renilla luciferase assay were measured initially to determine relative KLF15 reporter activity.

Adriamycin treatment in human podocytes

Differentiated *pEZX-FR03hKLF5p* human podocytes were treated with 0.4 µg/ml adriamycin (ADR) or vehicle and 1 µM KLF15 agonist or vehicle control for 24 hours. Firefly and renilla luciferase assay were measured initially to determine relative KLF15 reporter activity.

3- (4, 5-dimethylthiazolyl-2)-2, 5-diphenyltetrazolium bromide (MTT) assay

To assess cell viability, the CellTiter 96® AQueous One Solution Reagent (Promega) was added to the culture media and plates were incubated for 2 hours at 37°C with 5% CO_2. Optical density was determined at 490 nm using a 96-well plate reader SpectraMax M3 (Molecular Devices).

Actin stress fiber formation

To determine actin stress fiber formation, differentiated human podocytes were treated with 25 µg/ml LPS or vehicle and 1 µM KLF15 agonist or vehicle control for 24 hours. Subsequently, podocytes were fixed and stained for F-actin using Alexa Fluor 647 Phalloidin (Life Technologies). Fixation, permeabilization, and staining with phalloidin were performed as per the manufacturer’s protocol. Quantification and classification of changes in actin cytoskeleton are on the basis of previously published methodology [2]. Briefly, phalloidin staining pattern in each cell was classified into the following types: Type A (90% of cell area filled with thick cables), Type B (no thick cables but some cables present), and Type C (no cables visible in the central area of the cell). Unless specified, 100-200 cells were quantified in a blinded manner for each group in three independent experiments.

Immunocytochemistry

Differentiated human podocytes were initially washed with phosphate-buffered saline (PBS) and subsequently fixed with 3.7% formaldehyde in the growth medium. Podocytes were washed and permeabilized with 0.25% Triton X-100, and then blocked in 10% normal horse serum (NHS) and incubated with rabbit anti-KLF15 antibody overnight. The next day, cells were washed with PBS and incubated in Donkey anti-Rabbit IgG (H+L) Secondary Antibody, Alexa Fluor™ 568 (Invitrogen) in 10% NHS. Subsequently, cells were washed and incubated with Hoechst (Thermo Fisher Scientific) before mounting.

Structure-activity relationship study and synthesis of novel analogues of C-7

After identifying C-7 as the lead molecule, we designed a series of analogues (BT501-514) based on the structure-activity relationship to C-7. We synthesized BT502 and BT503 by modifying the pyridine ring in Part C (general schema is shown in **Supplemental Figure 1A**). In BT510 (oxadiazole linker) and BT511 (amide linker), we exploited the role of boron chemistry in drug development to introduce a boronic ester group in place of the methyl group and replaced the pyridine ring with a phenyl ring. Analogues BT501 and BT513 were synthesized

by replacing the urea linker (Part B) with urea, amide (BT501), and oxadiazole (BT513) isosteres to mitigate the effects of urea hydrolysis by urease and thereby prevent toxicity and off-target effects of urea hydrolysis [7]. Additionally, we also replaced the pyridine ring with phenol in the part C for BT501 as well as BT513. Subsequently, we synthesized new analogs by substituting the Part A ring with imidazopyridine derivatives to generate analogues BT504, BT509-511, and BT514 with different linkers (Part B). Finally, a defragmented approach was conducted to synthesize analogues BT505-508 and BT512. These compounds were designed to increase the cell membrane permeability and KLF15 specificity.

For synthesis of the amide linker analogue, BT501, and 4-hydroxybenzoic acid were stirred with 1,1'-carbonyldiimidazole (CDI) in DMF at room temperature for 1 hours followed by addition of 4-(methylsulfanyl)aniline (**1**) and stirring the reaction at 70°C for 30 minutes and 100°C for 12 hours under nitrogen atmosphere **(Supplemental Figure 1B)**. The urea linker analogues (BT502, 503), (4-isocyanatophenyl)(methyl)sulfane (**3**) was synthesized by dropwise addition of triphosgene (**2**) to 4-(methylsulfanyl)aniline (**1**) in dichloromethane (DCM), followed by addition of triethylamine, the reaction mixture was stirred for 8 hours at room temperature to obtain the intermediate (**3**). Corresponding amines (**4**) were added to the intermediate (**3**) in DCM and the reaction mixture was stirred for 12 hours at room temperature to obtain the corresponding analogues **(Supplemental Figure 1C)**. For synthesis of analogues containing oxadiazole moiety, corresponding imidazo[1,2-a]pyridine-3-carboxylic acid (**6**) and 1,1'-carbonyldiimidazole (CDI) were stirred in DMF at 70°C for 30 minutes followed by addition of amidoxime derivatives (**7**), the reaction mixture was then stirred at 100°C for 12 hours to obtain corresponding analogues (BT504, BT509, BT510, and BT514) **(Supplemental Figure 1D)**. BT513 was synthesized using, 4-(methylthio)benzoic acid (**9**) and 1,1'-carbonyldiimidazole (CDI), the reaction mixture was stirred in DMF at 70°C for 30 minutes followed by addition of N,4-dihydroxybenzenecarboximidamide (**10**), and additionally stirring at 100°C for 12 hours **(Supplemental Figure 1E)**. For synthesis of amidoxime analogues (BT506, 507 and 508), substituted nitriles (**11**, **12**, **13**), hydroxylammonium chloride and di-isopropyl ethylamine were refluxed in ethanol for 12 hours to obtain desired analogues **(Supplemental Figure 1F)**. To obtain compounds with the amide linkers, a mixture of 6-chloroimidazo[1,2-a]pyridine-3-carboxylic acid (**14**) and CDI were stirred at room temperature in dry DMF for 1 hour, followed by addition of substituted amines (**15**) under nitrogen atmosphere at stirring at 60°C for 30 minutes.

The resulting mixture was then filtered to remove salt, and further stirred at room temperature for 12 hours to obtain desired analogues (BT505, BT511) **(Supplemental Figure 1G)**.

BT503 treatment in proteinuric murine models

In the LPS-induced proteinuric model, 8-week-old *FVB/n* mice were initially treated with LPS [intraperitoneal (IP), 10 μg/g, Sigma-Aldrich] or sterile normal saline (IP) at 0- and 24-hour time points as previously described.[2] BT503 (IP, 1 mg/kg) or DMSO vehicle (IP, 50% normal saline, 35% PEG300, 5% Tween80, 10% DMSO) were concurrently administered at similar time points. Urine was collected and mice were euthanized 48 hours post-LPS treatment **(Figure 3A)**.

In the nephrotoxic serum nephritis model, 8-week-old *FVB/n* mice were initially sensitized with an intraperitoneal injection of 0.5 mg of sheep IgG (IP, Jackson ImmunoResearch) with complete Freund's adjuvant (IP, Millipore Sigma). Five days later, mice were administered 100 μl of nephrotoxic serum (IP, ProbeTex), as previously described.[8] At 24 hours post-nephrotoxic serum treatment, all mice were administered either BT503 (IP, 1 mg/kg) or DMSO vehicle (IP, 50% normal saline, 35% PEG300, 5% Tween80, 10% DMSO) daily for 14 days. Urine was collected and mice were euthanized at day 7 and day 14 post-nephrotoxic serum treatment **(Figure 3F)**.

In the HIV-1 transgenic model, hemizygous *Tg26* and wildtype *FVB/n* 8-week-old mice were treated with either BT503 (IP, 1 mg/kg) or DMSO vehicle (IP, 50% normal saline, 35% PEG300, 5% Tween80, 10% DMSO) daily. Urine was collected prior to start of treatment at 8 week of age and, subsequently, at 9 weeks and 10 weeks of age. Serum collection and mouse euthanization occurred at 2 weeks after initiation of BT503 treatment (10 weeks of age) **(Figure 4A)**.

Measurement of urine albumin and creatinine

Urine albumin was quantified by ELISA using a kit from Bethyl Laboratory Inc. Urine creatinine levels were measured in the same samples using the Creatinine Colorimetric Assay Kit (500701; Cayman Chemical) as per manufacturer's protocol.

Isolation of glomeruli from mice for RNA extraction

Mouse glomeruli were isolated as previously described.[3,9] Briefly, mice were perfused with PBS containing 2.5 mg/ml iron oxide and 1% BSA. At the end of perfusion, kidneys were removed, decapsulated, minced into 1 mm^3 pieces, and digested in PBS containing 1 mg/ml collagenase A. Digested tissue was subsequently passed through a 100 μm cell strainer and collected by centrifugation. The pellet was resuspended in 1 ml PBS and glomeruli were collected using a magnet. The purity of glomeruli was verified under microscopy. Total RNA was isolated from kidney glomeruli of mice using the RNeasy Kit (Qiagen, Germantown, MD).

Real-Time PCR

Total RNA from cells was extracted by using TRIzol (Life Technologies). First-strand cDNA was prepared from total RNA (up to 1.25 μg) using the SuperScript™ IV VILO™ Master Mix (Life Technologies), and cDNA was amplified in triplicate using PowerUp™ SYBR™ Green Master Mix on an ABI QuantStudio3 (Applied Biosystems). Primers for human and mouse genes were designed using NCBI Primer-BLAST and validated for efficiency before application **(Supplemental Table 1).** Data were normalized to housekeeping genes (*GAPDH* or *ACTB*) and presented as a fold increase compared with RNA isolated from the control group using the ΔΔCT method.

Western Blot

Differentiated human podocytes were lysed with a fractionation buffer containing 1X protease and phosphatase inhibitor cocktail (Thermo Fisher Scientific) and separate cytoplasmic and nuclear fractions using a modified procedure described before.[10] Cell fractionation lysates from cultured cells were subjected to immunoblot analysis using the antibody for the target protein of interest with mouse anti-GAPDH (Millipore, MAB374) or mouse β-actin (Sigma-Aldrich, A1978) for loading control. Antibodies utilized for target proteins of interest: rabbit anti-KLF15 (Millipore, ABC471), rabbit anti-p50 (Cell Signaling Tech, 13586S), rabbit anti-p65 (Cell Signaling Tech, 8242S), rabbit anti-IκBα (Cell Signaling Tech, 4812S), rabbit anti-IKKα (Cell Signaling Tech, 61294S), rabbit anti-IKKβ (Cell Signaling Tech, 8943S), and rabbit anti-Histone H3 (Cell Signaling Tech, 4620S) antibodies.

Immunofluorescence staining and quantification

Specimens were initially baked for 60 minutes in a 60°C oven and then processed as previously described.[2] Briefly formalin-fixed and paraffin embedded sections were deparaffinized, and endogenous peroxidase was inactivated with H_2O_2. All kidney sections from these mice were prepared in identical fashion. Immunofluorescence was performed using rabbit anti-KLF15 (Genscript), mouse anti-WT1 (Santa Cruz, SC-7385), rabbit anti-Synaptopodin (Sigma, SAB3500585), rabbit anti-p65 (Cell Signaling Tech, 8242S) antibodies. After washing, sections were incubated with the appropriate fluorophore-linked secondary antibody (Alexa Fluor 647 Donkey anti-mouse, Alexa Fluor 568 Donkey anti-rabbit antibodies, Life Technologies). After counter staining with Hoechst (Thermo Fisher Scientific), slides were mounted in Prolong Gold mounting media (Thermo Fisher Scientific) and photographed under a Nikon Eclipse Ni-E Fully Motorized Microscope System with a DS-Qi2 digital camera.

Quantification of KLF15 staining in the podocytes was determined by quantifying the intensity of KLF15 staining (optical density) in $WT1^+Hoechst^+$ staining using ImageJ 1.53c software (National Institute of Health, http://imagej.nih.gov/ij). WT1 staining was quantified by counting the number of $WT1^+$ cells per glomerulus area (μm^2). Quantification of Synaptopodin staining was performed by measuring % glomerular area staining using ImageJ.[2]

Light microscopy and histopathological scoring

Mice were perfused with PBS, and the kidneys were fixed in 10% phosphate buffered formalin overnight and switched to 70% ethanol before processing for histology. Kidney tissue was embedded in paraffin by Stony Brook Medicine Research Histology Core Laboratory (RHCL) - Department of Pathology and 4-μm-thick sections were stained with periodic acid–Schiff (PAS), hematoxylin & eosin (H&E), and Masson’s trichrome (Sigma-Aldrich). Quantification of % FSGS was determined by the renal pathologist, M.P.R. (University of Utah), in a blinded fashion (Huntsman Cancer Institute-University of Utah Health).

Transmission electron microscopy

Mice were perfused with PBS and then immediately fixed in 2.5% glutaraldehyde for electron microscopy as previously described.[2] Transmission Electron Microscopy (TEM) was done in the Central Microscopy Imaging Center at Stony Brook Medicine. Briefly, after embedding the kidney tissues in epoxy resin, ultrathin sections

were stained with uranyl acetate and lead citrate, then mounted on a copper grid, and photographed under a FEI BioTwin G2 Transmission Electron Microscope with AMT XR-60 CCD digital Camera (FEI). Podocyte effacement was quantified as previously described.[2]

Generation of *IKBKB* gatekeeper mutant

The human *IKBKB* plasmid with pCMV6-entry backbone was obtained from Origene (RC219154). The QuikChange II XL Site-Directed Mutagenesis Kit (Agilent) was utilized to generate the $IKBKB^{M96V}$ (methionine to valine) mutant, according to previously described methods.[11] The QuikChange primer design program was employed to design mutagenesis primers. Primers were synthesized by Integrated DNA Technologies. All constructs were sequenced to confirm successful mutagenesis. The $IKBKB^{WT}$ (WT) and $IKBKB^{M96V}$ mutant plasmids were transfected in HEK293 cells and Sepharose FLAG beads (Cell Signaling, 70569) were used to purify the Flag-tagged $IKBKB^{WT}$ and $IKBKB^{M96V}$ mutant.

IKKβ kinase assay

The assays were performed according to our previously established methodologies.[11,12] For *in vitro* kinase assays, myelin basic protein (MBP, sourced from Active Motif, catalog number 31314) was utilized as the substrate for recombinant $IKBKB^{WT}$ and $IKBKB^{M96V}$ mutant. MBP serves as a versatile substrate for various kinases, encompassing both serine/threonine and tyrosine kinases, owing to its multiple phosphorylation sites, rendering it conducive for in vitro kinase assessments.[12] Recombinant IKKβ and MBP were incubated in kinase buffer (Cell Signaling, 9802), supplemented with or without ATP (1 µM), for a duration of 30 minutes at 30°C, followed by kinase assays employing the ADP-Glo Kinase Assay kit (Promega). To assess if the inhibition of IKKβ kinase activity is ATP dependent, the assay was also conducted under ATP concentrations of 1, 5, and 50 µM.

Thermal shift assay

The cells were treated with BT503 (1 µM) for 1 hour, resuspended in PBS, and a 100 µl (~200k cells) was aliquoted. Each tube was treated at a range of temperatures (37°C to 52°C) for 3 minutes using Eppendorf

Mastercycler gradient temperature setting. Protein was subsequently extracted from cells using freeze-thaw cycle, and IKKβ and β-actin were detected using western blot.

RNA sequencing and enrichment analysis

RNA sequencing data was processed as previously described.[13] Briefly, sequencing reads were first aligned to the human genome (version hg38) using Spliced Transcripts Alignment to a Reference (STAR 2.4.1c).[14] Aligned reads were then quantified to the transcriptome (UCSC hg38 annotation) at the gene level using featureCounts (v1.4.6).[15] Read counts were normalized to count per million (CPM), and differentially expressed genes were identified using BioJupies.[16] Enrichment analyses of the differentially expressed genes were performed with *Enrichr*[17,18] using *WikiPathway 2023 Human*[19] and *KEGG 2021 Human*[20] databases using a cutoff of $p < 0.05$ with the Benjamini–Hochberg correction. Normalized read counts were used to create matrix visualized heat maps on *Morpheus* by Broad Institute (https://software.broadinstitute.org/morpheus) with hierarchical clustering, sorted by one minus Pearson's correlation and clustered by rows. Gene set enrichment analysis (GSEA) by Broad Institute and UC San Diego[21] was conducted using raw RNA-seq counts and used to enrich the gene list for the NF-κB signaling pathway to evaluate differences in gene expression level between different treatment groups. Integrated pathway analysis was conducted using *ClusterProfiler*[22] based on differentially expressed genes between treatment groups and sorted based on gene count and adjusted p-value to identify enriched pathways. Using the TRANSFAC software[23,24] we also scanned the promoters of all human genes in the region from (-2000) to the transcription start site with the KLF15 position weight matrix provided by the TRANSFAC system. Enrichment analysis was performed using *Enrichr* and the Fisher's Exact test was used to determine the terms that were overrepresented among the genes with KLF15 binding sites[2,17].

In silico p50/p65 motif enrichment

The following R libraries were used to construct the *in silico* ENCODE ChIP-seq binding of p50 and p65 at the *KLF15* promoter-proximal region within the first intron: *GenomicRanges, rtracklayer, GenomicFeatures, Gviz, biomaRt, BSgenome.Hsapiens.UCSC.hg38*. Gviz was the principal package used to graphically represent the binding of p50 and p65, followed by generation of the ENSEMBL human genome reference (hg38) track which underlies the intronic *KLF15* region-of-interest plotting.

Computational Methods:

Protein target selection and modeling

Coordinates for docking ligands to IKKβ were obtained from the Protein Data Bank (pdb code 4KIK).[25] The structure is an asymmetric dimer and both ATP binding sites contained the Staurosporine analog labeled K252a. Chain B of the dimer was a more complete structure and thus retained for modeling[25]. The program Chimera[26], with the Modeller interface[27], was employed to fill in missing residues (180-183) on the activation loop (4KIK numbering) with no movement of flanking residues allowed. To simplify docking setup of the kinase domain, residues numbered greater than 316 and crystallographic waters were deleted. These deletions removed the majority of the central ubiquitin like domain and the entirety of the C terminal dimerization domain. A homologous protein-ligand structure was downloaded from the pdb (code 1GIH)[28], comprised of a CDK2 construct, in which the ATP binding site was modified to mimic CDK4 and bound to the inhibitor named 1PU (1-[(9bR)-5-oxo-1,2,3,9b-tetrahydrobenzo[f]pyrrolizin-9-yl]-3-pyridin-2-yl-urea). The binding site in the CDK4 mimic from 1GIH shares high structural homology and reasonable sequence homology with IKKβ from 4KIK and the inhibitor 1PU share similarity with the inhibitors being investigated here, thus, the 1GIH structure provides a means to generate a second inhibitor-bound IKKβ complex (see results). To generate this second complex, as reference for docking, 1GIH was matched to 4KIK (backbone atoms) using the Chimera command "matchmaker". As expected, the procedure yielded a well overlapped structure with low RMSD (RMSD = 0.872 Å over 140 pruned atom pairs). The sequence homology for the group of residues closest to the binding site, defined here as ca. 8 Å from 1PU (N=53) was 37% by identity and 65% by similarity.[29] The homology for the entire catalytic domains, defined here as residues 12 to 311 (4KIK numbering), was 25% identity and 39% similarity (calculations from EMBOSS Needle, www.ebi.ac.uk/Tools/psa/emboss_needle).[29]

Protein target and ligand setups details

Protocols for preparing ligands and proteins for docking have previously been described.[30,31] Briefly, ligand K252a (code 4KIK) was separated from IKKβ, protonated, and assigned partial atomic charges (AM1BCC method)[32] using the program Chimera.[26] The utility programs tleap and antechamber,[33] from the AMBER[34] suite of programs, were employed to re-assemble the complex and assign force field parameters (FF14SB[35] for

protein, GAFF[36] for ligand). To relax the structure prior to docking, the K252a/IKKβ complex was subsequently minimized for 1000 steps using the AMBER[34] module sander as part of our standard DOCK6 FLX[31] preparation protocol. Other ligands employed for docking in this work, including 1PU (code 1GIH)[28], INH14[37], and BT503, were prepared in a similar manner using Chimera and saved as MOL2 files.

DOCK Setups for the Binding Site

The energy-minimized protein was separated from the K252a/IKKβ complex, saved as a separate MOL2 file, and used to prepare files needed by DOCK6[30] for docking. Briefly, a protein molecular surface (unprotonated protein) was computed using the Chimera tool DMS, and then the DOCK utility sphgen[38] was used to generate docking spheres on the molecular surface.[39] Spheres within ca. 8 Å of the cognate ligand were saved to facilitate ligand anchor orientation in the binding site.[39] Docking grids (grid program)[40], which help speed up the calculations, employed a 6-9 Lennard Jones potential for the van der Waals (VDW) term and a 4r distance dependent dielectric Coulombic potential for the electrostatic (ES) term. Grid dimensions were based on a bounding box of 8 Å from any docking sphere and employed a spacing resolution of 0.3 Å. Following these preparation steps, a DOCK energy minimization was performed for the theoretical complex of 1PU with IKKβ (from the 1GIH to 4KIK alignment) which served as a reference for the study. To facilitate molecular footprint calculations (discussed below) the DOCK multigrid[41] protocol was used to isolate key residues involved in protein ligand binding.

Docking protocol - Grid score only

To complement the energy minimizations, pose reproduction calculations were also performed (FLX protocol) for K252a with 4KIK and 1PU with 1GIH. We also docked 1PU into IKKβ using the minimized aligned structure as the "theoretical reference." Pose reproduction experiments employed equal weighting of VDW and ES terms, with each experiment generating 100 conformers for each ligand with the top one being retained for geometric comparisons and molecular dynamics (MD) simulations.

Docking Protocol - Grid Score with Restraints

For INH14 and BT503 docking, a similarity-based function comprised of Hungarian Matching Similarity (HMS)[42] and Volume Overlap Similarity (VOS)[43] terms was employed (along with grid score) to help bias sampling in IKKβ towards the conformation adopted by the aligned 1PU reference which makes key H-bond interactions with the backbone of Cys099 (see Results). The weights employed for VDW, ES, HMS, and VOS

terms were 1, 1, 5, and -5, respectively. The above scoring function was used during orienting, minimization, dihedral sampling, as well as final rank ordering. The VDW and ES terms were assessed to make sure the above weightings did not produce unlikely conformations for the top ranked pose. As before, 100 conformers were saved for each ligand, with the top pose being retained for molecular dynamics simulations. Molecular footprints[41,44] for the docked and reference ligands, based on the most favorable per-residue interaction energy decomposition (VDW and ES terms) observed across the "collective" group of poses in IKKβ were also computed.

Minimization, equilibration, and production (Molecular Dynamics)

Docked and reference poses used for MD simulations were prepared as above using the AMBER[34] utility programs tleap and antechamber[33] to assemble coordinates and assign the required force fields (FF14SB[35] for protein, GAFF[36] for ligand). Each complex was solvated with an octahedron of 12 Å TIP3P[45] waters and neutralized as appropriate with sodium and chloride counter ions. A previously described nine-step protocol[46] was then employed to minimize and equilibrate each complex prior to running production MD.

Briefly, a restrained energy minimization followed by short MD (100 ps) was performed (steps 1-2, 5 kcal/mol - Å^{-2} restraint, heavy atoms only) followed by three minimizations in which the weights were decreased from 2 to 0.1 to 0.05 kcal/mol - Å^{-2} (steps 3-5, heavy atoms only). Three additional short MD equilibrations (100 ps each) were performed in which the weights were decreased from 1 to 0.5 to 0.1 kcal/mol - Å^{-2} (step 6-8, heavy atoms only). A final step of MD equilibration was performed with a restraint of 0.1 kcal/mol - Å^{-2} applied only to the protein backbone (step 9). Production MD employed a 1 kcal/mol - Å^{-2} backbone only restraint. For each system, GPU accelerated MD[47] was performed for 10 nanoseconds. Chimera was used to visually assess simulation outcomes and the 3D coordinate analysis program cpptraj[48] was used to calculate ligand RMSDs relative to originally docked poses. Cpptraj was also used to extract evenly spaced snapshots (frames) from the production MD trajectories, and the ambpdb and antechamber[33,34] utilities were used to convert snapshots to MOL2 format (Sybyl atom types), so that time averaged footprints could be estimated using DOCK6[30]. Energy minimizations of each frame (ligand restraint weight = 10 kcal/mol - Å^{-2}) were performed to account for any potential differences in force field parameters in switching from DOCK to AMBER format. Time averaged footprints computed using DOCK6 employed the same residue list as determined from the original docking calculations but were performed in Cartesian space (6-12 LJ potential, 4r distance dependent dielectric).

SUPPLEMENTAL REFERENCES

1. Saleem MA, O'Hare MJ, Reiser J, et al. A conditionally immortalized human podocyte cell line demonstrating nephrin and podocin expression. *J Am Soc Nephrol*. Mar 2002;13(3):630-638.
2. Mallipattu SK, Guo Y, Revelo MP, et al. Krüppel-Like Factor 15 mediates glucocorticoid-induced restoration of podocyte differentiation markers. *J Am Soc Nephrol*. Jan 2017;28(1):166-184. doi:10.1681/ASN.2015060672
3. Mallipattu SK, Liu R, Zheng F, et al. Krüppel-like factor 15 (KLF15) is a key regulator of podocyte differentiation. *J Biol Chem*. Jun 2012;287(23):19122-19135. doi:10.1074/jbc.M112.345983
4. Lee HW, Khan SQ, Faridi MH, et al. A podocyte-based automated screening assay identifies protective small molecules. *J Am Soc Nephrol*. Nov 2015;26(11):2741-2752. doi:10.1681/ASN.2014090859
5. Bialkowska AB, Du Y, Fu H, Yang VW. Identification of novel small-molecule compounds that inhibit the proproliferative Krüppel-like factor 5 in colorectal cancer cells by high-throughput screening. *Mol Cancer Ther*. Mar 2009;8(3):563-570. doi:10.1158/1535-7163.MCT-08-0767
6. Asada M, Rauch A, Shimizu H, et al. DNA binding-dependent glucocorticoid receptor activity promotes adipogenesis via Krüppel-like factor 15 gene expression. *Lab Invest*. Feb 2011;91(2):203-215. doi:10.1038/labinvest.2010.170
7. Ahn JM, Boyle NA, MacDonald MT, Janda KD. Peptidomimetics and peptide backbone modifications. *Mini Rev Med Chem*. Oct 2002;2(5):463-473. doi:10.2174/1389557023405828
8. Estrada CC, Paladugu P, Guo Y, et al. Krüppel-like factor 4 is a negative regulator of STAT3-induced glomerular epithelial cell proliferation. *JCI Insight*. Jun 2018;3(12)doi:10.1172/jci.insight.98214
9. Mallipattu SK, He JC. The podocyte as a direct target for treatment of glomerular disease? *Am J Physiol Renal Physiol*. Jul 2016;311(1):F46-51. doi:10.1152/ajprenal.00184.2016
10. Suzuki K, Bose P, Leong-Quong RY, Fujita DJ, Riabowol K. REAP: A two minute cell fractionation method. *BMC Res Notes*. Nov 2010;3:294. doi:10.1186/1756-0500-3-294
11. Kim JY, Bai Y, Jayne LA, et al. A kinome-wide screen identifies a CDKL5-SOX9 regulatory axis in epithelial cell death and kidney injury. *Nat Commun*. Apr 21 2020;11(1):1924. doi:10.1038/s41467-020-15638-6
12. Castano A, Silvestre M, Wells CI, et al. Discovery and characterization of a specific inhibitor of serine-threonine kinase cyclin-dependent kinase-like 5 (CDKL5) demonstrates role in hippocampal CA1 physiology. *Elife*. Jul 25 2023;12doi:10.7554/eLife.88206
13. Wang Z, Ma'ayan A. An open RNA-Seq data analysis pipeline tutorial with an example of reprocessing data from a recent Zika virus study. *F1000Res*. Jul 2016;5:1574. doi:10.12688/f1000research.9110.1
14. Dobin A, Davis CA, Schlesinger F, et al. STAR: ultrafast universal RNA-seq aligner. *Bioinformatics*. Jan 2013;29(1):15-21. doi:10.1093/bioinformatics/bts635
15. Liao Y, Smyth GK, Shi W. featureCounts: an efficient general purpose program for assigning sequence reads to genomic features. *Bioinformatics*. Apr 2014;30(7):923-930. doi:10.1093/bioinformatics/btt656
16. Lachmann A, Torre D, Keenan AB, et al. Massive mining of publicly available RNA-seq data from human and mouse. *Nat Commun*. Apr 2018;9(1):1366. doi:10.1038/s41467-018-03751-6
17. Chen EY, Tan CM, Kou Y, et al. Enrichr: interactive and collaborative HTML5 gene list enrichment analysis tool. *BMC Bioinformatics*. Apr 2013;14:128. doi:10.1186/1471-2105-14-128
18. Kuleshov MV, Jones MR, Rouillard AD, et al. Enrichr: a comprehensive gene set enrichment analysis web server 2016 update. *Nucleic Acids Res*. Jul 2016;44(W1):W90-97. doi:10.1093/nar/gkw377
19. Slenter DN, Kutmon M, Hanspers K, et al. WikiPathways: a multifaceted pathway database bridging metabolomics to other omics research. *Nucleic Acids Res*. Jan 2018;46(D1):D661-d667. doi:10.1093/nar/gkx1064
20. Kanehisa M, Goto S. KEGG: kyoto encyclopedia of genes and genomes. *Nucleic Acids Res*. Jan 2000;28(1):27-30.
21. Subramanian A, Tamayo P, Mootha VK, et al. Gene set enrichment analysis: A knowledge-based approach for interpreting genome-wide expression profiles. *P Natl Acad Sci USA*. Oct 2005;102(43):15545-15550. doi:10.1073/pnas.0506580102
22. Yu GC, Wang LG, Han YY, He QY. clusterProfiler: an R package for comparing biological themes among gene clusters. *Omics*. May 2012;16(5):284-287. doi:10.1089/omi.2011.0118
23. Matys V, Fricke E, Geffers R, et al. TRANSFAC: transcriptional regulation, from patterns to profiles. Research Support, Non-U.S. Gov't. *Nucleic Acids Res*. Jan 2003;31(1):374-378.

24. Matys V, Kel-Margoulis OV, Fricke E, et al. TRANSFAC and its module TRANSCompel: transcriptional gene regulation in eukaryotes. *Nucleic Acids Res*. Jan 2006;34(Database issue):D108-110. doi:10.1093/nar/gkj143
25. Liu S, Misquitta YR, Olland A, et al. Crystal structure of a human IκB kinase β asymmetric dimer. *J Biol Chem*. Aug 2013;288(31):22758-22767. doi:10.1074/jbc.M113.482596
26. Pettersen EF, Goddard TD, Huang CC, et al. UCSF Chimera--a visualization system for exploratory research and analysis. *J Comput Chem*. Oct 2004;25(13):1605-1612. doi:10.1002/jcc.20084
27. Sali A, Blundell TL. Comparative protein modelling by satisfaction of spatial restraints. *J Mol Biol*. Dec 1993;234(3):779-815. doi:10.1006/jmbi.1993.1626
28. Ikuta M, Kamata K, Fukasawa K, et al. Crystallographic approach to identification of cyclin-dependent kinase 4 (CDK4)-specific inhibitors by using CDK4 mimic CDK2 protein. *J Biol Chem*. Jul 2001;276(29):27548-27554. doi:10.1074/jbc.M102060200
29. Madeira F, Pearce M, Tivey ARN, et al. Search and sequence analysis tools services from EMBL-EBI in 2022. *Nucleic Acids Res*. Jul 2022;50(W1):W276-W279. doi:10.1093/nar/gkac240
30. Allen WJ, Balius TE, Mukherjee S, et al. DOCK 6: Impact of new features and current docking performance. *J Comput Chem*. Jun 2015;36(15):1132-1156. doi:10.1002/jcc.23905
31. Mukherjee S, Balius TE, Rizzo RC. Docking validation resources: protein family and ligand flexibility experiments. *J Chem Inf Model*. Nov 2010;50(11):1986-2000. doi:10.1021/ci1001982
32. Jakalian A, Jack DB, Bayly CI. Fast, efficient generation of high-quality atomic charges. AM1-BCC model: II. Parameterization and validation. *J Comput Chem*. Dec 2002;23(16):1623-1641. doi:10.1002/jcc.10128
33. Wang J, Wang W, Kollman PA, Case DA. Automatic atom type and bond type perception in molecular mechanical calculations. *J Mol Graph Model*. Oct 2006;25(2):247-260. doi:10.1016/j.jmgm.2005.12.005
34. D.A. Case HMA, K. Belfon, I.Y. Ben-Shalom, J.T. Berryman, S.R. Brozell, D.S. Cerutti, T.E. Cheatham, III, G.A. Cisneros, V.W.D. Cruzeiro, T.A. Darden, N. Forouzesh, G. Giambaşu, T. Giese, M.K. Gilson, H. Gohlke, A.W. Goetz, J. Harris, S. Izadi, S.A. Izmailov, K. Kasavajhala, M.C. Kaymak, E. King, A. Kovalenko, T. Kurtzman, T.S. Lee, P. Li, C. Lin, J. Liu, T. Luchko, R. Luo, M. Machado, V. Man, M. Manathunga, K.M. Merz, Y. Miao, O. Mikhailovskii, G. Monard, H. Nguyen, K.A. O'Hearn, A. Onufriev, F. Pan, S. Pantano, R. Qi, A. Rahnamoun, D.R. Roe, A. Roitberg, C. Sagui, S. Schott-Verdugo, A. Shajan, J. Shen, C.L. Simmerling, N.R. Skrynnikov, J. Smith, J. Swails, R.C. Walker, J. Wang, J. Wang, H. Wei, X. Wu, Y. Wu, Y. Xiong, Y. Xue, D.M. York, S. Zhao, Q. Zhu, and P.A. Kollman. Amber 2023 University of California, San Francisco. 2023;
35. Maier JA, Martinez C, Kasavajhala K, Wickstrom L, Hauser KE, Simmerling C. ff14SB: Improving the accuracy of protein side chain and backbone parameters from ff99SB. *J Chem Theory Comput*. Aug 2015;11(8):3696-3713. doi:10.1021/acs.jctc.5b00255
36. Wang J, Wolf RM, Caldwell JW, Kollman PA, Case DA. Development and testing of a general amber force field. *J Comput Chem*. Jul 2004;25(9):1157-1174. doi:10.1002/jcc.20035
37. Drexel M, Kirchmair J, Santos-Sierra S. INH14, a small-molecule urea derivative, inhibits the IKKα/β-dependent TLR inflammatory response. *Chembiochem*. Mar 2019;20(5):710-717. doi:10.1002/cbic.201800647
38. DesJarlais RL, Sheridan RP, Seibel GL, Dixon JS, Kuntz ID, Venkataraghavan R. Using shape complementarity as an initial screen in designing ligands for a receptor binding site of known three-dimensional structure. *J Med Chem*. Apr 1988;31(4):722-729. doi:10.1021/jm00399a006
39. Kuntz ID, Blaney JM, Oatley SJ, Langridge R, Ferrin TE. A geometric approach to macromolecule-ligand interactions. *J Mol Biol*. Oct 1982;161(2):269-288. doi:10.1016/0022-2836(82)90153-x
40. Shoichet BK, Bodian DL, Kuntz ID. Molecular docking using shape descriptors. *J Comput Chem*. Apr 1992;13(3):380-397. doi:DOI 10.1002/jcc.540130311
41. Balius TE, Allen WJ, Mukherjee S, Rizzo RC. Grid-based molecular footprint comparison method for docking and de novo design: application to HIVgp41. *J Comput Chem*. May 2013;34(14):1226-1240. doi:10.1002/jcc.23245
42. Allen WJ, Rizzo RC. Implementation of the Hungarian algorithm to account for ligand symmetry and similarity in structure-based design. *J Chem Inf Model*. Feb 2014;54(2):518-529. doi:10.1021/ci400534h
43. Sastry GM, Dixon SL, Sherman W. Rapid shape-based ligand alignment and virtual screening method based on atom/feature-pair similarities and volume overlap scoring. *J Chem Inf Model*. Oct 2011;51(10):2455-2466. doi:10.1021/ci2002704
44. Balius TE, Mukherjee S, Rizzo RC. Implementation and evaluation of a docking-rescoring method using molecular footprint comparisons. *J Comput Chem*. Jul 2011;32(10):2273-2289. doi:10.1002/jcc.21814

45. Jorgensen WL, Chandrasekhar J, Madura JD, Impey RW, Klein ML. Comparison of simple potential functions for simulating liquid water. *J Chem Phys*. Jul 1983;79(2):926-935. doi:Doi 10.1063/1.445869
46. Zhou YC, Elmes MW, Sweeney JM, et al. Identification of fatty acid binding protein 5 inhibitors through similarity-based screening. *Biochemistry-Us*. Oct 2019;58(42):4304-4316. doi:10.1021/acs.biochem.9b00625
47. Salomon-Ferrer R, Gotz AW, Poole D, Le Grand S, Walker RC. Routine microsecond molecular dynamics simulations with AMBER on GPUs. 2. explicit solvent particle mesh ewald. *J Chem Theory Comput*. Sep 2013;9(9):3878-3888. doi:10.1021/ct400314y
48. Roe DR, Cheatham TE. PTRAJ and CPPTRAJ: Software for processing and analysis of molecular dynamics trajectory data. *J Chem Theory Comput*. Jul 2013;9(7):3084-3095. doi:10.1021/ct400341p

SUPPLEMENTAL FIGURE LEGENDS

Supplemental Figure 1: Structure-activity relationship study of C-7 by modifying different core structural moieties and synthetic scheme. (A) Structure-activity relationship analysis was conducted on C-7 with 3 structural moieties (Part A contains 4-thiomethyl group of phenyl ring, Part B contains substituted urea linker and Part C contains pyridine ring) to generate 14 novel analogues. **(B)** Synthetic scheme for amide analogue, BT501. **(C)** General synthetic scheme for C-7 analogues (BT502 and BT503) for substituting the pyridine ring. **(D)** General procedure for synthesis to replace the urea derivatives (in ring Part B) with oxadiazole (BT504, BT509, BT510, BT514). **(E)** Synthetic scheme for BT513. **(F)** Synthetic scheme for BT506, BT507 and BT508. **(G)** General procedure for synthesis of amide analogues (BT505, BT511).

Supplemental Figure 2: BT503 increased KLF15 activity in differentiated human podocytes. (A) Fold change in KLF15 reporter activity in human podocytes under non-permissive (differentiated) conditions (37°C) as compared to permissive conditions (33°C) (relative to DMSO, normalized to Renilla) (n=6, ***p<0.001, Kruskal-Wallis test with Dunn's post-test). **(B)** Heatmap analysis of all 600 DEGs in DMSO-, BT503-treated mice in 37°C vs. 33°C. Heatmap analysis of WikiPathway and KEGG Pathway for **(C)** upregulated and **(D)** downregulated DEGs (with enrichment for pathways involving genes with KLF15 BS). **(E)** Heatmap of top transcription factors from ChIP-enrichment analysis (ChEA) of DEGs. **(F)** Heatmap analysis of DEGs encompassing NF-κB pathway. **(G-I)** Western blot with quantification for IKKβ, IKKα, and GAPDH. Representative blots from four different experiments are shown. (n=4, ***p<0.001 compared to all other groups, Kruskal-Wallis test with Dunn's post-test).

Supplemental Figure 3: Molecular footprints for energy-minimized 1PU, docked 1PU, and INH14 on IKKβ. (A-B) Molecular footprints (per residue energy breakdown) for energy-minimized 1PU (purple), docked 1PU (light blue), and INH14 (orange) on IKKβ. Only three protein residues are shown for clarity. Potential H-bonds (magenta). DOCK scores reported in kcal/mol. **(C)** Ligand root-mean square deviations (RMSD) from simulations in IKKβ relative to their original docked (1PU, INH14, BT503) or cognate ligand pose (K252a x-ray pose) as a function of simulation time (box car averaged over 200 frames, 2000 frames total). **(D)** Time averaged molecular footprints (N=100 frames, 10 ns) for 1PU (cyan) and BT503 (green) with error bars representing standard

deviations. The VDW (top) and ES (bottom) residue lists correspond to the top 20 most favorable residues (plus remainder residues labeled: Remain). Energies in kcal/mol.

Supplemental Figure 1

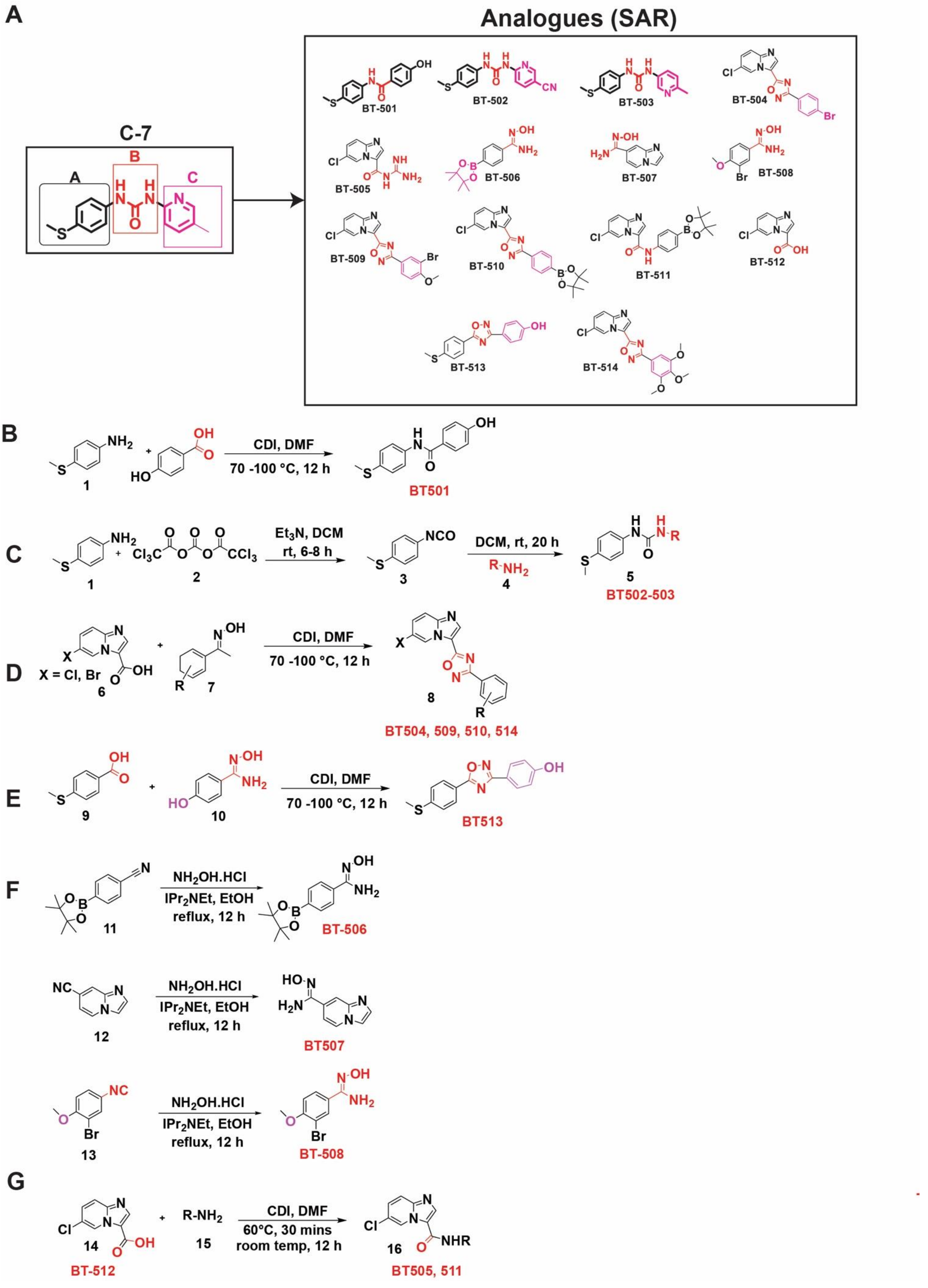

Supplemental Figure 2

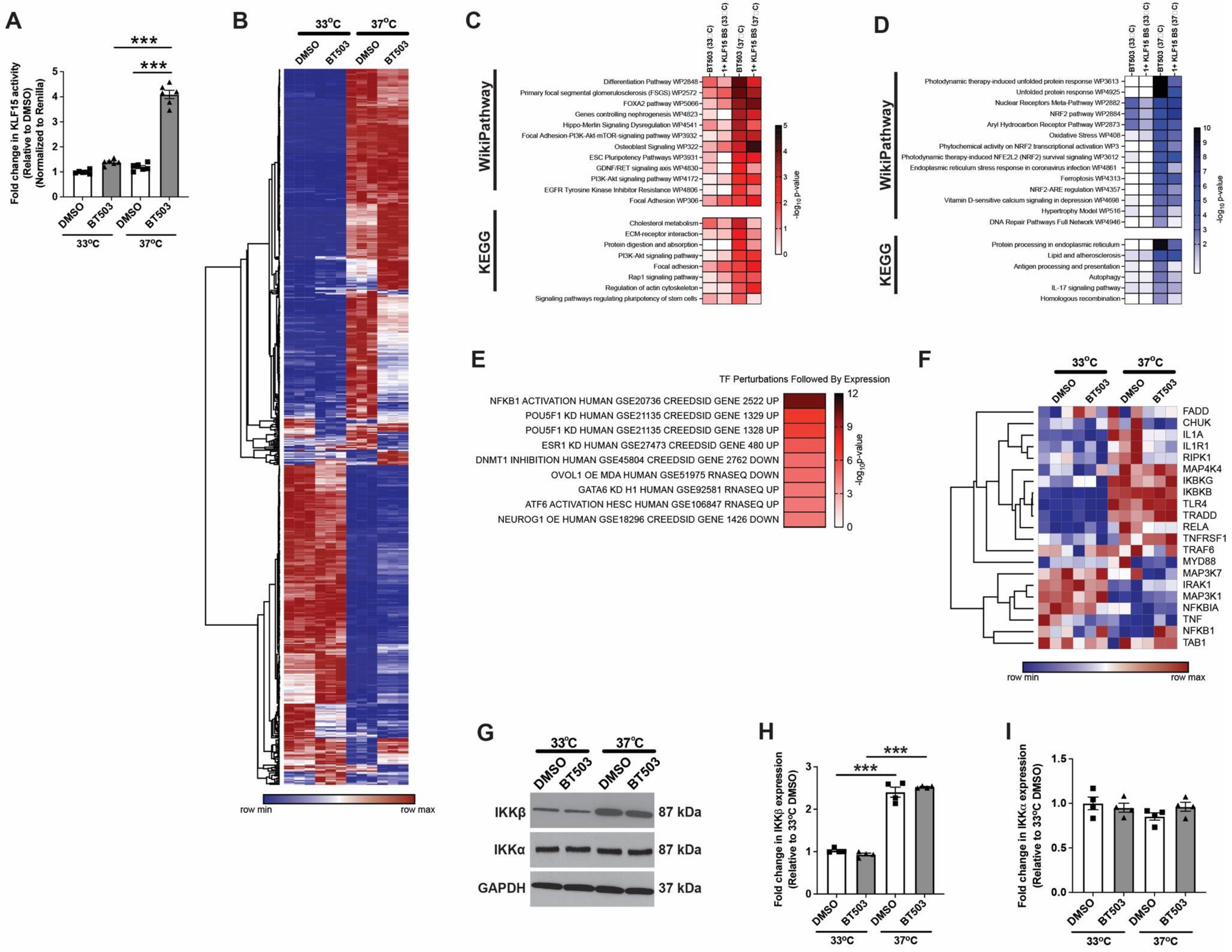

Supplemental Figure 3

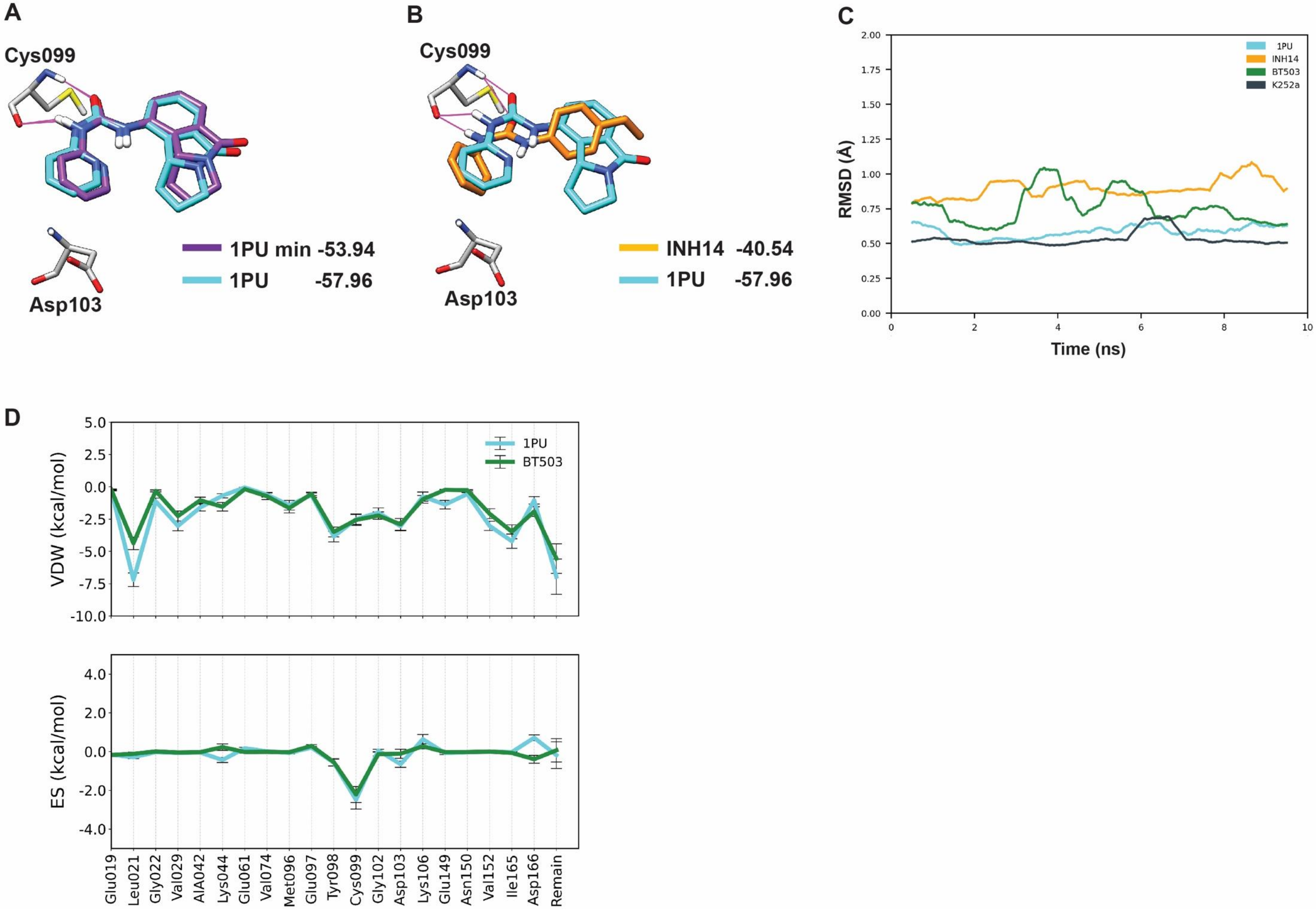

Supplemental Table 1: Primers for real-time PCR

Gene Name	Species	Forward primer	Reverse primer
KLF15	*Human*	GTTGGGTATCTGGGTGATAGGC	TGAGAGTCGGGACTGGAACAG
Synpo	*Human*	AGCCCAAGGTGACCCCGAAT	CCCTGTCACGAGGTGCTGGC
WT1	*Human*	CAGGCTGCAATAAGAGATATTTTA	GAAGTCACACTGGTATGGTTTCT
GAPDH	*Human*	TGTTGCCATCAATGACCCCTT	CTCCACGACGTACTCAGCG
ACTB	*Human*	AGAGCTACGAGCTGCCTGAC	AGCACTGTGTTGGCGTACAG
Klf15	*Mouse*	AGAGCAGCCACCTCAAGGCCCA	TCACACCCGAGTGAGATCGCCGGT
Wt1	*Mouse*	GAGAGCCAGCCTACCATCC	GGGTCCTCGTGTTTGAAGGAA
Col1a1	*Mouse*	CCAGCCGCAAAGAGTCTACA	GTTTCCACGTCTCACCATTG
Fn1	*Mouse*	ATGGTACAGCTGATCCTGCC	GCCCTGGTTTGTACCTGCTA
Acta2	*Mouse*	CAGCGGGCATCCACGAA	GCCACCGATCCAGACAGA
Vimentin	*Mouse*	GGATCAGCTCACCAACGACA	GGTCAAGACGTGCCAGAGAA
Gapdh	*Mouse*	GCCATCAACGACCCCTTCAT	ATGATGACCCGTTTGGCTCC
Actb	*Mouse*	GTTCCGATGCCCTGAGGCTCTT	CGTCACACTTCATGATGGAATTGA

Supplemental Table 2: Top hits from the KLF15 High-Throughput Screen in Human Podocytes

Hits	NSC	Pubchem	Description	Fold Change	EC50 (nM)
C-1	33004	5351155	Phenol,4-(2-benzothiazolyl)	3.0083	99.63
C-2	320846	71750	Batracyclin	3.0717	14.89
C-3	80087	5784745	[(4-Dimethylamino)benzylidene]indene	2.6455	17.73
C-4	667251	5351425	2-Propenenitrile, 3-[3-(dimethylamino)phenyl]-2-phenyl-	2.5670	23.74
C-5	159031	292850	5-methoxy-2-phenyl-1H-indole	3.1354	55.88
C-6	20619	3003755	1,3-bis(3-methylpyridin-2-yl)thiourea	2.7113	770.50
**C-7**	**142269**	**285402**	**1-(4-Methylpyridin-2-yl)-3-(4-methylsulfanylphenyl)urea**	**3.1695**	**11.90**
C-8	214029	310541	3-fluoro-N-(2-naphthyl)benzamide	2.7914	70.53
**C-9**	**158549**	**292642**	**2-benzo[e]benzotriazol-2-ylaniline**	**2.7182**	**76.26**
C-10	305743	65758	Pifexole	2.7219	332.40
C-11	343557	335175	2-amino-N-(2,4-dichlorophenyl)benzamide	2.6436	705.90
C-12	33738	234249	2-[2-(3,4-Dichloroanilino)-2-oxoethoxy]benzamide	3.1347	37.28
C-13	522131	351549	N-(3,4-dichlorophenyl)-N'-(2-hydroxyphenyl)urea	2.6384	86.12
C-14	164435	295274	1-(4-Anilinophenyl)-3-(2-chlorophenyl)urea	3.0342	42.10
**C-15**	**158959**	**292795**	**2,4-dichloro-N-(naphthalen-2-yl)benzamide**	**2.9715**	**10.93**
C-16	177407	5383615	5,6-dichloro-2-[3-(trifluoromethyl)phenyl]-1H-imidazo[4,5-b]pyrazine	2.8171	52.14
C-17	353527	8137375	Tin(IV), chlorotriphenyl[1-(4-ethoxyphenyl)- 3-cyanoureato]-, hydrogen, triethylamine	4.6159	NA
C-18	693632	5351439	2-(2-(1-(pyrimidin-4-yl)ethylidene)hydrazinyl)benzo[d]thiazole	3.2863	156.50
C-19	70931	122724	Celastrol	3.2798	11.18
C-20	78130	105268	3-[(4-Methoxyphenyl)azo]-2,6-pyridinediamine	3.1426	84.44
C-21	82025	255922	2-hydroxy-N-(4-methyl-2-nitrophenyl)-3-nitrobenzamide	3.1125	61.35
C-22	757441	6450551	Axitinib	3.0642	~286693
C-23	184403	5351258	5-Methylbenzo[c]phenanthridin-5-ium-2,3,8,9-tetrol;pyridine;chloride;hydrochloride	3.0541	296.00
C-24	21683	228624	(4Z)-5-imino-4-[(4-methylphenyl)hydrazinylidene]-1-phenylpyrazol-3-amine	3.0292	2670.00
C-25	680516	387118	N,N,3-trimethyl-4-[(E)-6-quinolylazo]aniline	3.0228	15.77
C-26	658350	135462447	2-[2-[(2E)-2-[(5Z)-5-(anilinomethylene)-3-ethyl-4-oxo-thiazolidin-2-ylidene]hydrazino]thiazol-5-yl]-N-(o-tolyl)acetamide	3.0168	484.20
C-27	640584	5351382	3-(3,4-dichlorophenyl)-1-(2-(3,5-diphenyl-1H-pyrazol-1-yl)-4-methylthiazol-5-yl)prop-2-en-1-one	2.8899	NA
C-28	680515	39164	7-(p-N,N-Dimethylaminophenylazo)benzofuran	2.8704	~6310

C-29	70717	4204363	s-Triazolo[4,8-methyl	2.8700	549.80
C-30	106461	267294	2,5-dipyridin-2-yl-1,3,4-thiadiazole	2.8592	334.80
C-31	634224	366050	1-Indenone, 3-hydroxy-2-(2-quinoxalinyl)-	2.8466	45.86
C-32	658293	5351418	ethyl (2Z)-5-methyl-2-[(4-nitrophenyl)methylidene]-3-oxo-[1,3]thiazolo[2,3-b][1,3]thiazol-4-ium-6-carboxylate;chloride	2.8360	NA
C-33	33005	95746	(4E)-4-(3H-1,3-benzothiazol-2-ylidene)-3-hydroxycyclohexa-2,5-dien-1-one	2.7511	490.10
C-34	635404	366687	2-hydroxy-N'-(2-oxo-1,2-dihydro-3H-indol-3-ylidene)benzohydrazide	2.6900	121.60
C-35	367416	339701	7-Methoxy-1,4,6-benzotriazaphenothiazine	2.6597	319.20
C-36	176367	708470	N-(pyridin-2-ylcarbamothioyl)benzamide	2.6427	291.90
C-37	9358	81528	4-(2-pyridylazo)-n,n-dimethylaniline	2.6148	26.96
C-38	50648	242249	2-<3-Nitro-phenylcarbamoyl>-naphth-1-ol	2.6069	144.80
C-39	289748	324415	N,N-dimethyl-4-[(4-methyl-3-oxido-1,3-thiazol-3-ium-2-yl)diazenyl]aniline	2.5988	~ 6890
C-40	130872	6164010	2-(4-methoxyphenyl)-3-pyridin-2-ylprop-2-enenitrile	2.5975	545.50
C-41	647136	372186	1-Methoxy-7,8-dimethylphenazine	2.5935	214.90
C-42	657598	5351414	Thiazolo[2,3-b]thiazolium, 2,3-dihydro- 2-[(4-nitrophenyl)methylene]- 5-(3-nitrophenyl)-3-oxo-, chloride	2.5844	NA
C-43	400938	135441716	4-[2-(4-methylphenyl)hydrazinyl]benzotriazol-5-one	2.5682	866.00
C-44	164880	5937213	1-[2-(2,3-Dimethoxyphenyl)vinyl]isoquinoline	2.5631	~1839

Supplemental Table 3: Predicted ADME for KLF15 Agonists

KLF15 Agonist	C-7	BT501	BT502	BT503
**Physiochemical Properties**				
MW (g/mol)	273.35	259.32	284.34	273.35
# heavy atoms	19	18	20	19
# aromatic heavy atoms	12	12	12	12
# rotational bonds	5	4	5	5
# H-bond acceptors	2	2	3	2
# H-dond donors	2	2	2	2
**Lipophilicity (XLOGP3) (-0.7 to +5.0)**	2.62	2.78	1.98	2.33
**Polarity (TSPA) (20 to 130 Å²)**	79.32	74.63	103.11	79.32
**Solubility (log S) (not higher than 6)**	-3.32	-3.43	-2.96	-3.14
**Pharmacokinetics**				
GI absorption	High	High	High	High
BBB permeability	No	Yes	No	No
**Druglikeness**				
Lipinski	Yes	Yes	Yes	Yes
Ghose	Yes	Yes	Yes	Yes
Veber	Yes	Yes	Yes	Yes
Egan	Yes	Yes	Yes	Yes
Muegge	Yes	Yes	Yes	Yes
**Bioavailability score**	0.55	0.55	0.55	0.55
**Leadlikeness**	Yes	Yes	Yes	Yes

Supplemental Table 4: Upregulated DEGs (BT503 vs. DMSO (+/- LPS))

Gene Symbol	Description
LDLR	low density lipoprotein receptor
PPME1	protein phosphatase methylesterase 1
SCD	stearoyl-CoA desaturase
SLC22A5	solute carrier family 22 member 5
TINAGL1	tubulointerstitial nephritis antigen like 1
CCN2	cellular communication network factor 2
COL8A1	collagen type VIII alpha 1 chain
COL11A1	collagen type XI alpha 1 chain
FASN	fatty acid synthase
LTBP2	latent transforming growth factor beta binding protein 2
LTBP3	latent transforming growth factor beta binding protein 3
TENM2	teneurin transmembrane protein 2
PLK2	polo like kinase 2
F8A2	coagulation factor VIII associated 2
G0S2	G0/G1 switch 2
ENC1	ectodermal-neural cortex 1
DCDC2	doublecortin domain containing 2
MAMDC2	MAM domain containing 2
SEMA7A	semaphorin 7A (John Milton Hagen blood group)
AHNAK	AHNAK nucleoprotein
OXTR	oxytocin receptor
KRT80	keratin 80
IRS1	insulin receptor substrate 1
ITGA3	integrin subunit alpha 3
CAV1	caveolin 1
GAS6	growth arrest specific 6
FABP5P7	fatty acid binding protein 5 pseudogene 7
FJX1	four-jointed box kinase 1
HAPLN1	hyaluronan and proteoglycan link protein 1
LSS	lanosterol synthase
ADAMTS3	ADAM metallopeptidase with thrombospondin type 1 motif 3
NPR3	natriuretic peptide receptor 3
NPRL3	NPR3 like, GATOR1 complex subunit
AXL	AXL receptor tyrosine kinase
HS3ST3A1	heparan sulfate-glucosamine 3-sulfotransferase 3A1
ANXA3	annexin A3
GFRA1	GDNF family receptor alpha 1
COL7A1	collagen type VII alpha 1 chain
LOXL1	lysyl oxidase like 1
MTCL1	microtubule crosslinking factor 1
EFEMP1	EGF containing fibulin extracellular matrix protein 1

F2R	coagulation factor II thrombin receptor
KRT7	keratin 7
AGRN	agrin
WNT5B	Wnt family member 5B
TGM2	transglutaminase 2
CD70	CD70 molecule
ELFN2	extracellular leucine rich repeat and fibronectin type III domain containing 2
ADAMTSL1	ADAMTS like 1
NIBAN2	niban apoptosis regulator 2
NR2F2	nuclear receptor subfamily 2 group F member 2
NUAK2	NUAK family kinase 2
SCRIB	scribble planar cell polarity protein
SOX9	SRY-box transcription factor 9
ADAMTS1	ADAM metallopeptidase with thrombospondin type 1 motif 1
KAZN	kazrin, periplakin interacting protein
ANKRD33B	ankyrin repeat domain 33B
PDE1C	phosphodiesterase 1C
WNK4	WNK lysine deficient protein kinase 4
MVD	mevalonate diphosphate decarboxylase
LFNG	LFNG O-fucosylpeptide 3-beta-N-acetylglucosaminyltransferase
CTIF	cap binding complex dependent translation initiation factor
SEMA3F	semaphorin 3F
S1PR1	sphingosine-1-phosphate receptor 1
CHTF18	chromosome transmission fidelity factor 18
RPS6KA4	ribosomal protein S6 kinase A4
IGDCC4	immunoglobulin superfamily DCC subclass member 4
FOXD1	forkhead box D1
SREBF1	sterol regulatory element binding transcription factor 1
INSIG1	insulin induced gene 1
PDLIM2	PDZ and LIM domain 2
ITGB3	integrin subunit beta 3
TBC1D2	TBC1 domain family member 2
DHCR7	7-dehydrocholesterol reductase
S100A10	S100 calcium binding protein A10
NAT14	N-acetyltransferase 14 (putative)
MFSD3	major facilitator superfamily domain containing 3
SPNS2	SPNS lysolipid transporter 2, sphingosine-1-phosphate
ANO8	anoctamin 8
RAPGEF3	Rap guanine nucleotide exchange factor 3
SEMA3B	semaphorin 3B
BCL2L1	BCL2 like 1
FOXC2	forkhead box C2
NSUN5P1	NSUN5 pseudogene 1
PEAR1	platelet endothelial aggregation receptor 1

MAN2C1	mannosidase alpha class 2C member 1
PDGFB	platelet derived growth factor subunit B
EPPK1	epiplakin 1
CGNL1	cingulin like 1
SH2D5	SH2 domain containing 5
AP1M2	adaptor related protein complex 1 subunit mu 2
COL27A1	collagen type XXVII alpha 1 chain
FAT3	FAT atypical cadherin 3
SYNPO	synaptopodin
EPS8L2	EPS8 like 2
CCBE1	collagen and calcium binding EGF domains 1
MACROD1	mono-ADP ribosylhydrolase 1
SHISA9	shisa family member 9
TRIM6	tripartite motif containing 6
ARHGAP23	Rho GTPase activating protein 23
LAMB3	laminin subunit beta 3
SLX1B	SLX1 homolog B, structure-specific endonuclease subunit
MEST	mesoderm specific transcript
C6orf132	chromosome 6 open reading frame 132
SYTL1	synaptotagmin like 1
GDF6	growth differentiation factor 6
TRPC4	transient receptor potential cation channel subfamily C member 4
PGM5P2	phosphoglucomutase 5 pseudogene 2
PAPLN	papilin, proteoglycan like sulfated glycoprotein
CCDC61	coiled-coil domain containing 61
HRNR	hornerin
KCNIP1	potassium voltage-gated channel interacting protein 1
ST6GAL2	ST6 beta-galactoside alpha-2,6-sialyltransferase 2
KIRREL3	kirre like nephrin family adhesion molecule 3
ADAMTS6	ADAM metallopeptidase with thrombospondin type 1 motif 6
MN1	MN1 proto-oncogene, transcriptional regulator
SPEG	striated muscle enriched protein kinase
ABCC3	ATP binding cassette subfamily C member 3
CPED1	cadherin like and PC-esterase domain containing 1
CPNE7	copine 7
RRS1	ribosome biogenesis regulator 1 homolog
AMZ1	archaelysin family metallopeptidase 1
GALK1	galactokinase 1
FBXL6	F-box and leucine rich repeat protein 6
LSM7	LSM7 homolog, U6 small nuclear RNA and mRNA degradation associated
ACCS	1-aminocyclopropane-1-carboxylate synthase homolog (inactive)
MXRA8	matrix remodeling associated 8
IER5L	immediate early response 5 like
NBEAL2	neurobeachin like 2

NAV3	neuron navigator 3
TLL1	tolloid like 1
SLC26A1	solute carrier family 26 member 1
ARHGAP22	Rho GTPase activating protein 22
EMP1	epithelial membrane protein 1
AMDHD2	amidohydrolase domain containing 2
RARRES2	retinoic acid receptor responder 2
HYI	hydroxypyruvate isomerase (putative)
LIPG	lipase G, endothelial type
TNFRSF6B	TNF receptor superfamily member 6b
KCNIP3	potassium voltage-gated channel interacting protein 3
JPH2	junctophilin 2
CDH4	cadherin 4
CCDC183	coiled-coil domain containing 183
SFN	stratifin
REXO2	RNA exonuclease 2
DNPH1	2'-deoxynucleoside 5'-phosphate N-hydrolase 1
DKK1	dickkopf WNT signaling pathway inhibitor 1
WNT2B	Wnt family member 2B
HLX	H2.0 like homeobox
SLITRK5	SLIT and NTRK like family member 5
BMP4	bone morphogenetic protein 4
TBX1	T-box transcription factor 1
ACADS	acyl-CoA dehydrogenase short chain
ID1	inhibitor of DNA binding 1
S100A2	S100 calcium binding protein A2
CDHR2	cadherin related family member 2
MIR1915HG	MIR1915 host gene
IL7R	interleukin 7 receptor
FBXW12	F-box and WD repeat domain containing 12
CSPG4P11	chondroitin sulfate proteoglycan 4 pseudogene 11
CKMT2	creatine kinase, mitochondrial 2
XDH	xanthine dehydrogenase
PGGHG	protein-glucosylgalactosylhydroxylysine glucosidase
PIF1	PIF1 5'-to-3' DNA helicase
STX1B	syntaxin 1B
CHST7	carbohydrate sulfotransferase 7
SYCE1L	synaptonemal complex central element protein 1 like
SORCS2	sortilin related VPS10 domain containing receptor 2
TSPAN18	tetraspanin 18
SPON1	spondin 1
RASA4B	RAS p21 protein activator 4B
LHPP	phospholysine phosphohistidine inorganic pyrophosphate phosphatase
KIF12	kinesin family member 12

PCSK9	proprotein convertase subtilisin/kexin type 9
KCNN4	potassium calcium-activated channel subfamily N member 4
SLC35F3	solute carrier family 35 member F3
SHANK2	SH3 and multiple ankyrin repeat domains 2
EPN3	epsin 3
S100A3	S100 calcium binding protein A3
SLC52A1	solute carrier family 52 member 1
PAK3	p21 (RAC1) activated kinase 3
ARHGEF7	Rho guanine nucleotide exchange factor 7
USP43	ubiquitin specific peptidase 43
FLG2	filaggrin 2
ACBD4	acyl-CoA binding domain containing 4
RTEL1-TNFRSF6B	RTEL1-TNFRSF6B readthrough (NMD candidate)
COL13A1	collagen type XIII alpha 1 chain
SLC16A8	solute carrier family 16 member 8
ZRSR2P1	ZRSR2 pseudogene 1
CSDC2	cold shock domain containing C2
HSD11B1L	hydroxysteroid 11-beta dehydrogenase 1 like
PSMC1P1	proteasome 26S subunit, ATPase 1 pseudogene 1
P2RY2	purinergic receptor P2Y2
METTL27	methyltransferase like 27
NTHL1	nth like DNA glycosylase 1
IKBKGP1	inhibitor of nuclear factor kappa B kinase subunit gamma pseudogene 1
ITGA2B	integrin subunit alpha 2b
FGFR4	fibroblast growth factor receptor 4
SNAI3	snail family transcriptional repressor 3
BAIAP2L2	BAR/IMD domain containing adaptor protein 2 like 2
B4GALNT4	beta-1,4-N-acetyl-galactosaminyltransferase 4
DOK7	docking protein 7
WT1	WT1 transcription factor
LMF1	lipase maturation factor 1
KLF4	KLF transcription factor 4
MAMDC4	MAM domain containing 4
SH3TC2	SH3 domain and tetratricopeptide repeats 2
VSIG8	V-set and immunoglobulin domain containing 8
GPRIN2	G protein regulated inducer of neurite outgrowth 2
GPT	glutamic--pyruvic transaminase
DPAGT1	dolichyl-phosphate N-acetylglucosaminephosphotransferase 1
P4HA3	prolyl 4-hydroxylase subunit alpha 3
ST6GALNAC5	ST6 N-acetylgalactosaminide alpha-2,6-sialyltransferase 5
INKA1	inka box actin regulator 1
HRCT1	histidine rich carboxyl terminus 1
C1orf116	chromosome 1 open reading frame 116
CAMK2A	calcium/calmodulin dependent protein kinase II alpha

NEURL2	neuralized E3 ubiquitin protein ligase 2
PTRH1	peptidyl-tRNA hydrolase 1 homolog
GPC2	glypican 2
PABPC4L	poly(A) binding protein cytoplasmic 4 like
LENG1	leukocyte receptor cluster member 1
FMC1	formation of mitochondrial complex V assembly factor 1 homolog
CCDC198	coiled-coil domain containing 198
RAB43	RAB43, member RAS oncogene family
RASAL1	RAS protein activator like 1
EDF1	endothelial differentiation related factor 1
SSR4P1	signal sequence receptor subunit 4 pseudogene 1
MYEOV	myeloma overexpressed
B3GNTL1	UDP-GlcNAc:betaGal beta-1,3-N-acetylglucosaminyltransferase like 1
DMBT1	deleted in malignant brain tumors 1
CDH16	cadherin 16
ASIC2	acid sensing ion channel subunit 2
PGM5	phosphoglucomutase 5
KCNK10	potassium two pore domain channel subfamily K member 10
LINC00431	long intergenic non-protein coding RNA 431
FGF1	fibroblast growth factor 1
PCDHGC5	protocadherin gamma subfamily C, 5
KLHL13	kelch like family member 13
EDN1	endothelin 1
PTX3	pentraxin 3
HUNK	hormonally up-regulated Neu-associated kinase
HEPACAM	hepatic and glial cell adhesion molecule
NKAIN4	sodium/potassium transporting ATPase interacting 4
GNB1L	G protein subunit beta 1 like
KLHL4	kelch like family member 4
SOAT2	sterol O-acyltransferase 2
GLYCTK	glycerate kinase
CD163L1	CD163 molecule like 1
KMT5AP1	KMT5A pseudogene 1
ADAMTS16	ADAM metallopeptidase with thrombospondin type 1 motif 16
SBK2	SH3 domain binding kinase family member 2
NOG	noggin
PROM2	prominin 2
HSD17B8	hydroxysteroid 17-beta dehydrogenase 8
SOWAHD	sosondowah ankyrin repeat domain family member D
PCDHGA6	protocadherin gamma subfamily A, 6
DNAJB13	DnaJ heat shock protein family (Hsp40) member B13
KAAG1	kidney associated DCDC2 antisense RNA 1
DYNLT4	dynein light chain Tctex-type 4
HSPD1P11	heat shock protein family D (Hsp60) member 1 pseudogene 11

MPP3	MAGUK p55 scaffold protein 3
KIF17	kinesin family member 17
CRYBG2	crystallin beta-gamma domain containing 2
DIRAS1	DIRAS family GTPase 1
PTAFR	platelet activating factor receptor
GPR39	G protein-coupled receptor 39
ACHE	acetylcholinesterase (Cartwright blood group)
MMP23B	matrix metallopeptidase 23B
PHYHIP	phytanoyl-CoA 2-hydroxylase interacting protein
DERL3	derlin 3
FKBP2	FKBP prolyl isomerase 2
NRG2	neuregulin 2
NNAT	neuronatin
USH1G	USH1 protein network component sans
ULK4P3	ULK4 pseudogene 3
CYP27C1	cytochrome P450 family 27 subfamily C member 1
DSTNP1	DSTN pseudogene 1
KLK10	kallikrein related peptidase 10
CEACAM20	CEA cell adhesion molecule 20
FRG1JP	FSHD region gene 1 family member J, pseudogene
DCST1	DC-STAMP domain containing 1
PLTP	phospholipid transfer protein
KCNJ5-AS1	KCNJ5 antisense RNA 1
APLN	apelin
CHAD	chondroadherin
MATN1	matrilin 1
BGLAP	bone gamma-carboxyglutamate protein
RIMBP3B	RIMS binding protein 3B
MAMSTR	MEF2 activating motif and SAP domain containing transcriptional regulator
INSYN2B	inhibitory synaptic factor family member 2B
NT5E	5'-nucleotidase ecto
PLAU	plasminogen activator, urokinase
IGFBP3	insulin like growth factor binding protein 3
NRP2	neuropilin 2
NELL2	neural EGFL like 2
DGKA	diacylglycerol kinase alpha
CAVIN2	caveolae associated protein 2
KCNH1	potassium voltage-gated channel subfamily H member 1
PTPRB	protein tyrosine phosphatase receptor type B
CCND1	cyclin D1
UCP2	uncoupling protein 2
HMGA2	high mobility group AT-hook 2
NFE2L3	NFE2 like bZIP transcription factor 3
CAPN2	calpain 2

HEG1	heart development protein with EGF like domains 1
DOCK4	dedicator of cytokinesis 4
RIN2	Ras and Rab interactor 2
KLF6	KLF transcription factor 6
SEMA3C	semaphorin 3C
SEMA5A	semaphorin 5A
THSD4	thrombospondin type 1 domain containing 4
CD24	CD24 molecule
MYLK	myosin light chain kinase
AMOTL2	angiomotin like 2
LMO7	LIM domain 7
LAYN	layilin
SFXN2	sideroflexin 2
NRXN3	neurexin 3
CLDN4	claudin 4
NEFL	neurofilament light chain
AOX1	aldehyde oxidase 1
KIF20A	kinesin family member 20A
PRSS23	serine protease 23
DUXAP9	double homeobox A pseudogene 9
TMSB4X	thymosin beta 4 X-linked
TJP2	tight junction protein 2
FGF5	fibroblast growth factor 5
PDGFC	platelet derived growth factor C
PCDHGC3	protocadherin gamma subfamily C, 3
TNS3	tensin 3
KRT18	keratin 18
CPA4	carboxypeptidase A4
SLC37A4	solute carrier family 37 member 4
ANXA2	annexin A2
PODXL	podocalyxin like
FAM83D	family with sequence similarity 83 member D
NCKAP5	NCK associated protein 5
NMT2	N-myristoyltransferase 2
SNAPC1	small nuclear RNA activating complex polypeptide 1
ABTB3	ankyrin repeat and BTB domain containing 3
TSPAN14	tetraspanin 14
PLAUR	plasminogen activator, urokinase receptor
DCAF12L1	DDB1 and CUL4 associated factor 12 like 1
C2CD3	C2 domain containing 3 centriole elongation regulator
POU2F2	POU class 2 homeobox 2
DRAXIN	dorsal inhibitory axon guidance protein
HLA-DOA	major histocompatibility complex, class II, DO alpha
NPNT	nephronectin

IP6K3	inositol hexakisphosphate kinase 3
BHLHE40	basic helix-loop-helix family member e40
FRMD5	FERM domain containing 5
EZR	ezrin
SERPINE1	serpin family E member 1
CHRDL1	chordin like 1
ATP8B1	ATPase phospholipid transporting 8B1
TENM4	teneurin transmembrane protein 4
P3H2	prolyl 3-hydroxylase 2
SRSF2	serine and arginine rich splicing factor 2
ADGRG1	adhesion G protein-coupled receptor G1
NIPAL4	NIPA like domain containing 4
NABP1	nucleic acid binding protein 1
MEGF9	multiple EGF like domains 9
MTMR10	myotubularin related protein 10
BCAR3	BCAR3 adaptor protein, NSP family member
TRERF1	transcriptional regulating factor 1
RPL21	ribosomal protein L21
SH3RF2	SH3 domain containing ring finger 2
BNC1	basonuclin zinc finger protein 1
ABLIM1	actin binding LIM protein 1
CMBL	carboxymethylenebutenolidase homolog
CRIM1	cysteine rich transmembrane BMP regulator 1
CD274	CD274 molecule
NHS	NHS actin remodeling regulator
HAS2	hyaluronan synthase 2
ACTN4	actinin alpha 4
CLCA2	chloride channel accessory 2
ARHGAP18	Rho GTPase activating protein 18
ARSJ	arylsulfatase family member J
PRKX	protein kinase cAMP-dependent X-linked catalytic subunit
SERTAD4	SERTA domain containing 4
NME1	NME/NM23 nucleoside diphosphate kinase 1
RMRP	RNA component of mitochondrial RNA processing endoribonuclease
ADAMTS12	ADAM metallopeptidase with thrombospondin type 1 motif 12
DHCR24	24-dehydrocholesterol reductase
F2RL2	coagulation factor II thrombin receptor like 2
SCN2A	sodium voltage-gated channel alpha subunit 2
GYPC	glycophorin C (Gerbich blood group)
NUAK1	NUAK family kinase 1
APBB2	amyloid beta precursor protein binding family B member 2

Supplemental Table 5: Downregulated DEGs (BT503 vs. DMSO (+/- LPS))

Gene Symbol	Description
CYP1B1	cytochrome P450 family 1 subfamily B member 1
STC2	stanniocalcin 2
SLC7A11	solute carrier family 7 member 11
TIPARP	TCDD inducible poly(ADP-ribose) polymerase
SHISA2	shisa family member 2
HSPA1A	heat shock protein family A (Hsp70) member 1A
CYP1A1	cytochrome P450 family 1 subfamily A member 1
SLC7A5	solute carrier family 7 member 5
ANGPTL4	angiopoietin like 4
GREM1	gremlin 1, DAN family BMP antagonist
OTUB2	OTU deubiquitinase, ubiquitin aldehyde binding 2
HSPA5	heat shock protein family A (Hsp70) member 5
DDIT3	DNA damage inducible transcript 3
HSPA1B	heat shock protein family A (Hsp70) member 1B
AHRR	aryl hydrocarbon receptor repressor
ASNS	asparagine synthetase (glutamine-hydrolyzing)
PLIN2	perilipin 2
ITGB1P1	integrin subunit beta 1 pseudogene 1
SQSTM1	sequestosome 1
NQO1	NAD(P)H quinone dehydrogenase 1
HSP90AA1	heat shock protein 90 alpha family class A member 1
TRIB3	tribbles pseudokinase 3
FTL	ferritin light chain
HSPH1	heat shock protein family H (Hsp110) member 1
ASB2	ankyrin repeat and SOCS box containing 2
IL6	interleukin 6
CCPG1	cell cycle progression 1
HSPA13	heat shock protein family A (Hsp70) member 13
CTH	cystathionine gamma-lyase
VSIG2	V-set and immunoglobulin domain containing 2
TRIM16L	tripartite motif containing 16 like (pseudogene)
BCL2L2-PABPN1	BCL2L2-PABPN1 readthrough
GABARAPL1	GABA type A receptor associated protein like 1
MT-ND4L	mitochondrially encoded NADH:ubiquinone oxidoreductase core subunit 4L
ZFAND2A	zinc finger AN1-type containing 2A
TSC22D3	TSC22 domain family member 3
IFRD1	interferon related developmental regulator 1
CREBRF	CREB3 regulatory factor
ZNF473	zinc finger protein 473
FAM107B	family with sequence similarity 107 member B
CHAC1	ChaC glutathione specific gamma-glutamylcyclotransferase 1

DHRS3	dehydrogenase/reductase 3
OSGIN1	oxidative stress induced growth inhibitor 1
CCL2	C-C motif chemokine ligand 2
LPXN	leupaxin
PPP1R15A	protein phosphatase 1 regulatory subunit 15A
NFE2L2	NFE2 like bZIP transcription factor 2
INA	internexin neuronal intermediate filament protein alpha
SLU7	SLU7 homolog, splicing factor
HMOX1	heme oxygenase 1
KLHL24	kelch like family member 24
LRIF1	ligand dependent nuclear receptor interacting factor 1
TRIM16	tripartite motif containing 16
KCNK3	potassium two pore domain channel subfamily K member 3
CXCL8	C-X-C motif chemokine ligand 8
CASP4	caspase 4
CENPS-CORT	CENPS-CORT readthrough
ADGRF1	adhesion G protein-coupled receptor F1
SAA2	serum amyloid A2
DLX2	distal-less homeobox 2
IGF2	insulin like growth factor 2
ARRDC4	arrestin domain containing 4
CENPQ	centromere protein Q
H2BC12	H2B clustered histone 12
QRICH2	glutamine rich 2
SC5D	sterol-C5-desaturase
LUM	lumican
MIA2	MIA SH3 domain ER export factor 2
CYTIP	cytohesin 1 interacting protein
ZNF511-PRAP1	ZNF511-PRAP1 readthrough
ZNF273	zinc finger protein 273
GFPT2	glutamine-fructose-6-phosphate transaminase 2
CHRM4	cholinergic receptor muscarinic 4
SPX	spexin hormone
CXCL1	C-X-C motif chemokine ligand 1
TRIM36	tripartite motif containing 36
IL1A	interleukin 1 alpha
CLEC4E	C-type lectin domain family 4 member E
ATG4A	autophagy related 4A cysteine peptidase
FAM9C	family with sequence similarity 9 member C
CPEB3	cytoplasmic polyadenylation element binding protein 3
ZNF267	zinc finger protein 267
TIGD7	tigger transposable element derived 7
ZNF823	zinc finger protein 823
PIR	pirin

SH3GL2	SH3 domain containing GRB2 like 2, endophilin A1
ZNF724	zinc finger protein 724
HSPE1-MOB4	HSPE1-MOB4 readthrough
RADX	RPA1 related single stranded DNA binding protein, X-linked
SNAI1	snail family transcriptional repressor 1
LEKR1	leucine, glutamate and lysine rich 1
H4C9	H4 clustered histone 9
FZD3	frizzled class receptor 3
FZD9	frizzled class receptor 9
RPL13P12	ribosomal protein L13 pseudogene 12
SYS1-DBNDD2	SYS1-DBNDD2 readthrough (NMD candidate)
BMF	Bcl2 modifying factor
CXCL11	C-X-C motif chemokine ligand 11
H3P6	H3 histone pseudogene 6
PTGES3L-AARSD1	PTGES3L-AARSD1 readthrough
KRT34	keratin 34
BATF2	basic leucine zipper ATF-like transcription factor 2
CYP27B1	cytochrome P450 family 27 subfamily B member 1
MAFA	MAF bZIP transcription factor A
KLRG1	killer cell lectin like receptor G1
BBS12	Bardet-Biedl syndrome 12
COLQ	collagen like tail subunit of asymmetric acetylcholinesterase
HOXC8	homeobox C8
PDE8B	phosphodiesterase 8B
PRRG3	proline rich and Gla domain 3
HSPA6	heat shock protein family A (Hsp70) member 6
BCAS1	brain enriched myelin associated protein 1
LRRC49	leucine rich repeat containing 49
FAM47E-STBD1	FAM47E-STBD1 readthrough
CRABP2	cellular retinoic acid binding protein 2
ZNF100	zinc finger protein 100
TMED7-TICAM2	TMED7-TICAM2 readthrough
HLA-DMB	major histocompatibility complex, class II, DM beta
ZNF570	zinc finger protein 570
FRG1BP	FSHD region gene 1 family member B, pseudogene
TMEM179	transmembrane protein 179
GKAP1	G kinase anchoring protein 1
FOS	Fos proto-oncogene, AP-1 transcription factor subunit
ZCCHC12	zinc finger CCHC-type containing 12
IL1B	interleukin 1 beta
MICOS10-NBL1	MICOS10-NBL1 readthrough
FMC1-LUC7L2	FMC1-LUC7L2 readthrough
AOC2	amine oxidase copper containing 2

CCDC181	coiled-coil domain containing 181
GYG2P1	glycogenin 2 pseudogene 1
DNAJC27	DnaJ heat shock protein family (Hsp40) member C27
MYO7A	myosin VIIA
IRGM	immunity related GTPase M
ULBP3	UL16 binding protein 3
TMED10P1	transmembrane p24 trafficking protein 10 pseudogene 1
RND1	Rho family GTPase 1
ZNF695	zinc finger protein 695
GPR68	G protein-coupled receptor 68
RGPD6	RANBP2 like and GRIP domain containing 6
MPZL3	myelin protein zero like 3
BTBD8	BTB domain containing 8
ATP1B2	ATPase Na+/K+ transporting subunit beta 2
PLA2G4C	phospholipase A2 group IVC
CAB39L	calcium binding protein 39 like
ELAVL3	ELAV like RNA binding protein 3
SLC6A9	solute carrier family 6 member 9
CTAGE8	CTAGE family member 8
DPY19L1P1	DPY19L1 pseudogene 1
MMP16	matrix metallopeptidase 16
XKR9	XK related 9
RNASEK-C17orf49	RNASEK-C17orf49 readthrough
TIGD4	tigger transposable element derived 4
ZNF214	zinc finger protein 214
IQCK	IQ motif containing K
SHE	Src homology 2 domain containing E
FGF23	fibroblast growth factor 23
CKMT1B	creatine kinase, mitochondrial 1B
TAS2R4	taste 2 receptor member 4
EFHD1	EF-hand domain family member D1
GAREM1	GRB2 associated regulator of MAPK1 subtype 1
STIMATE-MUSTN1	STIMATE-MUSTN1 readthrough
EIF3CL	eukaryotic translation initiation factor 3 subunit C like
C3orf33	chromosome 3 open reading frame 33
CPA2	carboxypeptidase A2
ZFP37	ZFP37 zinc finger protein
UBE2FP1	UBE2F pseudogene 1
PPM1L	protein phosphatase, Mg2+/Mn2+ dependent 1L
GTF2IRD2P1	GTF2I repeat domain containing 2 pseudogene 1
SMIM11	small integral membrane protein 11
SLC3A1	solute carrier family 3 member 1
SLC43A1	solute carrier family 43 member 1

HPCAL4	hippocalcin like 4
ZNF568	zinc finger protein 568
TEN1-CDK3	TEN1-CDK3 readthrough (NMD candidate)
BEX2	brain expressed X-linked 2
CNTF	ciliary neurotrophic factor
GP1BB	glycoprotein Ib platelet subunit beta
OGN	osteoglycin
MAP2	microtubule associated protein 2
METAP2	methionyl aminopeptidase 2
INO80B-WBP1	INO80B-WBP1 readthrough (NMD candidate)
EML1	EMAP like 1
ZNF836	zinc finger protein 836
ZNF43	zinc finger protein 43
RAB39B	RAB39B, member RAS oncogene family
C12orf50	chromosome 12 open reading frame 50
LPA	lipoprotein(a)
CXCL2	C-X-C motif chemokine ligand 2
SRXN1	sulfiredoxin 1
TTN	titin
ATF3	activating transcription factor 3
SEPHS2	selenophosphate synthetase 2
SLC3A2	solute carrier family 3 member 2
ANKRD13C	ankyrin repeat domain 13C
CXCL3	C-X-C motif chemokine ligand 3
TPCN1	two pore segment channel 1
KRTAP2-3	keratin associated protein 2-3
FREM2	FRAS1 related extracellular matrix 2
HLA-E	major histocompatibility complex, class I, E
MAFG	MAF bZIP transcription factor G
MYBL2	MYB proto-oncogene like 2
RASSF1	Ras association domain family member 1
TPRA1	transmembrane protein adipocyte associated 1
GPR153	G protein-coupled receptor 153
HDAC11	histone deacetylase 11
RHBDL3	rhomboid like 3
AGTR1	angiotensin II receptor type 1
FBN1	fibrillin 1
TBRG1	transforming growth factor beta regulator 1
NCF2	neutrophil cytosolic factor 2
PSENEN	presenilin enhancer, gamma-secretase subunit
GLA	galactosidase alpha
NAT8	N-acetyltransferase 8 (putative)
DNAJB9	DnaJ heat shock protein family (Hsp40) member B9
IER3	immediate early response 3

USH2A	usherin
UNKL	unk like zinc finger
FLG	filaggrin
FGFR1	fibroblast growth factor receptor 1
SLC66A1	solute carrier family 66 member 1
MAP1LC3B	microtubule associated protein 1 light chain 3 beta
INPP5K	inositol polyphosphate-5-phosphatase K
SMCR8	SMCR8-C9orf72 complex subunit

Supplemental Table 6: Upregulated DEGs (BT503 vs. DMSO in nonpermissive conditions (37°C), relative to permissive conditions (33°C))

Gene Symbol	Description
C3	complement C3
CPA4	carboxypeptidase A4
CDKN1A	cyclin dependent kinase inhibitor 1A
SERPINE1	serpin family E member 1
GDF15	growth differentiation factor 15
NT5E	5'-nucleotidase ecto
LTBP2	latent transforming growth factor beta binding protein 2
LTBP3	latent transforming growth factor beta binding protein 3
PHLDA1	pleckstrin homology like domain family A member 1
PSTPIP2	proline-serine-threonine phosphatase interacting protein 2
TRIM22	tripartite motif containing 22
PLAT	plasminogen activator, tissue type
CLDN1	claudin 1
CPT1A	carnitine palmitoyltransferase 1A
BTG2	BTG anti-proliferation factor 2
MDM2	MDM2 proto-oncogene
CLCA2	chloride channel accessory 2
SLC7A14	solute carrier family 7 member 14
INPP5D	inositol polyphosphate-5-phosphatase D
DRAM1	DNA damage regulated autophagy modulator 1
SULF2	sulfatase 2
FBN1	fibrillin 1
CRYAB	crystallin alpha B
SLC7A11	solute carrier family 7 member 11
EDA2R	ectodysplasin A2 receptor
ACTA2	actin alpha 2, smooth muscle
CCND1	cyclin D1
TNFRSF10B	TNF receptor superfamily member 10b
SPATA18	spermatogenesis associated 18
CD82	CD82 molecule
ANKRD1	ankyrin repeat domain 1
HSPA1A	heat shock protein family A (Hsp70) member 1A
FAS	Fas cell surface death receptor
FASN	fatty acid synthase
STC2	stanniocalcin 2
IGFBP7	insulin like growth factor binding protein 7
INKA2	inka box actin regulator 2
HSPA4L	heat shock protein family A (Hsp70) member 4 like
DDX60	DExD/H-box helicase 60
TGFA	transforming growth factor alpha
DRAXIN	dorsal inhibitory axon guidance protein
WNT5B	Wnt family member 5B
DUSP6	dual specificity phosphatase 6

ETV5	ETS variant transcription factor 5
SPRY4	sprouty RTK signaling antagonist 4
EFNB1	ephrin B1
MARCHF4	membrane associated ring-CH-type finger 4
PLK3	polo like kinase 3
PAG1	phosphoprotein membrane anchor with glycosphingolipid microdomains 1
HMOX1	heme oxygenase 1
LACC1	laccase domain containing 1
PHLDA3	pleckstrin homology like domain family A member 3
CSMD3	CUB and Sushi multiple domains 3
ITGA11	integrin subunit alpha 11
NTN1	netrin 1
TP53I3	tumor protein p53 inducible protein 3
HLA-DOA	major histocompatibility complex, class II, DO alpha
SLCO4A1	solute carrier organic anion transporter family member 4A1
DHRS2	dehydrogenase/reductase 2
CA12	carbonic anhydrase 12
CHAC1	ChaC glutathione specific gamma-glutamylcyclotransferase 1
HSPE1-MOB4	HSPE1-MOB4 readthrough
DNAH3	dynein axonemal heavy chain 3
DNAI3	dynein axonemal intermediate chain 3
RFLNA	refilin A
CEACAM1	CEA cell adhesion molecule 1
ABCB1	ATP binding cassette subfamily B member 1
NOTCH3	notch receptor 3
CFH	complement factor H
ZDHHC14	zinc finger DHHC-type palmitoyltransferase 14
ACER2	alkaline ceramidase 2
SAA1	serum amyloid A1
FOXO1	forkhead box O1
NSG1	neuronal vesicle trafficking associated 1
GREB1	growth regulating estrogen receptor binding 1
TP53I11	tumor protein p53 inducible protein 11
PLCL2	phospholipase C like 2
TMEM59L	transmembrane protein 59 like
PPP1R14C	protein phosphatase 1 regulatory inhibitor subunit 14C
PTCHD4	patched domain containing 4
CXCL11	C-X-C motif chemokine ligand 11
FLRT2	fibronectin leucine rich transmembrane protein 2
SRGAP3	SLIT-ROBO Rho GTPase activating protein 3
NPPB	natriuretic peptide B
ABCA12	ATP binding cassette subfamily A member 12
CYGB	cytoglobin
AK5	adenylate kinase 5

CHRM4	cholinergic receptor muscarinic 4
RHBDL3	rhomboid like 3
GRHL3	grainyhead like transcription factor 3
TBX2	T-box transcription factor 2
GABBR2	gamma-aminobutyric acid type B receptor subunit 2
NECTIN4	nectin cell adhesion molecule 4
FOXA1	forkhead box A1
BLNK	B cell linker
MICOS10-NBL1	MICOS10-NBL1 readthrough
IL6	interleukin 6
POU2F2	POU class 2 homeobox 2
RTL5	retrotransposon Gag like 5
GPR87	G protein-coupled receptor 87
TNFRSF14	TNF receptor superfamily member 14
UBE2L6	ubiquitin conjugating enzyme E2 L6
KCNK3	potassium two pore domain channel subfamily K member 3
CXCL1	C-X-C motif chemokine ligand 1
NPTXR	neuronal pentraxin receptor
ANGPTL4	angiopoietin like 4
KRTAP2-3	keratin associated protein 2-3
TRIM55	tripartite motif containing 55
CXCL8	C-X-C motif chemokine ligand 8
CDH10	cadherin 10
ANKRD29	ankyrin repeat domain 29
ARRDC4	arrestin domain containing 4
FAM13C	family with sequence similarity 13 member C
GATA6	GATA binding protein 6
FEZ1	fasciculation and elongation protein zeta 1
LZTS1	leucine zipper tumor suppressor 1
DOCK4	dedicator of cytokinesis 4
DNAH12	dynein axonemal heavy chain 12
SUGCT	succinyl-CoA:glutarate-CoA transferase
HERC5	HECT and RLD domain containing E3 ubiquitin protein ligase 5
IGF2	insulin like growth factor 2
OAS1	2'-5'-oligoadenylate synthetase 1
EMILIN3	elastin microfibril interfacer 3
MMRN2	multimerin 2
H2AC11	H2A clustered histone 11
TLR4	toll like receptor 4
SCN4B	sodium voltage-gated channel beta subunit 4
ST6GAL1	ST6 beta-galactoside alpha-2,6-sialyltransferase 1
SGIP1	SH3GL interacting endocytic adaptor 1
CLTRN	collectrin, amino acid transport regulator
SCN2A	sodium voltage-gated channel alpha subunit 2

RTN1	reticulon 1
LEF1	lymphoid enhancer binding factor 1
SERPINB7	serpin family B member 7
SHANK1	SH3 and multiple ankyrin repeat domains 1
ACSS1	acyl-CoA synthetase short chain family member 1
HAS3	hyaluronan synthase 3
PLEKHG1	pleckstrin homology and RhoGEF domain containing G1
KANK3	KN motif and ankyrin repeat domains 3
CLEC4E	C-type lectin domain family 4 member E
RNASE7	ribonuclease A family member 7
USP18	ubiquitin specific peptidase 18
FBXO32	F-box protein 32
PDE4C	phosphodiesterase 4C
HSPA6	heat shock protein family A (Hsp70) member 6
SPRY1	sprouty RTK signaling antagonist 1
TMEM179	transmembrane protein 179
ANK1	ankyrin 1
SAA2	serum amyloid A2
H2BC5	H2B clustered histone 5
FAM43A	family with sequence similarity 43 member A
IRGM	immunity related GTPase M
GAL3ST4	galactose-3-O-sulfotransferase 4
NR2F1	nuclear receptor subfamily 2 group F member 1
CCND2	cyclin D2
COL13A1	collagen type XIII alpha 1 chain
CCR4	C-C motif chemokine receptor 4
CNOT6	CCR4-NOT transcription complex subunit 6
HBA1	hemoglobin subunit alpha 1
SLC15A3	solute carrier family 15 member 3
SHC3	SHC adaptor protein 3
SLC27A2	solute carrier family 27 member 2
HES1	hes family bHLH transcription factor 1
GATD3	glutamine amidotransferase class 1 domain containing 3
NRARP	NOTCH regulated ankyrin repeat protein
RTL9	retrotransposon Gag like 9
POU3F1	POU class 3 homeobox 1
FAM184A	family with sequence similarity 184 member A
REEP1	receptor accessory protein 1
LRP3	LDL receptor related protein 3
CASP1	caspase 1
OPCML	opioid binding protein/cell adhesion molecule like
LURAP1L	leucine rich adaptor protein 1 like
CD177	CD177 molecule
PNMA2	PNMA family member 2

ANOS1	anosmin 1
PADI3	peptidyl arginine deiminase 3
TMEM158	transmembrane protein 158
SCN5A	sodium voltage-gated channel alpha subunit 5
SMC1B	structural maintenance of chromosomes 1B
LYPD6B	LY6/PLAUR domain containing 6B
APOBEC3H	apolipoprotein B mRNA editing enzyme catalytic subunit 3H
IFIH1	interferon induced with helicase C domain 1
KLHL30	kelch like family member 30
DUSP4	dual specificity phosphatase 4
LRRC26	leucine rich repeat containing 26
SNAP25	synaptosome associated protein 25
ANKRD20A11P	ankyrin repeat domain 20 family member A11, pseudogene
SERPING1	serpin family G member 1
ATP1A3	ATPase Na+/K+ transporting subunit alpha 3
TMOD1	tropomodulin 1
WSCD1	WSC domain containing 1
FBXL16	F-box and leucine rich repeat protein 16
HTRA1	HtrA serine peptidase 1
TGFBR3	transforming growth factor beta receptor 3
EPHX2	epoxide hydrolase 2
TLR3	toll like receptor 3
LAMP3	lysosomal associated membrane protein 3
CYP4F25P	cytochrome P450 family 4 subfamily F member 25, pseudogene
COL4A4	collagen type IV alpha 4 chain
MYEOV	myeloma overexpressed
STOX2	storkhead box 2
TG	thyroglobulin
ENHO	energy homeostasis associated
SLC43A1	solute carrier family 43 member 1
NRCAM	neuronal cell adhesion molecule
CEACAM22P	CEA cell adhesion molecule 22, pseudogene
LYNX1	Ly6/neurotoxin 1
KCNN2	potassium calcium-activated channel subfamily N member 2
CEMP1	cementum protein 1
CATSPERD	cation channel sperm associated auxiliary subunit delta
CELF2	CUGBP Elav-like family member 2
HES2	hes family bHLH transcription factor 2
SMOC1	SPARC related modular calcium binding 1
BST2	bone marrow stromal cell antigen 2
NBEAP1	neurobeachin pseudogene 1
HHATL	hedgehog acyltransferase like
CLDN16	claudin 16
SERPINB2	serpin family B member 2

PREX1	phosphatidylinositol-3,4,5-trisphosphate dependent Rac exchange factor 1
XKR7	XK related 7
ALOX5	arachidonate 5-lipoxygenase
HMCN2	hemicentin 2
CARD6	caspase recruitment domain family member 6
ACVR1C	activin A receptor type 1C
CXCL6	C-X-C motif chemokine ligand 6
C5AR1	complement C5a receptor 1
ZNF560	zinc finger protein 560
CYTIP	cytohesin 1 interacting protein
TENT5C	terminal nucleotidyltransferase 5C
DDO	D-aspartate oxidase
DLL4	delta like canonical Notch ligand 4
OASL	2'-5'-oligoadenylate synthetase like
GRIP2	glutamate receptor interacting protein 2
RANBP3L	RAN binding protein 3 like
C11orf96	chromosome 11 open reading frame 96
TYRP1	tyrosinase related protein 1
OR2I1P	olfactory receptor family 2 subfamily I member 1 pseudogene
GRIN1	glutamate ionotropic receptor NMDA type subunit 1
GPRIN1	G protein regulated inducer of neurite outgrowth 1
PSG9	pregnancy specific beta-1-glycoprotein 9
BCAS1	brain enriched myelin associated protein 1
SLC28A3	solute carrier family 28 member 3
ADAMTS14	ADAM metallopeptidase with thrombospondin type 1 motif 14
AKR1B10	aldo-keto reductase family 1 member B10
FBLN5	fibulin 5
LHX3	LIM homeobox 3
MYOCD	myocardin
COL25A1	collagen type XXV alpha 1 chain
CSF2	colony stimulating factor 2
PDK4	pyruvate dehydrogenase kinase 4
SYN1	synapsin I
KLRG2	killer cell lectin like receptor G2
PSG3	pregnancy specific beta-1-glycoprotein 3
ZNF488	zinc finger protein 488
FBP2	fructose-bisphosphatase 2
KHSRP	KH-type splicing regulatory protein
MUC5AC	mucin 5AC, oligomeric mucus/gel-forming
PSG1	pregnancy specific beta-1-glycoprotein 1
PSG2	pregnancy specific beta-1-glycoprotein 2
PLD5	phospholipase D family member 5
ENTREP1	endosomal transmembrane epsin interactor 1
MAFA	MAF bZIP transcription factor A

KLRG1	killer cell lectin like receptor G1
OFCC1	orofacial cleft 1 candidate 1 (pseudogene)
MT1G	metallothionein 1G
FOLR3	folate receptor gamma
PGBD5	piggyBac transposable element derived 5
IL15RA	interleukin 15 receptor subunit alpha
VMO1	vitelline membrane outer layer 1 homolog
LRRC66	leucine rich repeat containing 66
TFEC	transcription factor EC
PAX5	paired box 5
CEACAM20	CEA cell adhesion molecule 20
HSD3BP5	hydroxy-delta-5-steroid dehydrogenase, 3 beta, pseudogene 5
SPECC1L-ADORA2A	SPECC1L-ADORA2A readthrough (NMD candidate)
RGPD1	RANBP2 like and GRIP domain containing 1
SHC2	SHC adaptor protein 2
C19orf38	chromosome 19 open reading frame 38
KRT34	keratin 34
PPP1R16B	protein phosphatase 1 regulatory subunit 16B
TEX19	testis expressed 19
GTF2IRD2P1	GTF2I repeat domain containing 2 pseudogene 1
PDE2A	phosphodiesterase 2A
ZNF511-PRAP1	ZNF511-PRAP1 readthrough
TINCR	TINCR ubiquitin domain containing
RAB39B	RAB39B, member RAS oncogene family
EIF3CL	eukaryotic translation initiation factor 3 subunit C like
RBP4	retinol binding protein 4
CCDC168	coiled-coil domain containing 168
NPIPP1	nuclear pore complex interacting protein pseudogene 1
H4C14	H4 clustered histone 14
PCDHB2	protocadherin beta 2
NTNG2	netrin G2
OAS2	2'-5'-oligoadenylate synthetase 2
GAST	gastrin
H4C15	H4 clustered histone 15
SP140	SP140 nuclear body protein
CORO7-PAM16	CORO7-PAM16 readthrough
MDGA1	MAM domain containing glycosylphosphatidylinositol anchor 1
SESN1	sestrin 1
NPDC1	neural proliferation, differentiation and control 1
CSF1	colony stimulating factor 1
ZMAT3	zinc finger matrin-type 3
PTPRU	protein tyrosine phosphatase receptor type U
MAST4	microtubule associated serine/threonine kinase family member 4

FDXR	ferredoxin reductase
MAMDC2	MAM domain containing 2
DUSP5	dual specificity phosphatase 5
BBC3	BCL2 binding component 3
ICOSLG	inducible T cell costimulator ligand
IFI6	interferon alpha inducible protein 6
VWCE	von Willebrand factor C and EGF domains
WNK4	WNK lysine deficient protein kinase 4
SLC4A11	solute carrier family 4 member 11
SLX1B	SLX1 homolog B, structure-specific endonuclease subunit
BTBD19	BTB domain containing 19
BRSK2	BR serine/threonine kinase 2
EMP1	epithelial membrane protein 1
CAVIN2	caveolae associated protein 2
SHC4	SHC adaptor protein 4
KCNJ12	potassium inwardly rectifying channel subfamily J member 12
SLC16A12	solute carrier family 16 member 12
IFI27	interferon alpha inducible protein 27
F8A2	coagulation factor VIII associated 2
PDE4A	phosphodiesterase 4A
ALDH1A3	aldehyde dehydrogenase 1 family member A3
STX1A	syntaxin 1A
KLLN	killin, p53 regulated DNA replication inhibitor
HRNR	hornerin
COL17A1	collagen type XVII alpha 1 chain
IL7	interleukin 7
ASB2	ankyrin repeat and SOCS box containing 2
SRPX2	sushi repeat containing protein X-linked 2
KCTD12	potassium channel tetramerization domain containing 12
NHLH2	nescient helix-loop-helix 2
ACHE	acetylcholinesterase (Cartwright blood group)
SLC52A1	solute carrier family 52 member 1
GLS2	glutaminase 2
TBX10	T-box transcription factor 10
TRPV2	transient receptor potential cation channel subfamily V member 2
H4C8	H4 clustered histone 8
ZACN	zinc activated ion channel
RPL13AP20	ribosomal protein L13a pseudogene 20
DYNLT4	dynein light chain Tctex-type 4
ADCY1	adenylate cyclase 1
RPL24P8	RPL24 pseudogene 8
DKK1	dickkopf WNT signaling pathway inhibitor 1
CALML6	calmodulin like 6
PRODH	proline dehydrogenase 1

GAD1	glutamate decarboxylase 1
RNF152	ring finger protein 152
FGF1	fibroblast growth factor 1
HEPACAM	hepatic and glial cell adhesion molecule
CMPK2	cytidine/uridine monophosphate kinase 2
FRMPD2	FERM and PDZ domain containing 2
APOL3	apolipoprotein L3
KNDC1	kinase non-catalytic C-lobe domain containing 1
CCDC187	coiled-coil domain containing 187
FLG2	filaggrin 2
CHST15	carbohydrate sulfotransferase 15
RPSAP52	ribosomal protein SA pseudogene 52
ARC	activity regulated cytoskeleton associated protein
NOL3	nucleolar protein 3
CYP26B1	cytochrome P450 family 26 subfamily B member 1
NOS1AP	nitric oxide synthase 1 adaptor protein
INKA1	inka box actin regulator 1
KRT23	keratin 23
RASD2	RASD family member 2
SMIM10L2A	small integral membrane protein 10 like 2A
DUSP13B	dual specificity phosphatase 13B
CNTFR	ciliary neurotrophic factor receptor
GLDC	glycine decarboxylase
KCTD16	potassium channel tetramerization domain containing 16
TMEM151A	transmembrane protein 151A
DMBT1	deleted in malignant brain tumors 1
KCNQ3	potassium voltage-gated channel subfamily Q member 3
TTC6	tetratricopeptide repeat domain 6
PTPRN	protein tyrosine phosphatase receptor type N
NPR1	natriuretic peptide receptor 1
BMPER	BMP binding endothelial regulator
EGR2	early growth response 2
NT5C1B	5'-nucleotidase, cytosolic IB
ALOX15	arachidonate 15-lipoxygenase
DUX4L9	double homeobox 4 like 9 (pseudogene)
IFITM10	interferon induced transmembrane protein 10
WNT11	Wnt family member 11
TMEM200A	transmembrane protein 200A
ADAM21	ADAM metallopeptidase domain 21
PSG8	pregnancy specific beta-1-glycoprotein 8
USH1G	USH1 protein network component sans
TP53AIP1	tumor protein p53 regulated apoptosis inducing protein 1
GBP2	guanylate binding protein 2
LRRN2	leucine rich repeat neuronal 2

KLRC1	killer cell lectin like receptor C1
CASKIN1	CASK interacting protein 1
MGAT4A	alpha-1,3-mannosyl-glycoprotein 4-beta-N-acetylglucosaminyltransferase A
ANKRD30B	ankyrin repeat domain 30B
FGF17	fibroblast growth factor 17
GPR65	G protein-coupled receptor 65
IQSEC3	IQ motif and Sec7 domain ArfGEF 3
DQX1	DEAQ-box RNA dependent ATPase 1
LKAAEAR1	LKAAEAR motif containing 1
PSMC1P1	proteasome 26S subunit, ATPase 1 pseudogene 1
MEP1A	meprin A subunit alpha
PALM2	paralemmin 2
VSTM2L	V-set and transmembrane domain containing 2 like
THBD	thrombomodulin
CNTNAP3P2	CNTNAP3 pseudogene 2
HSPB2-C11orf52	HSPB2-C11orf52 readthrough (NMD candidate)
ARHGEF4	Rho guanine nucleotide exchange factor 4
OSR1	odd-skipped related transcription factor 1
OSER1	oxidative stress responsive serine rich 1
OXSR1	oxidative stress responsive kinase 1
GALNT18	polypeptide N-acetylgalactosaminyltransferase 18
SCGB1A1	secretoglobin family 1A member 1
CCIN	calicin
AGAP13P	ArfGAP with GTPase domain, ankyrin repeat and PH domain 13, pseudogene

Supplemental Table 7: Downregulated DEGs (BT503 vs. DMSO in nonpermissive conditions (37°C), relative to permissive conditions (33°C))

Gene Symbol	Description
SCD	stearoyl-CoA desaturase
SLC22A5	solute carrier family 22 member 5
RARRES2	retinoic acid receptor responder 2
CDH6	cadherin 6
IGFBP3	insulin like growth factor binding protein 3
MMP2	matrix metallopeptidase 2
CXCL12	C-X-C motif chemokine ligand 12
HMGCS1	3-hydroxy-3-methylglutaryl-CoA synthase 1
CNN1	calponin 1
CFI	complement factor I
PRICKLE1	prickle planar cell polarity protein 1
INSIG1	insulin induced gene 1
MN1	MN1 proto-oncogene, transcriptional regulator
LDLR	low density lipoprotein receptor
WNT2B	Wnt family member 2B
SAMD11	sterile alpha motif domain containing 11
BMF	Bcl2 modifying factor
MTUS1	microtubule associated scaffold protein 1
PDGFB	platelet derived growth factor subunit B
RBM3	RNA binding motif protein 3
EPHA5	EPH receptor A5
SCNN1A	sodium channel epithelial 1 subunit alpha
ITGA4	integrin subunit alpha 4
CD24	CD24 molecule
S1PR1	sphingosine-1-phosphate receptor 1
ERBB3	erb-b2 receptor tyrosine kinase 3
IGFBP6	insulin like growth factor binding protein 6
TNFRSF19	TNF receptor superfamily member 19
LRRC61	leucine rich repeat containing 61
AHRR	aryl hydrocarbon receptor repressor
ZBED10P	zinc finger BED-type containing 10, pseudogene
SEMA5A	semaphorin 5A
SREBF1	sterol regulatory element binding transcription factor 1
KRT80	keratin 80
HAPLN1	hyaluronan and proteoglycan link protein 1
PDZK1	PDZ domain containing 1
PTGIS	prostaglandin I2 synthase
CRISPLD2	cysteine rich secretory protein LCCL domain containing 2
HMCN1	hemicentin 1
SERPINA1	serpin family A member 1

TCF4	transcription factor 4
TCF7L2	transcription factor 7 like 2
COL8A1	collagen type VIII alpha 1 chain
LOXL1	lysyl oxidase like 1
ADAM19	ADAM metallopeptidase domain 19
KCNIP1	potassium voltage-gated channel interacting protein 1
GFRA1	GDNF family receptor alpha 1
FASN	fatty acid synthase
TENM2	teneurin transmembrane protein 2
COL5A1	collagen type V alpha 1 chain
CARD10	caspase recruitment domain family member 10
ELF3	E74 like ETS transcription factor 3
EFNB3	ephrin B3
CDKN2B	cyclin dependent kinase inhibitor 2B
SIM2	SIM bHLH transcription factor 2
GLIS2	GLIS family zinc finger 2
CDKN1C	cyclin dependent kinase inhibitor 1C
DENND2A	DENN domain containing 2A
TRIM6	tripartite motif containing 6
MUC1	mucin 1, cell surface associated
SHANK2	SH3 and multiple ankyrin repeat domains 2
ANXA3	annexin A3
AIF1L	allograft inflammatory factor 1 like
GSTM4	glutathione S-transferase mu 4
LAMA5	laminin subunit alpha 5
PROS1	protein S
METRNL	meteorin like, glial cell differentiation regulator
TUBB2B	tubulin beta 2B class IIb
BCAM	basal cell adhesion molecule (Lutheran blood group)
BCAT2	branched chain amino acid transaminase 2
LUM	lumican
AP1M2	adaptor related protein complex 1 subunit mu 2
LFNG	LFNG O-fucosylpeptide 3-beta-N-acetylglucosaminyltransferase
RARG	retinoic acid receptor gamma
COLEC12	collectin subfamily member 12
EDN2	endothelin 2
CAMKK1	calcium/calmodulin dependent protein kinase 1
LHPP	phospholysine phosphohistidine inorganic pyrophosphate phosphatase
ADGRB3	adhesion G protein-coupled receptor B3
MXRA8	matrix remodeling associated 8
CCBE1	collagen and calcium binding EGF domains 1
FHDC1	FH2 domain containing 1
ARHGAP11B	Rho GTPase activating protein 11B
KIF12	kinesin family member 12

NES	nestin
MYL3	myosin light chain 3
ADAMTS15	ADAM metallopeptidase with thrombospondin type 1 motif 15
TSPOAP1	TSPO associated protein 1
SLC1A3	solute carrier family 1 member 3
PCSK9	proprotein convertase subtilisin/kexin type 9
SUSD4	sushi domain containing 4
FJX1	four-jointed box kinase 1
ANK3	ankyrin 3
KBTBD11	kelch repeat and BTB domain containing 11
SGK2	serum/glucocorticoid regulated kinase 2
SGK3	serum/glucocorticoid regulated kinase family member 3
C1QL4	complement C1q like 4
IGFBP5	insulin like growth factor binding protein 5
THBS3	thrombospondin 3
COL9A2	collagen type IX alpha 2 chain
GXYLT2	glucoside xylosyltransferase 2
ATOH8	atonal bHLH transcription factor 8
DHCR7	7-dehydrocholesterol reductase
ANO9	anoctamin 9
SHF	Src homology 2 domain containing F
CDH16	cadherin 16
LHX1	LIM homeobox 1
RNF43	ring finger protein 43
ELFN2	extracellular leucine rich repeat and fibronectin type III domain containing 2
GDF6	growth differentiation factor 6
C1orf116	chromosome 1 open reading frame 116
TRIM58	tripartite motif containing 58
RUNX1T1	RUNX1 partner transcriptional co-repressor 1
CGN	cingulin
KIRREL3	kirre like nephrin family adhesion molecule 3
PIF1	PIF1 5'-to-3' DNA helicase
ESRP2	epithelial splicing regulatory protein 2
SLC29A2	solute carrier family 29 member 2
UNC13D	unc-13 homolog D
PLEKHG4B	pleckstrin homology and RhoGEF domain containing G4B
FGFR4	fibroblast growth factor receptor 4
GPR162	G protein-coupled receptor 162
GPER1	G protein-coupled estrogen receptor 1
ARHGAP28	Rho GTPase activating protein 28
ERLEC1P1	endoplasmic reticulum lectin 1 pseudogene 1
ST6GAL2	ST6 beta-galactoside alpha-2,6-sialyltransferase 2
PTH1R	parathyroid hormone 1 receptor
CHN1	chimerin 1

RBP1	retinol binding protein 1
ARID4A	AT-rich interaction domain 4A
SGCD	sarcoglycan delta
SLITRK5	SLIT and NTRK like family member 5
TET1	tet methylcytosine dioxygenase 1
SLC27A3	solute carrier family 27 member 3
UNC5C	unc-5 netrin receptor C
GPRIN2	G protein regulated inducer of neurite outgrowth 2
ADAMTS6	ADAM metallopeptidase with thrombospondin type 1 motif 6
GJB4	gap junction protein beta 4
BMPR1B	bone morphogenetic protein receptor type 1B
LRRC7	leucine rich repeat containing 7
MEX3A	mex-3 RNA binding family member A
CD37	CD37 molecule
TRPM2	transient receptor potential cation channel subfamily M member 2
VWA1	von Willebrand factor A domain containing 1
SEMA4G	semaphorin 4G
CEMIP	cell migration inducing hyaluronidase 1
CDK15	cyclin dependent kinase 15
GNG2	G protein subunit gamma 2
FRMD4B	FERM domain containing 4B
AGER	advanced glycosylation end-product specific receptor
KCNK2	potassium two pore domain channel subfamily K member 2
TNFRSF13C	TNF receptor superfamily member 13C
RND2	Rho family GTPase 2
OLFML3	olfactomedin like 3
ESRRG	estrogen related receptor gamma
FGF18	fibroblast growth factor 18
FGFR2	fibroblast growth factor receptor 2
PAMR1	peptidase domain containing associated with muscle regeneration 1
SEMA4A	semaphorin 4A
CTNND2	catenin delta 2
AJAP1	adherens junctions associated protein 1
SLC9A7P1	solute carrier family 9 member 7 pseudogene 1
FAT2	FAT atypical cadherin 2
HAVCR2	hepatitis A virus cellular receptor 2
RARRES1	retinoic acid receptor responder 1
FBXL13	F-box and leucine rich repeat protein 13
KCNAB3	potassium voltage-gated channel subfamily A regulatory beta subunit 3
FOLR1	folate receptor alpha
C1QTNF3	C1q and TNF related 3
PTP4A3	protein tyrosine phosphatase 4A3
CGB8	chorionic gonadotropin subunit beta 8
GPR39	G protein-coupled receptor 39

BCL2L15	BCL2 like 15
DIRAS2	DIRAS family GTPase 2
KL	klotho
MSC	musculin
MMP19	matrix metallopeptidase 19
CA11	carbonic anhydrase 11
GKN1	gastrokine 1
FOXO6	forkhead box O6
ECEL1P2	endothelin converting enzyme like 1 pseudogene 2
COL8A2	collagen type VIII alpha 2 chain
LINC02881	long intergenic non-protein coding RNA 2881
AMN	amnion associated transmembrane protein
ABCD1	ATP binding cassette subfamily D member 1
HCG22	HLA complex group 22 (gene/pseudogene)
HSD17B8	hydroxysteroid 17-beta dehydrogenase 8
LRFN5	leucine rich repeat and fibronectin type III domain containing 5
SORBS2	sorbin and SH3 domain containing 2
LRRC17	leucine rich repeat containing 17
TMEM130	transmembrane protein 130
KLHL14	kelch like family member 14
NPC1L1	NPC1 like intracellular cholesterol transporter 1
GPR85	G protein-coupled receptor 85
PLCH1	phospholipase C eta 1
SH2D3A	SH2 domain containing 3A
CBLN2	cerebellin 2 precursor
ZNF385B	zinc finger protein 385B
GAL3ST1	galactose-3-O-sulfotransferase 1
ITGB6	integrin subunit beta 6
SLITRK3	SLIT and NTRK like family member 3
CCT6B	chaperonin containing TCP1 subunit 6B
S100A3	S100 calcium binding protein A3
NOS3	nitric oxide synthase 3
NANOS3	nanos C2HC-type zinc finger 3
ST6GALNAC5	ST6 N-acetylgalactosaminide alpha-2,6-sialyltransferase 5
APOC1	apolipoprotein C1
ULK4P3	ULK4 pseudogene 3
HPD	4-hydroxyphenylpyruvate dioxygenase
MEOX1	mesenchyme homeobox 1
SLC22A3	solute carrier family 22 member 3
DBP	D-box binding PAR bZIP transcription factor
GC	GC vitamin D binding protein
HSD17B4	hydroxysteroid 17-beta dehydrogenase 4
DOCK11	dedicator of cytokinesis 11
S100A5	S100 calcium binding protein A5

SLC39A5	solute carrier family 39 member 5
CLEC18A	C-type lectin domain family 18 member A
NBEAP3	neurobeachin pseudogene 3
SOWAHD	sosondowah ankyrin repeat domain family member D
PCDH20	protocadherin 20
CTNNA3	catenin alpha 3
KCNMB4	potassium calcium-activated channel subfamily M regulatory beta subunit 4
PNMA6A	PNMA family member 6A
ISL1	ISL LIM homeobox 1
TNFRSF6B	TNF receptor superfamily member 6b
INHBB	inhibin subunit beta B
SULF1	sulfatase 1
TREH	trehalase
KRTCAP3	keratinocyte associated protein 3
CD4	CD4 molecule
CNTN6	contactin 6
FAM3C2P	family with sequence similarity 3 member C2, pseudogene
CERKL	ceramide kinase like
PCDH18	protocadherin 18
NYAP2	neuronal tyrosine-phosphorylated phosphoinositide-3-kinase adaptor 2
S100A4	S100 calcium binding protein A4
HPGD	15-hydroxyprostaglandin dehydrogenase
SSC4D	scavenger receptor cysteine rich family member with 4 domains
COL24A1	collagen type XXIV alpha 1 chain
CLEC18C	C-type lectin domain family 18 member C
CTSW	cathepsin W
COL3A1	collagen type III alpha 1 chain
RAMP2	receptor activity modifying protein 2
GLI1	GLI family zinc finger 1
ELMOD1	ELMO domain containing 1
EFHB	EF-hand domain family member B
TMEM37	transmembrane protein 37
TNNC1	troponin C1, slow skeletal and cardiac type
TNNI3	troponin I3, cardiac type
ALDH1A1	aldehyde dehydrogenase 1 family member A1
NIPSNAP3B	nipsnap homolog 3B
MAP1LC3C	microtubule associated protein 1 light chain 3 gamma
TMPRSS5	transmembrane serine protease 5
TBX1	T-box transcription factor 1
SOAT2	sterol O-acyltransferase 2
ENPP5	ectonucleotide pyrophosphatase/phosphodiesterase family member 5
ZNF467	zinc finger protein 467
NPY1R	neuropeptide Y receptor Y1
C2orf15	chromosome 2 open reading frame 15

LAG3	lymphocyte activating 3
ENAM	enamelin
ASIC2	acid sensing ion channel subunit 2
ADIRF	adipogenesis regulatory factor
NPFFR2	neuropeptide FF receptor 2
EPHA1	EPH receptor A1
C4B	complement C4B (Chido blood group)
C4A	complement C4A (Rodgers blood group)
FGB	fibrinogen beta chain
GUCY1A1	guanylate cyclase 1 soluble subunit alpha 1
SLC16A9	solute carrier family 16 member 9
OTUD7A	OTU deubiquitinase 7A
SPDYE9	speedy/RINGO cell cycle regulator family member E9
CRB2	crumbs cell polarity complex component 2
SPDYE11	speedy/RINGO cell cycle regulator family member E11
ID4	inhibitor of DNA binding 4
ZIC5	Zic family member 5
TBC1D3C	TBC1 domain family member 3C
SLC25A1P5	solute carrier family 25 member 1 pseudogene 5
TTC39A	tetratricopeptide repeat domain 39A
DLEC1	DLEC1 cilia and flagella associated protein
OLFM5P	olfactomedin family member 5, pseudogene
SLC22A2	solute carrier family 22 member 2
SELENOV	selenoprotein V
KCNJ4	potassium inwardly rectifying channel subfamily J member 4
SMIM6	small integral membrane protein 6
ZBTB45P2	zinc finger and BTB domain containing 45 pseudogene 2
ZNF90P1	zinc finger protein 90 pseudogene 1
FAM131C	family with sequence similarity 131 member C
ULK4P1	ULK4 pseudogene 1
ARHGAP8	Rho GTPase activating protein 8
TIE1	tyrosine kinase with immunoglobulin like and EGF like domains 1
KRT5	keratin 5
PRSS8	serine protease 8
ACKR3	atypical chemokine receptor 3
RAB7B	RAB7B, member RAS oncogene family
THSD7A	thrombospondin type 1 domain containing 7A
TTC22	tetratricopeptide repeat domain 22
PILRA	paired immunoglobin like type 2 receptor alpha
RNF180	ring finger protein 180
GLP2R	glucagon like peptide 2 receptor
GPX7	glutathione peroxidase 7
CLDN7	claudin 7
SRSF12	serine and arginine rich splicing factor 12

BTBD18	BTB domain containing 18
MAP2K6	mitogen-activated protein kinase 6
ANKRD2	ankyrin repeat domain 2
LPAR5	lysophosphatidic acid receptor 5
EPHA6	EPH receptor A6
ZMYND10	zinc finger MYND-type containing 10
C1GALT1C1L	C1GALT1 specific chaperone 1 like
BAALC	BAALC binder of MAP3K1 and KLF4
TECTA	tectorin alpha
ACTG1P10	actin gamma 1 pseudogene 10
AEBP1	AE binding protein 1
RENBP	renin binding protein
TAMALIN	trafficking regulator and scaffold protein tamalin
CCDC85A	coiled-coil domain containing 85A
NRSN1	neurensin 1
SLC5A2	solute carrier family 5 member 2
C1QTNF5	C1q and TNF related 5
MFRP	membrane frizzled-related protein
KCNE1	potassium voltage-gated channel subfamily E regulatory subunit 1
TMEM132B	transmembrane protein 132B
DLX4	distal-less homeobox 4
PAQR9	progestin and adipoQ receptor family member 9
GFRA3	GDNF family receptor alpha 3
NBPF2P	NBPF member 2, pseudogene
PPFIA2	PTPRF interacting protein alpha 2
GPRIN3	GPRIN family member 3
FEZF2	FEZ family zinc finger 2
NCLP1	nucleolin pseudogene 1
SMIM1	small integral membrane protein 1 (Vel blood group)
OBSCN-AS1	OBSCN antisense RNA 1
CYP27C1	cytochrome P450 family 27 subfamily C member 1
RNF175	ring finger protein 175
GYG2	glycogenin 2
SPATA32	spermatogenesis associated 32
LHB	luteinizing hormone subunit beta
SLC6A12	solute carrier family 6 member 12
PCDHGB8P	protocadherin gamma subfamily B, 8 pseudogene
ATP8A1	ATPase phospholipid transporting 8A1
C20orf204	chromosome 20 open reading frame 204
MFNG	MFNG O-fucosylpeptide 3-beta-N-acetylglucosaminyltransferase
MRGPRF	MAS related GPR family member F
UNC5CL	unc-5 family C-terminal like
CASQ1	calsequestrin 1
KDF1	keratinocyte differentiation factor 1

TVP23C-CDRT4	TVP23C-CDRT4 readthrough
PMFBP1	polyamine modulated factor 1 binding protein 1
ASPG	asparaginase
PDE6B	phosphodiesterase 6B
CYP26C1	cytochrome P450 family 26 subfamily C member 1
FOXP3	forkhead box P3
ENPP3	ectonucleotide pyrophosphatase/phosphodiesterase 3
ROBO4	roundabout guidance receptor 4
IL12B	interleukin 12B
ACVRL1	activin A receptor like type 1
LY75-CD302	LY75-CD302 readthrough
ASS1P12	argininosuccinate synthetase 1 pseudogene 12
LTB	lymphotoxin beta
KLHL31	kelch like family member 31
IL20RB	interleukin 20 receptor subunit beta
LIX1	limb and CNS expressed 1
SP8	Sp8 transcription factor
POM121L7P	POM121 transmembrane nucleoporin like 7 pseudogene
MAL	mal, T cell differentiation protein
MRTFA	myocardin related transcription factor A
TIRAP	TIR domain containing adaptor protein
GRIN3A	glutamate ionotropic receptor NMDA type subunit 3A
FREM2	FRAS1 related extracellular matrix 2
STUM	stum, mechanosensory transduction mediator homolog
OMG	oligodendrocyte myelin glycoprotein
UPK3B	uroplakin 3B
SCGB3A2	secretoglobin family 3A member 2
PPEF2	protein phosphatase with EF-hand domain 2
INSYN2B	inhibitory synaptic factor family member 2B
CDK3	cyclin dependent kinase 3
KCNJ5	potassium inwardly rectifying channel subfamily J member 5
MARK1	microtubule affinity regulating kinase 1
APELA	apelin receptor early endogenous ligand
DAND5	DAN domain BMP antagonist family member 5
SPEF1	sperm flagellar 1
RLN2	relaxin 2
ACTG1P9	actin gamma 1 pseudogene 9
CD9	CD9 molecule
CRABP2	cellular retinoic acid binding protein 2
RAB11FIP4	RAB11 family interacting protein 4
SLCO4C1	solute carrier organic anion transporter family member 4C1
SMN1	survival of motor neuron 1, telomeric
IL23A	interleukin 23 subunit alpha
MPZL2	myelin protein zero like 2

ITGB1P1	integrin subunit beta 1 pseudogene 1
SLC4A5	solute carrier family 4 member 5
SLC4A4	solute carrier family 4 member 4
WFDC2	WAP four-disulfide core domain 2
SLC6A16	solute carrier family 6 member 16
BGN	biglycan
ADAMTS9	ADAM metallopeptidase with thrombospondin type 1 motif 9
RSPH4A	radial spoke head component 4A
CNTF	ciliary neurotrophic factor
CCDC181	coiled-coil domain containing 181
ITGB7	integrin subunit beta 7
ENO4	enolase 4
OPN1SW	opsin 1, short wave sensitive
CDH17	cadherin 17
TIMD4	T cell immunoglobulin and mucin domain containing 4
CXXC4	CXXC finger protein 4
VWF	von Willebrand factor
NSUN7	NOP2/Sun RNA methyltransferase family member 7
IGSF10	immunoglobulin superfamily member 10
ZNF662	zinc finger protein 662
FAM110D	family with sequence similarity 110 member D
LPAR4	lysophosphatidic acid receptor 4
DFFBP1	DNA fragmentation factor subunit beta pseudogene 1
WNT10A	Wnt family member 10A
LCK	LCK proto-oncogene, Src family tyrosine kinase
PRSS3	serine protease 3
ZDHHC8BP	ZDHHC8B, pseudogene
SGPP2	sphingosine-1-phosphate phosphatase 2
KIAA0040	KIAA0040
MTCO1P12	MT-CO1 pseudogene 12
FAM163A	family with sequence similarity 163 member A
SLCO2A1	solute carrier organic anion transporter family member 2A1
ZFP91-CNTF	ZFP91-CNTF readthrough (NMD candidate)
PACRG	parkin coregulated
KRT17P7	keratin 17 pseudogene 7
ZNF736P9Y	zinc finger protein 736 pseudogene 9, Y-linked
NPY5R	neuropeptide Y receptor Y5
CEACAM5	CEA cell adhesion molecule 5
FBXW10B	F-box and WD repeat domain containing 10B
ZNF157	zinc finger protein 157
TTC23L	tetratricopeptide repeat domain 23 like
CCDC38	coiled-coil domain containing 38
RPS3AP6	RPS3A pseudogene 6
MEI1	meiotic double-stranded break formation protein 1

PMS2P7	PMS1 homolog 2, mismatch repair system component pseudogene 7
RPL23AP21	ribosomal protein L23a pseudogene 21
HENMT1	HEN methyltransferase 1
PTGDS	prostaglandin D2 synthase
